# Attenuated G protein signaling and minimal receptor phosphorylation as a biochemical signature of low side-effect opioid analgesics

**DOI:** 10.1038/s41598-022-11189-6

**Published:** 2022-05-03

**Authors:** Pooja Dasgupta, Anika Mann, Willma E. Polgar, Rainer K. Reinscheid, Nurulain T. Zaveri, Stefan Schulz

**Affiliations:** 1 Department of Pharmacology and Toxicology, Institute of Pharmacology and Toxicology, Jena University Hospital, Friedrich Schiller University, Drackendorfer Straße 1, 07747 Jena, Germany; 2 Astraea Therapeutics, 320 Logue Avenue, Suite 142, Mountain View, CA 94043 USA

**Keywords:** Biological techniques, Chemical biology, Drug discovery, Molecular medicine

## Abstract

Multi-receptor targeting has been proposed as a promising strategy for the development of opioid analgesics with fewer side effects. Cebranopadol and AT-121 are prototypical bifunctional ligands targeting the nociceptin/orphanin FQ peptide receptor (NOP) and µ-opioid receptor (MOP) that elicit potent analgesia in humans and nonhuman primates, respectively. Cebranopadol was reported to produce typical MOP-related side effects such as respiratory depression and reward, whereas AT-121 appeared to be devoid of these liabilities. However, the molecular basis underlying different side effect profiles in opioid analgesics remains unknown. Here, we examine agonist-induced receptor phosphorylation and G protein signaling profiles of a series of chemically diverse mixed MOP/NOP agonists, including cebranopadol and AT-121. We found that these compounds produce strikingly different MOP phosphorylation profiles. Cebranopadol, AT-034 and AT-324 stimulated extensive MOP phosphorylation, whereas AT-201 induced selective phosphorylation at S375 only. AT-121, on the other hand, did not promote any detectable MOP phosphorylation. Conversely, none of these compounds was able to elicit strong NOP phosphorylation and low NOP receptor phosphorylation correlated with partial agonism in a GIRK-channel assay. Our results suggest a close correlation between MOP receptor phosphorylation and side effect profile. Thus, bifunctional MOP/NOP opioid ligands combining low efficacy G protein signaling at both NOP and MOP with no detectable receptor phosphorylation appear to be devoid of side-effects such as respiratory depression, abuse liability or tolerance development, as with AT-121.

## Introduction

Despite being the gold standard for treatment of severe pain, the widespread and long-term use of opioids is limited by their serious side effects. Chronic opioid use leads to tolerance and dependence, whereas acute ingestion of potent opioids can lead to severe respiratory depression and overdose fatalities. Opioid abuse due to over-prescription and false advertising produced the so-called ‘opioid crisis’ that highlighted the urgent need for developing safer analgesics devoid of the above-mentioned liabilities. The µ-opioid receptor (MOP) mediates all physiological effects of currently used opioid analgesics, including their adverse effects^1^. However, the other members of the opioid receptor family are also known to mediate analgesia. For example, activation of nociceptin/orphanin FQ peptide receptors (NOP) in nonhuman primates results in effective anti-nociception without typical opioid-like side effects such as respiratory depression, itching and reinforcing effects^2^. However, pure NOP agonists might elicit strong sedative effects^3–6^. A novel concept to ameliorate unfavorable side effects is to target multiple opioid receptors simultaneously with a single chemical entity^7–9^. Activation of MOP and NOP pathways in nonhuman primates was demonstrated to produce synergistic antinociceptive effects with an absence of side effects, as was shown with bifunctional MOP/NOP ligands^10–13^. Such a multi-targeted approach could hold the key to improved opioid drugs with minimal opioid liabilities and adverse effects.

Buprenorphine, a clinically used analgesic, displays a unique pharmacology, as it has mixed agonist–antagonist effects at all the opioid receptors. As a result, it exhibits a “ceiling-effect” on respiratory depression; however, due to its significant MOP agonism buprenorphine is not completely devoid of reinforcing effects^14^. Cebranopadol, a novel pan-opioid analgesic was developed with the goal of achieving a higher therapeutic index than the prototypical marketed MOP-targeted opioids. It is a potent analgesic that has high efficacy at both NOP and MOP receptors^15^. Although initially cebranopadol showed anti-allodynic and anti-hyperalgesic effects in rodent neuropathic pain models, it is not completely devoid of typical opioid-like adverse effects^16–18^. Another promising MOP/NOP bifunctional candidate, AT-121, has partial agonist efficacy at both MOP and NOP receptors^13^. Besides potent analgesic effects of AT-121 in nonhuman primates, the absence of physical dependence or opioid-induced hyperalgesia supports the therapeutic potential of this novel compound. Additionally, its ability to attenuate oxycodone’s reinforcing effects is another attractive feature. Altogether, these studies along with the diminishing focus on biased MOP agonists emphasize the therapeutic potential of developing bifunctional drugs targeting both MOP and NOP as novel analgesics lacking opioid liabilities. However, there is limited knowledge regarding in vitro biochemical signatures that correlate with an optimal balance between MOP and NOP receptor activities, and which of these have predictive value for a reduced side-effect profile. In essence, we aim to formulate a hypothesis for the design of safer opioid analgesics devoid of adverse effects, based on in vitro biochemical parameters as proxies.

Here, we make use of phosphosite-specific antibodies^19–21^ and a G protein-coupled inwardly rectifying potassium (GIRK)-channel based fluorescent screening assay^22–24^, two highly sensitive and reliable tools to characterize the in vitro pharmacological profile of novel MOP/NOP bifunctional ligands. Although all these compounds can principally be classified as mixed MOP/ NOP agonists, it is important to determine the ideal balance between MOP and NOP mediated effects. Phosphosite-specific antibodies for the NOP receptor C-terminal residues S351 and T362/S363 and the MOP receptor residues T370, S375, T376 and T379 have revealed agonist-induced receptor phosphorylation as a critical event in NOP and MOP receptor signaling^19–21^. Agonist-dependent opening of GIRK channels is also an endogenous effect of opioids and reflects G protein signaling. These analyses are complemented with measures for radioligand binding and GTPγ^35^S binding as an indicator of non-amplified GPCR activation. We apply this strategy to a series of new bifunctional MOP/NOP compounds and compare them to the two prototypical bifunctional agonists cebranopadol and AT-121. For this, we studied novel MOP/NOP bifunctional agonists AT-034, AT324 and AT-201 each having a distinct binding and selectivity profile at NOP and MOP. Although they have been characterized in binding studies, their biochemical profiles have not been investigated in detail before. We suggest that multisite receptor phosphorylation provides a unique biochemical code for each agonist, which along with G protein signaling assays, could be a tool to analyze the effects of chemically distinct novel mixed MOP/NOP agonists. Our results support the hypothesis that a combination of partial agonism at both MOP and NOP in G protein signaling, together with absence of agonist-induced receptor phosphorylation, may be characteristic of NOP- and MOP-targeted opioids that have a safer therapeutic profile and minimal side effects.

## Methods

### Drugs

The nociceptin/orphanin FQ (N/OFQ) peptide and DAMGO peptide were purchased from Abcam (Cambridge, UK) and Sigma-Aldrich (Steinheim, Germany), respectively. AT-121, AT-034, AT-201 and AT-324 were synthesized at Astraea Therapeutics (Mountain View, CA, USA)^13,25–27^. The AT compounds were dissolved in DMSO and stored at a stock concentration of 10 mM at − 20 °C. Cebranopadol was purchased from MedChemExpress (Germany).

### Cell culture, transfection and fluorescence activated cell sorting

Human embryonic kidney 293 (HEK293) cells and AtT-20 cells were obtained from DSMZ (Braunschweig, Germany) and ATCC (Manassas, USA) respectively, and cultured in Dulbecco´s modified Eagle´s medium (DMEM) supplemented with 10% fetal bovine serum (FBS), 100 U/mL penicillin/streptomycin, 2 mM l-glutamine in a 5% CO_2_ incubator at 37 °C. Cells were transfected using Turbofect (ThermoFisher Scientific, Schwerte, Germany) according to the manufacturer’s instructions. Cells were transfected with plasmids encoding human (h) NOP (imaGenes) or hMOP receptor (Eurofins) with an additional N-terminal HA- or FLAG-tag, respectively. Transfectants were selected with geneticin 400 µg/mL or puromycin 0.1 µg/mL. An additional enrichment of positively expressing cells was done using fluorescence-activated cell sorting as previously described^21,24^. Radioligand displacement competition binding and GTPγ^35^S binding functional assays were conducted in Chinese hamster ovary (CHO) cells stably expressing human NOP and MOP opioid receptors individually, as reported previously^8,28^. The NOP CHO cells were cultured in 150 mm tissue culture dishes (Corning, New York City, NY) in Dulbecco’s Modified Eagle’s Medium (DMEM, Gibco, Thermo Fisher Scientific, Waltham, MA) supplemented with 10% fetal bovine serum (FBS), 100 U/mL penicillin, 100 μg/mL streptomycin and 400 μg/mL G418. MOP CHO cells were cultured in 50% F12/DMEM (Gibco, Thermo Fisher Scientific, Waltham, MA) supplemented with 10% FBS, 100 U/mL penicillin, 100 μg/mL streptomycin and 400 μg/mL G418.

### Membrane preparation

Human NOP and human MOP CHO cells were grown to confluency and harvested for membrane preparation. Membranes were prepared as described^29^, in 50 mM Tris buffer (pH 7.4). Cells were scraped off the culture dishes and centrifuged at 500×*g* for 15 min. The cell pellet was homogenized in 50 mM Tris with a Fisher Scientific PowerGen 125 rotor–stator type homogenizer, centrifuged at 20,000×*g* for 25 min, washed and re-centrifuged once more at 20,000×*g* for 25 min, and aliquoted at a concentration of 2 mg/mL protein per vial for NOP and 3 mg/mL protein per vial for MOP, and stored in a − 80 °C freezer until use.

### Receptor binding

AT-compounds were dissolved in 100% DMSO to a concentration of 10 mM. The binding assays were performed in 96-well polystyrene plates using six concentrations of each test compound (1 μM–0.01 nM) in triplicate, by adding 100 μL of compound and 100 μL of tritiated ligands [^3^H]DAMGO (48.0 Ci/mmol, K_d_ 0.69 nM for MOP) or [^3^H]N/OFQ (130 Ci/mmol, K_d_ 0.065 nM for NOP). Nonspecific binding was determined using 1.0 μM of unlabeled N/OFQ for NOP and 1.0 μM of unlabeled DAMGO for MOP. Assays were initiated by the addition of 800 μL of membrane per well, after which the samples were incubated for 60 min at 25 °C in a total volume of 1.0 mL. In NOP receptor experiments, 1 mg/mL BSA was added to the compound dilution buffer. The incubation was terminated by rapid filtration through 0.05% PEI-soaked glass fiber filter mats (GF/C Filtermat A, Perkin-Elmer) on a Tomtec Mach III cell harvester and washed 5 times with 0.5 mL of ice-cold 50 nM Tris–HCl (pH 7.4) buffer. The filters were dried overnight and soaked with scintillation cocktail before counting on a Wallac Beta plate 1205 liquid scintillation counter. Radioactivity was determined as counts per minute (CPM). IC_50_ values were determined using at least six concentrations of test compound, and calculated using GraphPad/Prism (ISI, San Diego, CA). K_i_ values were determined by the method of Cheng and Prusoff^30^.

### [^35^S] GTPγS binding assay

[^35^S] GTPγS binding assays were conducted as previously described^8^. In brief, membranes (2 mg/mL protein for NOP and 3 mg/mL for MOP) were incubated for 60 min at 25 °C with [^35^S] GTPγS (50 pM), GDP (10 μM), and the appropriate compound, in a total volume of 1.0 ml Buffer A containing 20 mM HEPES, 10 mM MgCl_2_, and 100 mM NaCl (pH 7.4). Samples were filtered over glass fiber filters and bound radioactivity was counted as described for the binding assays. To calculate the efficacy (% stimulation), the amount of stimulation induced by test compounds was normalized to that of the positive controls N/OFQ or DAMGO set at 100%.

### GIRK assay

AtT-20 cells stably expressing MOP or NOP receptor were plated in 96-well black, clear bottom plates coated with poly-l-lysine. Plates were kept for 48 h at 37 °C and 5% CO_2_. Assays were performed as previously described using Hank’s balanced salt solution (HBSS), buffered with HEPES 20 mM (pH7.4) as standard buffer^24^. The fluorescent membrane potential dye FMP (FLIPR Membrane Potential kit BLUE, Molecular Devices, Biberach, Germany) was reconstituted according to the manufacturer´s instructions. Test compounds were prepared right before assay measurements at tenfold higher concentration than indicated. The assay was performed in a FlexStation 3 microplate reader (Molecular Devices). Halfmaximal effective doses (EC_50_) were calculated from assay read-outs obtained in duplicates on 3 separate occasions using OriginLab software (Northampton, MA, USA) as described^24,31^.

### Phosphosite-specific antibodies

The phosphosite-specific MOP antibodies pT370-MOP (7TM0319B), pS375-MOP (7TM0319C), pT376-MOP (7TM0319D), pT379-MOP (7TM0319E) and phosphosite-specific NOP antibodies pS346-NOP (7TM0320A), pS351-NOP (7TM0320B), pT362/pS363-NOP (7TM0320C) as well as the phosphorylation-independent antibodies non-phospho-MOP (7TM0319N), non-phospho-NOP (7TM0320N) and rabbit polyclonal anti-HA antibodies (7TM000HA) were provided by 7TM Antibodies (Jena, Germany). All antibodies have been extensively characterized previously^19–21,32^. Phosphosite-specific antibodies were affinity-purified against their immunizing phosphorylated peptides.

### Western Blot analysis

Stably transfected HEK293 cells were seeded onto poly-l-lysine-coated 60 mm dishes and grown to 80% confluency. After compound-treatment, cells were lysed in detergent buffer in the presence of protease (Complete Mini) and phosphatase (PhosSTOP) inhibitors (Sigma-Aldrich, Steinheim, Germany). Glycosylated MOP was enriched using wheat germ lectin-agarose (WGA) beads and NOP receptor was enriched using HA-beads (ThermoFisher Scientific, Schwerte, Germany) as described in detail^19,21,33,34^. Proteins were eluted from the beads using SDS-sample buffer for 25 min at 43 °C (MOP receptor) or 30 min at 50 °C (NOP receptor). After SDS–polyacrylamide gel electrophoresis and electroblotting, membranes were incubated with the MOP receptor phosphosite-specific antibodies anti-pT370 [3196], anti-pS375 [2493], anti-pT376 [3723], anti-pT379 [3686] or with the NOP receptor phosphosite-specific antibodies anti-S346 [5034], anti-pS351 [4876], anti-pT362/S363 [4874], followed by detection using an enhanced chemiluminescence detection system (ThermoFisher Scientific, Schwerte, Germany). Blots were stripped and incubated with the phosphorylation-independent anti-MOP [UMB3] or anti-NOP receptor antibody [4871] to ensure equal loading of the gels. For a semi-quantitative study the western blot images were analyzed using ImageJ 1.47v software (National Institute of Health, Bethesda, MD, USA).

### Statistical analysis

Receptor binding, dose–response relationships for GTPγ^35^S binding and GIRK channel activation were calculated from at least three independent experiments conducted in triplicate or duplicates, using the indicated software. Maximal activation values (E_max_) were calculated by normalizing to values obtained with 1 µM prototypical agonist (DAMGO for MOP, N/OFQ for NOP) set at 100%. Phosphorylation intensities were normalized to levels obtained with 10 µM prototypical agonist and calculated from triplicate incubations repeated at least three times. EC_50_ values are given as means ± SEM.

## Results

**Figure 1:**
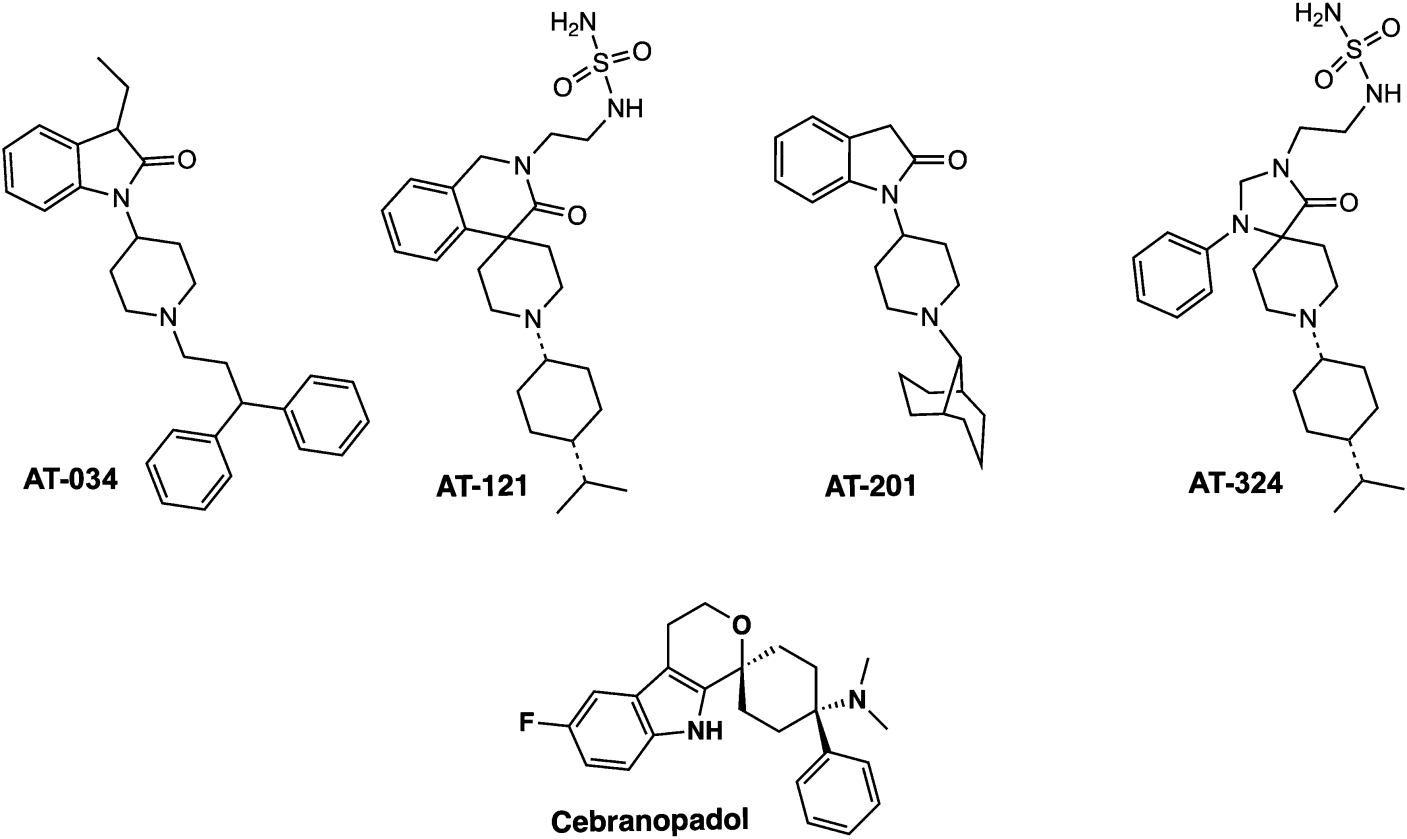
Chemical structures. Chemical structures of investigated novel mixed MOP/NOP agonists.

**Figure 2:**
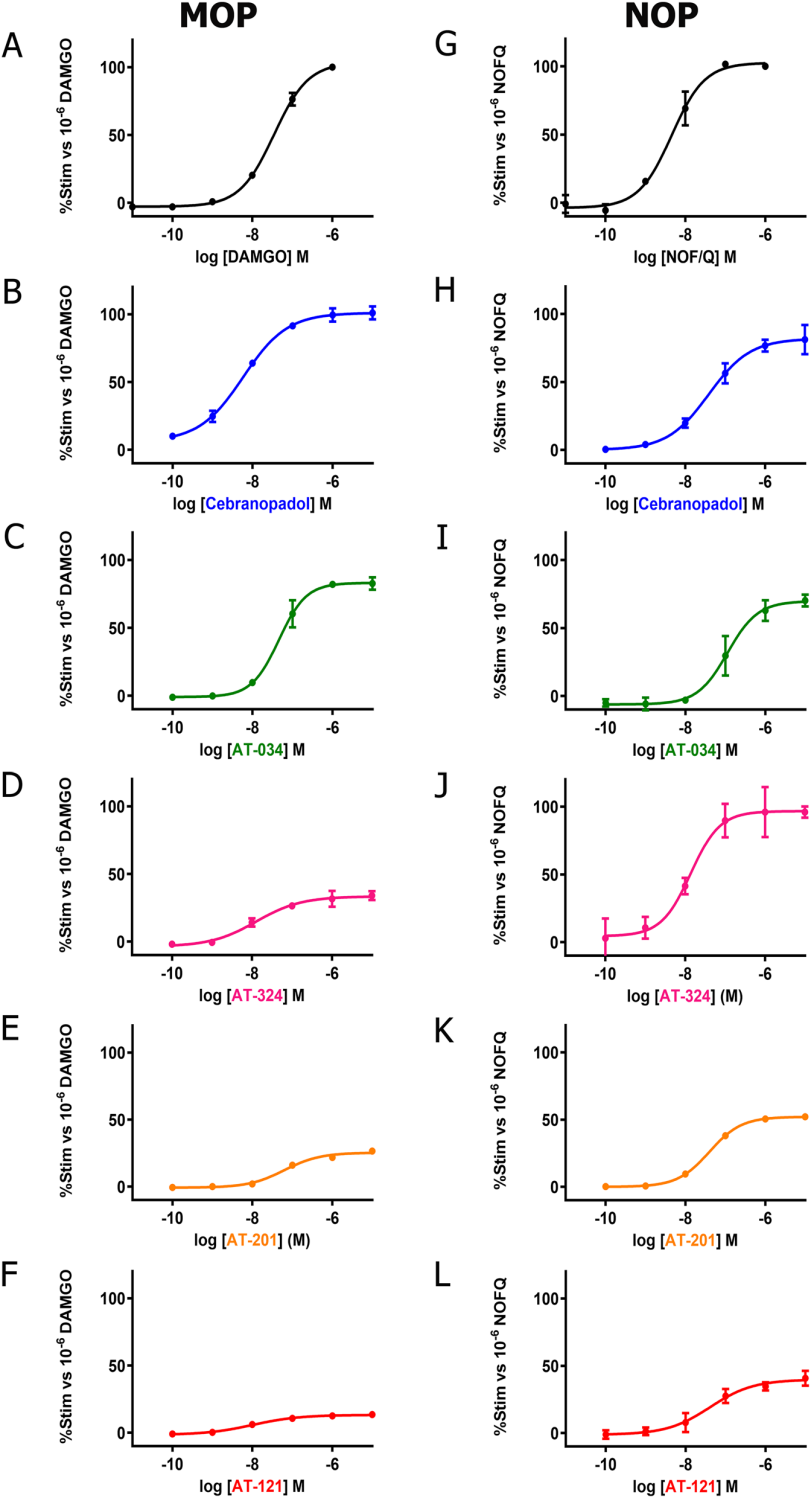
G protein signaling of novel mixed MOP/NOP agonists measured by GTPƔS assay. Concentration–response curves obtained for prototypical selective agonist DAMGO (**A**) and N/OFQ (**G**), novel mixed MOP/NOP agonists cebranopadol (**B**, **H**), AT-034 (**C**, **I**), AT-324 (**D**, **J**), AT-201 (**E**, **K**), AT-121 (**F**, **L**) at MOP and NOP, respectively, in stably expressing CHO cells. Each data point (mean ± SEM) was calculated from at least three independent experiments conducted in triplicates.

Chemical structures of the novel AT compounds are depicted in Fig. 1. In vitro binding affinities of the compounds at NOP and MOP were determined using competitive radioligand displacement^35^. As shown in Table 1, cebranopadol, AT-121, AT-201 and AT-324 have a high affinity at NOP, whereas AT-034 shows moderate affinity. In contrast, all AT compounds and cebranopadol have high affinity at MOP. Functional efficacy at NOP and MOP was determined in the [^35^S] GTPɣS binding assay using membranes from CHO cells transfected with the respective human receptors. Agonist efficacy (E_max_) of the compounds was determined from their relative [^35^S] GTPɣS binding normalized to maximal effects elicited by the selective full agonists N/OFQ and DAMGO at NOP and MOP, respectively (Fig. 2**)**. At NOP, only AT-324 displayed full intrinsic activity (Table 2). Cebranopadol and AT-034 showed high intrinsic activity although with lower potency than AT-324. AT-201 and AT-121 displayed partial agonist efficacy at NOP. Conversely, at MOP, cebranopadol and AT-034 exhibited full intrinsic activity, whereas AT-324 and AT-201 had partial agonist efficacy. AT-121 displayed lower intrinsic activity at MOP compared to AT-201 but has similar agonist potency.

**Table 1:**
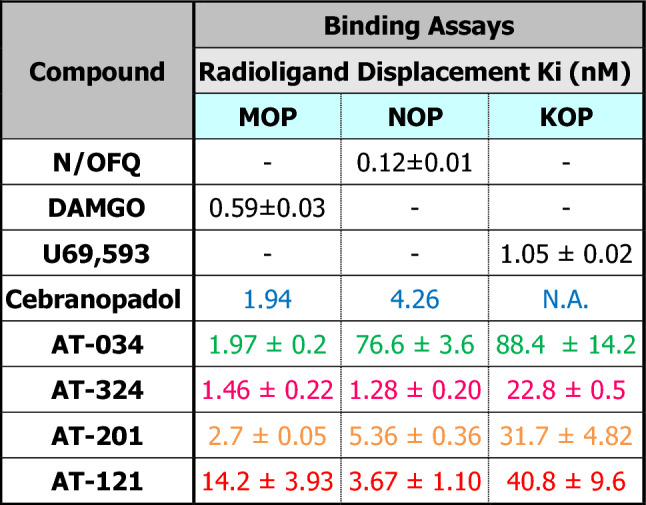
Receptor binding of the investigated compounds in CHO cells stably expressing MOP, NOP and KOP.

**Table 2:**
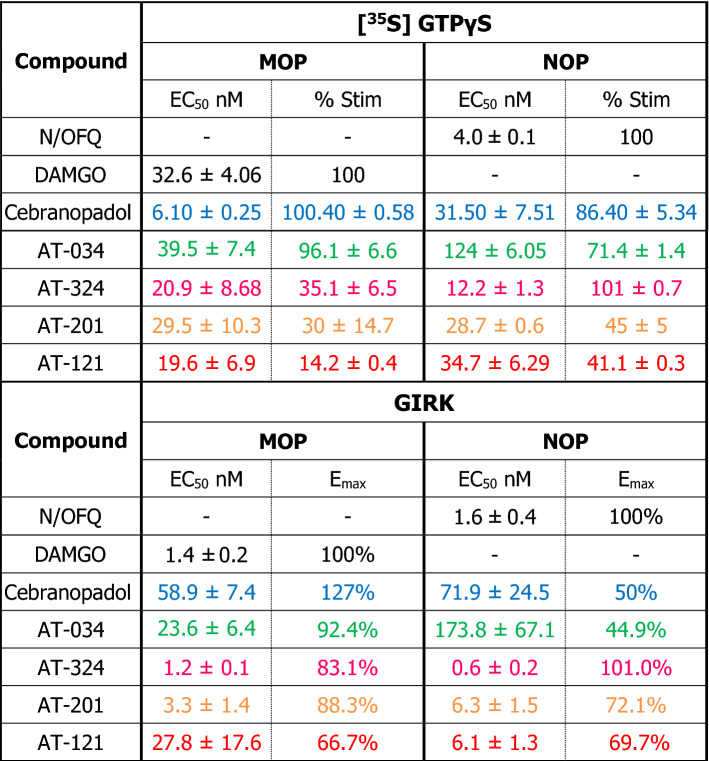
Stimulated [^35^S] GTPγS binding in stably transfected CHO cells and GIRK assay in stably transfected AtT-20 cells.

%Stim, %stimulation.

**Figure 3:**
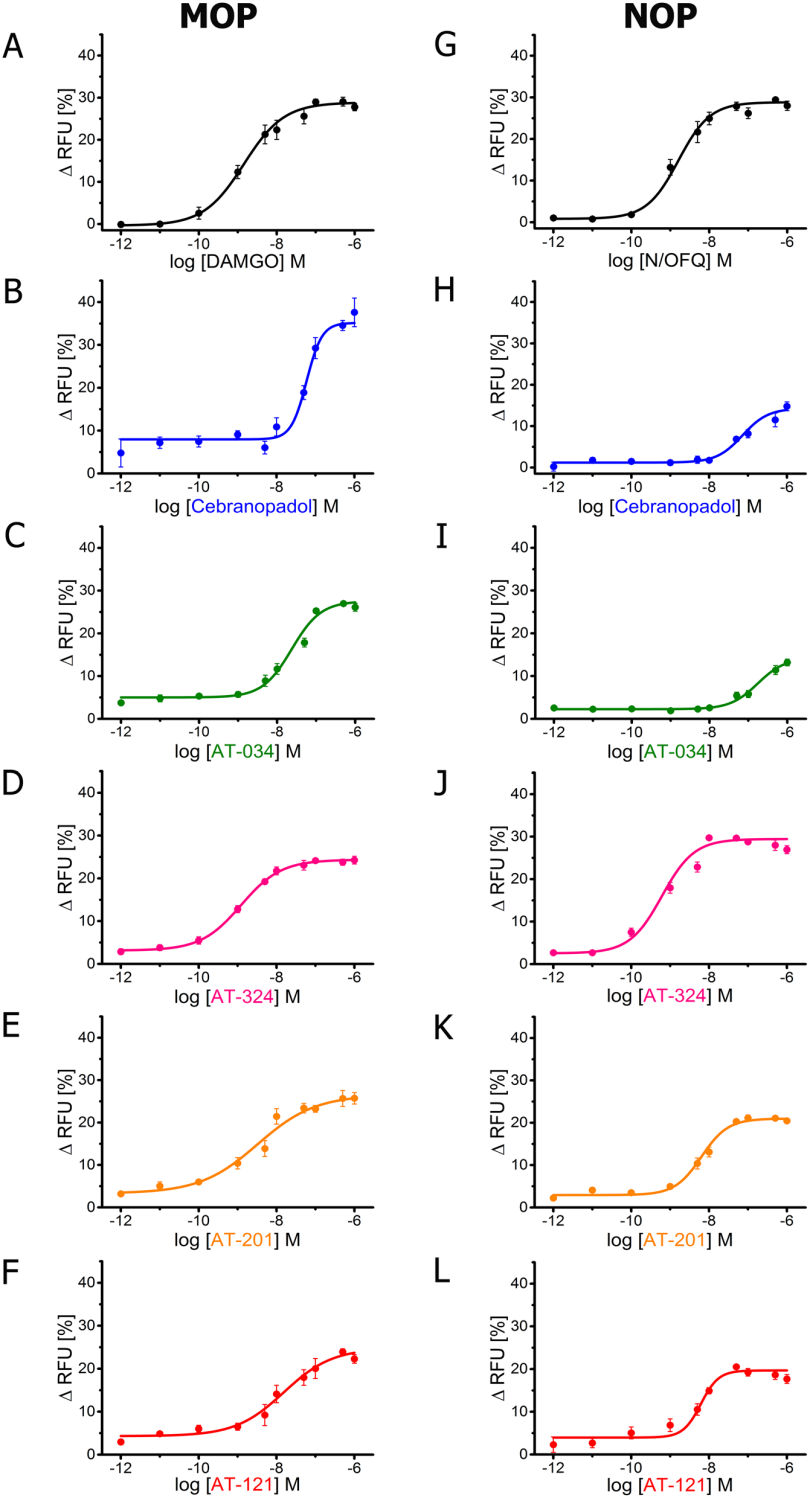
G protein mediated effects of chemically diverse mixed MOP/NOP agonists using GIRK assay. Concentration–response curves obtained for prototypical selective agonist DAMGO (**A**) and N/OFQ (**G**), novel mixed MOP/NOP agonists cebranopadol (**B**, **H**), AT-034 (**C**, **I**), AT-324 (**D**, **J**), AT-201 (**E**, **K**), AT-121 (**F**, **L**) at MOP and NOP, respectively. AtT-20 cells stably expressing MOP and NOP were stimulated with the agonists at a concentration range of 10^–6^ to 10^–12^ M and the signal was measured using GIRK channel-based membrane potential assay. Each data point (mean ± SEM) represents assay read-out obtained from four different experiments in duplicates. (ΔRFU, change in relative fluorescence unit).

We further investigated functional efficacy of the compounds using a GIRK channel-based fluorescent screening assay in AtT-20 cells expressing NOP or MOP, as an additional read-out for G protein signaling. All compounds produced a concentration-dependent change in the membrane potential which was recorded as a change in fluorescent signal of the FMP dye. E_max_ was determined by normalizing the maximal change in fluorescent signal of each compound to that of standard full agonists at the respective receptors (Table 2). Both N/OFQ and DAMGO yielded EC_50_ values in the nanomolar range at NOP and MOP, respectively (Fig. 3). Corresponding to results obtained in GTPɣS binding assay, AT-324 displayed full intrinsic activity at NOP whereas AT-201, AT-121, cebranopadol and AT-034 showed partial agonist efficacy at NOP. At MOP, cebranopadol showed superagonist activity in our hands, with an E_max_ of 127% compared to DAMGO. AT-034, AT-201 and AT-324 displayed high agonist efficacy while AT-121 showed the lowest E_max_ at MOP in the GIRK assay compared to the other bifunctional ligands.

**Figure 4:**
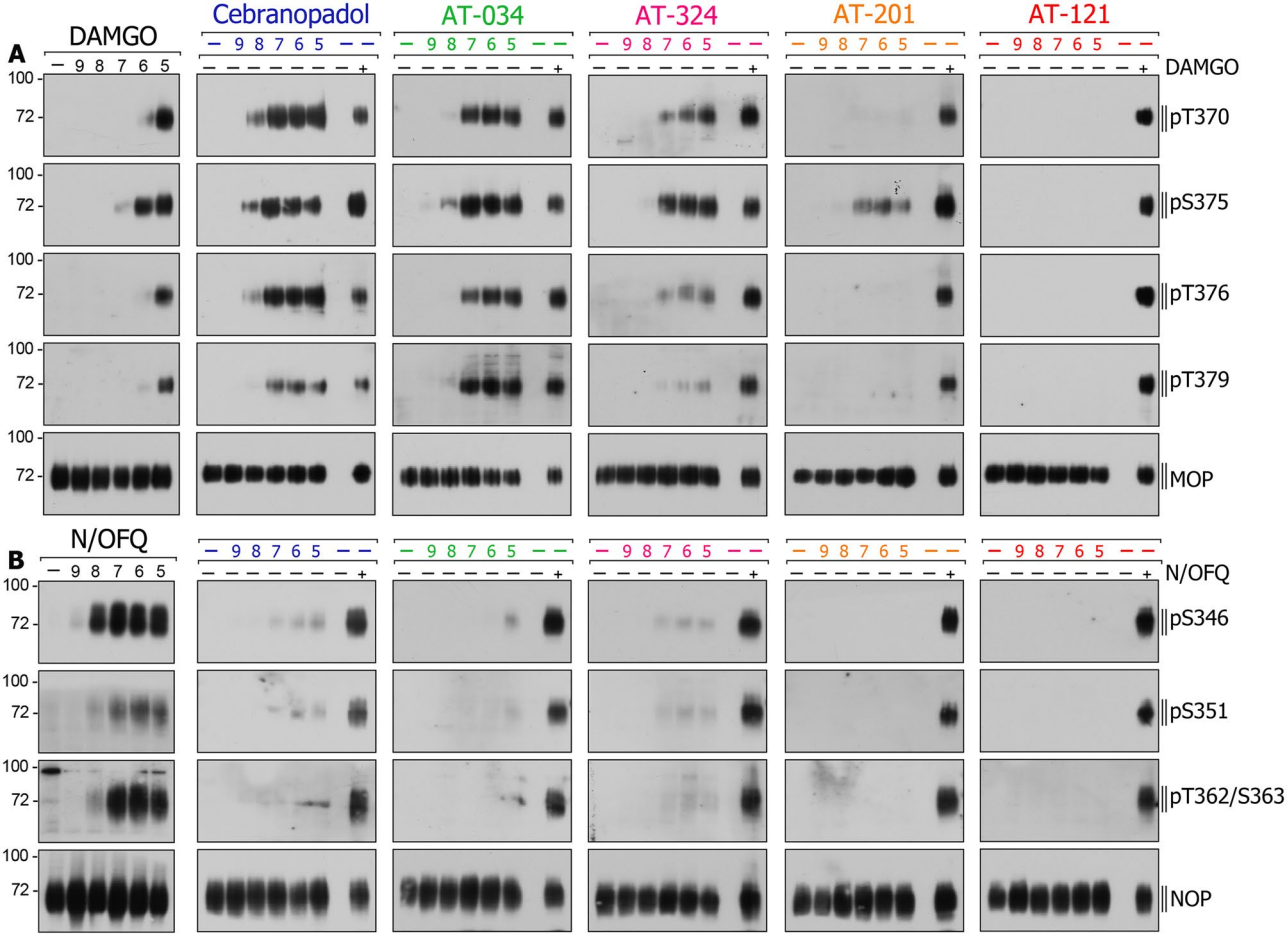
Agonist-induced receptor phosphorylation at MOP (**A**) and NOP (**B**). Stably transfected MOP and NOP cells were stimulated with the agonists at a concentration range of 10^–5^ to 10^–9^ M. The lysates were immunoblotted with MOP phoshosite-specific antibodies pT370, pS375, pT376 and pT379 and NOP phosophosite-specific antibodies pS346, pS351, pT362/S363, respectively. Blots were stripped and reprobed with anti-MOP or anti-NOP antibody. Each blot is a representative image of n = 3 independent experiments. Original blots are shown in Supplementary Fig. 1.

**Figure 5:**
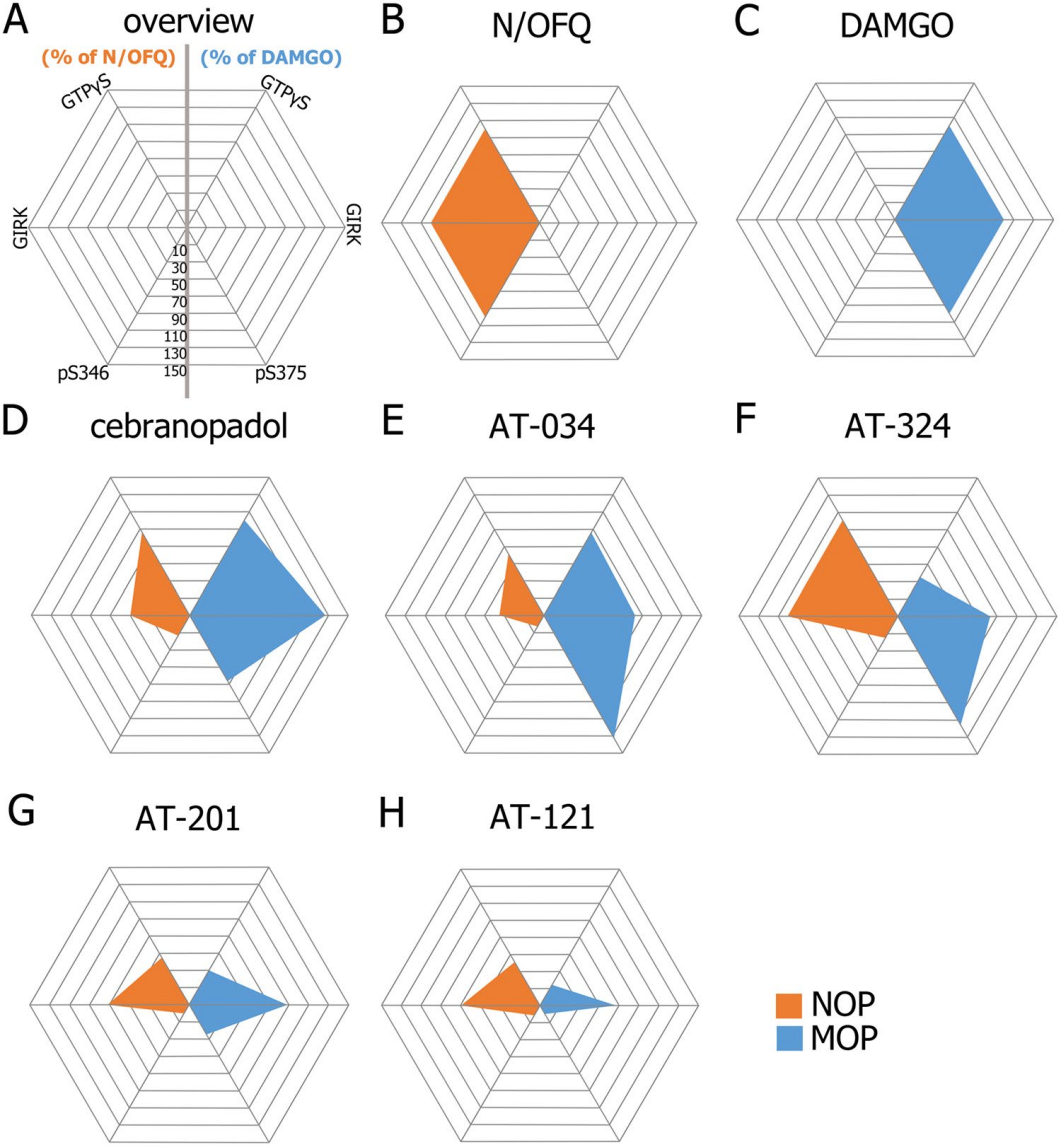
Radar graphs illustrating the balance between agonist-mediated effects of the investigated compounds at NOP (orange) and MOP (blue). All axes represent values normalized to maximal concentration of N/OFQ or DAMGO set at 100%. Phosphorylation values were calculated for the primary phosphorylation site S346 at NOP and S375 at MOP. Scale ranging from − 10 to 150% with an interval of 20%.

Next, we studied agonist-induced receptor phosphorylation at NOP and MOP in stably transfected HEK293 cells (Fig. 4). At MOP, cebranopadol produced robust phosphorylation at T370, S375, T376 and moderate phosphorylation at T379 in a concentration-dependent manner. On the contrary, cebranopadol failed to elicit phosphorylation at NOP, except for very weak phosphorylation at the primary site S346. AT-034 induced robust phosphorylation at T370, S375, T376 as well as T379 in MOP, whereas no phosphorylation was seen at NOP. AT-324 elicited strong phosphorylation at T370 and S375 at MOP but was barely able to stimulate detectable phosphorylation at NOP. Conversely, AT-201 elicited phosphorylation only at S375 at MOP; however, no phosphorylation was detected at NOP. AT-121 was the only compound that failed to induce any phosphorylation at MOP or NOP even at higher concentrations. In summary, cebranopadol and AT-121 show major differences in their in vitro profile with respect to G protein signaling and receptor phosphorylation (Fig. 5).

## Discussion

Various strategies have emerged in medicinal chemistry over the past few years to address the adverse side-effects associated with the use of MOP-targeted opioids. These include biased MOP agonists, multi- or bi-functional ligands, positive allosteric modulators, or partial agonists; yet, a consensus strategy remains a significant challenge^7^. Biased agonism seemed to be a promising concept initially, however, clinical trials with the presumed G protein biased agonist TRV130 (oliceridine) failed to produce therapeutic results devoid of side-effects such as constipation^36^ or respiratory depression, although respiratory effects appeared lower than in the morphine control group^37^. Another approach that shows substantial potential is co-activation of NOP and MOP receptors. Several studies indicate that activation of NOP can attenuate opioid-mediated adverse effects such as reinforcing and rewarding behaviors^38–40^. Moreover, co-activation of NOP and MOP has been shown to produce synergistic enhancement of MOP-mediated anti-nociception^10^.

The compounds used in this study, AT-034, AT-121, AT-201, and AT-324 are bifunctional NOP-MOP ligands belonging to different chemical scaffolds^41^ (Fig. 1), designed using medicinal chemistry strategies to have varying levels of NOP and MOP binding affinities and intrinsic efficacies^13,35,42,43^. AT-034 and AT-201 are from a series of piperidinyl-1,3-dihydroindolone-based NOP ligands whose chemical modifications resulted in varying affinity at the MOP receptor and differential pharmacological effects. AT-201 (previously called SR16435), which shows high binding affinity at NOP and MOP and has partial agonist efficacy at both receptors, shows naloxone-reversible antinociceptive activity in mice but also showed naloxone-reversible rewarding effects in the conditioned place preference model in mice^44,45^. However, compared with morphine, AT-201 showed lower tolerance development to its antinociceptive effects after chronic treatment, attributed to its NOP efficacy and bifunctional MOP/NOP profile^44,45^. The piperidinyl-indolinone AT-034, on the other hand, has higher binding affinity at MOP than at NOP (Table 1), and higher agonist efficacy at MOP than at NOP (Table 2). AT-034 was shown to significantly reduce cocaine self-administration in rats, similar to buprenorphine, an effect that required co-activation of both MOP and NOP receptors^46^.

AT-121 belongs to a novel class of spiro-isoquinolinone NOP ligands which were optimized using structure-based drug design to produce a MOP/NOP bifunctional ligand with partial agonist efficacy at both receptors^13^. In nonhuman primates, AT-121 produced potent anti-nociceptive and anti-hyperalgesic effects devoid of reinforcing effects or physical dependence. In addition, AT-121 did not compromise respiratory or cardiovascular functions functions at supra-analgesic doses and showed significantly lower tolerance development compared to morphine after chronic treatment in nonhuman primates^13^. AT-324 belongs to yet a different chemical scaffold, the triazaspirodecanone scaffold, which has yielded highly selective NOP ligands such as the selective NOP receptor agonist too compound Ro64-6198^4^. While AT-324 has similarly high binding affinity at NOP as Ro64-6198, medicinal chemistry-driven structural modifications of this scaffold afforded high MOP binding affinity and a bifunctional MOP/NOP agonist efficacy profile in vitro^41^, shown in Tables 1 and 2. AT-324 also shows potent antinociceptive activity in nonhuman primates (unpublished results). As a comparator, we used cebranopadol, which is now well-characterized for its pharmacological effects in various animal models as well as in human clinical studies^47^. Cebranopadol belongs to a class of spiro[cyclohexane-dihydropyrano[3,4-b]indol]-amines and is a potent NOP and MOP agonist^15^, chemically distinct from the AT-series of compounds. It produced potent analgesic effects in various rat models of acute and chronic pain^15,16^. Although it did not elicit respiratory or motor deficits, or itching, it produced reinforcing effects in nonhuman primates^48,49^. Recent clinical studies showed that cebranopadol is effective in treatment of post-operative pain, cancer-related pain, as well as lower back pain^47,50–52^.

In this study, we assessed the in vitro pharmacological effects of these chemically diverse novel mixed MOP/NOP agonists on agonist-induced G protein signaling and receptor phosphorylation. In addition to the standard ligand binding and GTP_Ɣ_S assays, we used two novel pharmacological measures, i.e., GIRK channel-based fluorescent assay and phosphosite-specific antibodies, to study different stages of agonist-induced receptor activation in vitro. The GIRK channel-based membrane potential assay is a reliable method that uses a fluorescent membrane potential (FMP) dye to study G protein signaling^24^. The role of GIRK channels as a key effector protein in signaling of receptors involved in pain pathways has been demonstrated in various animal models^53–55^. Receptor phosphorylation and its functional importance in GPCR regulation and desensitization have been extensively demonstrated^56,57^. Phosphosite-specific antibodies have proven to be reliable tools to study temporal and spatial aspects of receptor regulation. We have previously characterized phosphosite-specific antibodies that effectively detect phosphorylation at the carboxyl terminus of their respective G protein-coupled receptors. These include pT370, pS375, pT376 and p T379 at MOP and pS346, pS351 and pT362/S363 at NOP receptors^19–21^.

AT-034, which could be described as a ‘MOP-dominant’ MOP/NOP bifunctional agonist (i.e. intrinsic activity at MOP > intrinsic activity at NOP) from our assays (Fig. 5), elicited strong phosphorylation at pT370, S375, T376 and T379 residues at the C-terminal of MOP but showed no phosphorylation at NOP (Fig. 4). Although AT-324 was more ‘NOP-dominant’ in G protein signaling assays, it induced robust phosphorylation of MOP, especially at S375, but no phosphorylation of NOP at any of the tested sites. In contrast, AT-201 is a ‘balanced’ MOP/NOP agonist (MOP intrinsic activity ≅ NOP intrinsic activity) that induced only S375 phosphorylation at MOP, but none at NOP. Along with its analgesic effects, it also displayed rewarding properties in mice^44^. Interestingly, clinically used buprenorphine which shows partial agonism at both MOP and NOP, has a phosphorylation profile similar to AT-201 wherein it stimulates only S375 phosphorylation at MOP but none at NOP^20,21^ Cebranopadol showed a more ‘MOP-dominant’ profile and partial to high efficacy at NOP in G protein signaling assays. Despite its clinical efficacy in pain, cebranopadol has been reported to produce reinforcing effects in rats, nonhuman primates and produced drug liking in human clinical trials^48,58,59^. Correspondingly, it elicited strong multi-site phosphorylation at MOP and weak NOP phosphorylation at S346. Interestingly, AT-121 which is a partial agonist at both MOP and NOP did not induce any detectable phosphorylation signal at either of the receptors and only AT-121 was reported to be devoid of any opioid-related side-effects in a nonhuman primates^13^ unlike AT-201 and cebranopadol^44,60^.

Our current studies expand and reinforce the hypothesis that bifunctional MOP/NOP compounds with lower intrinsic activity at both targets may have promising profiles with wider therapeutic windows and reduced adverse effects^49^. Recent findings that suggest low-efficacy partial agonism at MOP as an approach to design safer analgesics further support this hypothesis^61,62^. Altogether, the two parameters of (1) partial agonism in G protein signaling at MOP and NOP, combined with (2) low or absent agonist-induced receptor phosphorylation, appear to have predictive validity for favorable therapeutic effects avoiding abuse liability. However, the optimal balance of NOP- or MOP-related effects for development of novel and safe opioids remains to be examined in more detail.

## Supplementary Information

Supplementary Information.
