# Supplementary material for: Attenuated G protein signaling and minimal receptor phosphorylation as a biochemical signature of low side-effect opioid analgesics
Source: Sci Rep. 2022 May 3;12:7154. doi: 10.1038/s41598-022-11189-6 (PMC9065038; doi:10.1038/s41598-022-11189-6)
Supplement: Supplementary file 1 — Supplementary Information. [file 41598_2022_11189_MOESM1_ESM.pdf]

AT-034

NOP

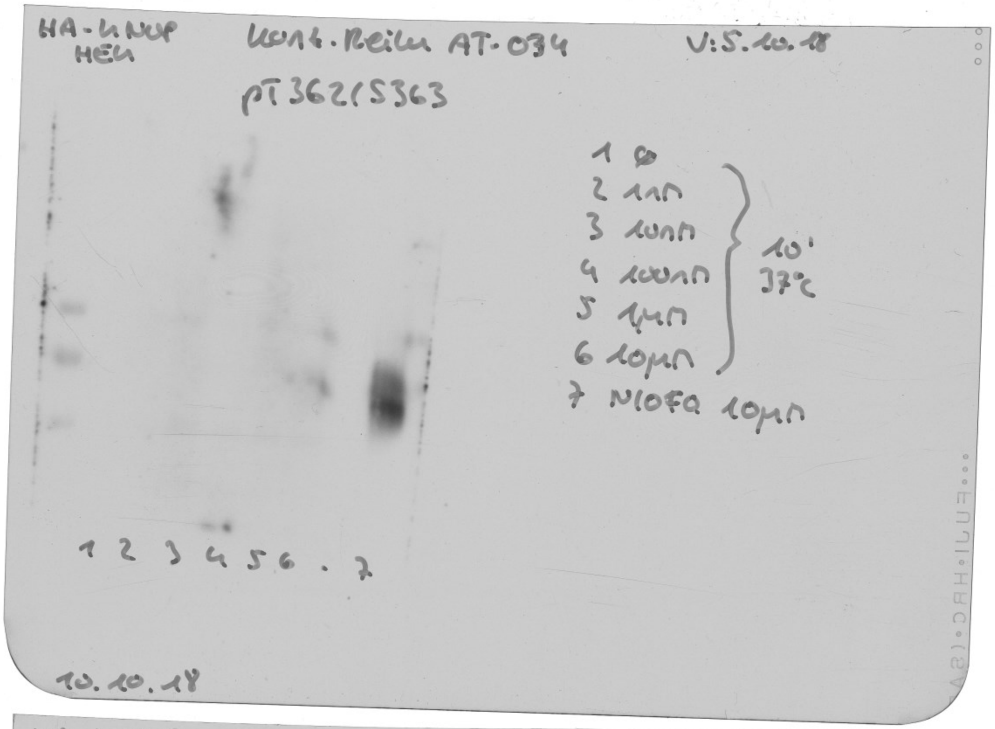

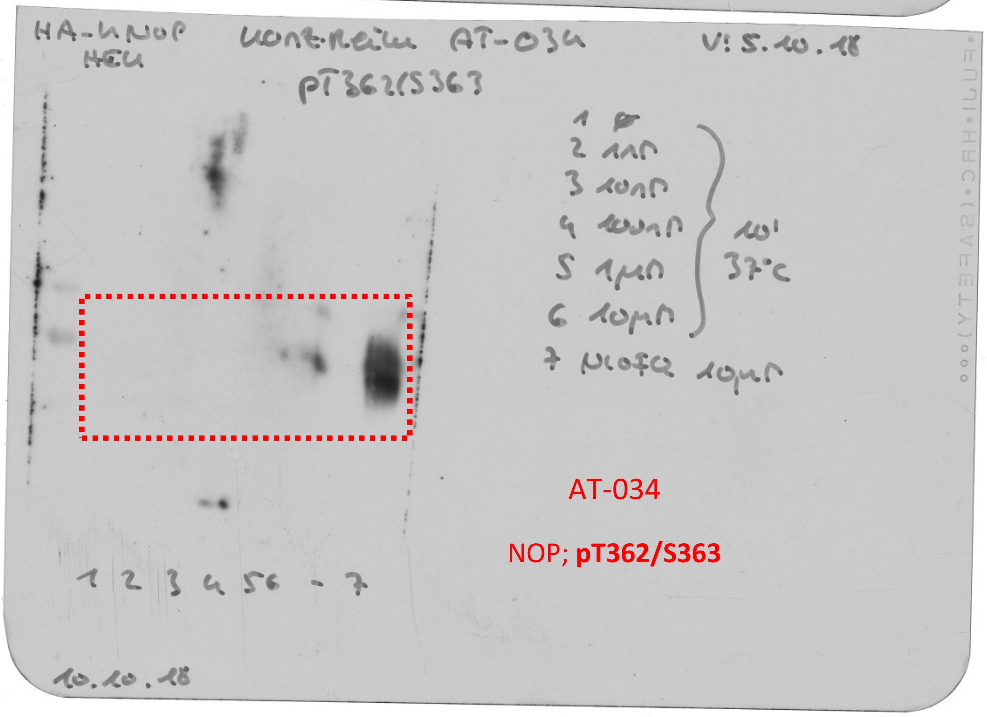

AT-034

NOP; **pT362/S363**

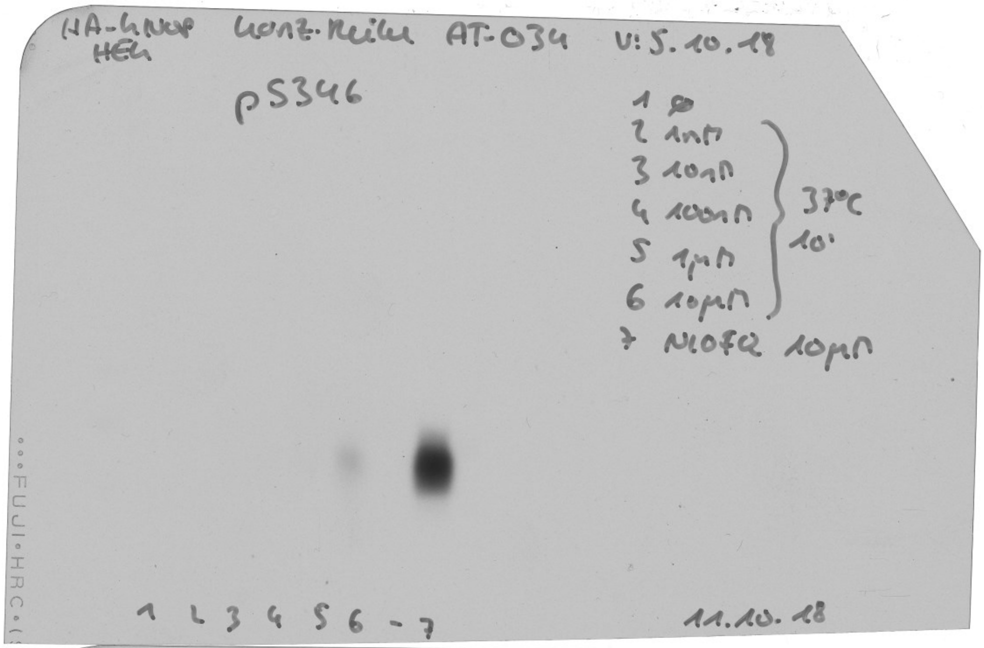
HA-hNOP HEK
Konz.Reihe
AT-034
V: 5.10.18
pS346
1 ø
2 1nM
3 10nM
4 100nM
5 1µM
6 10µM
37°C 10'
7 N/OFQ 10µM
1 2 3 4 5 6 - 7
11.10.18

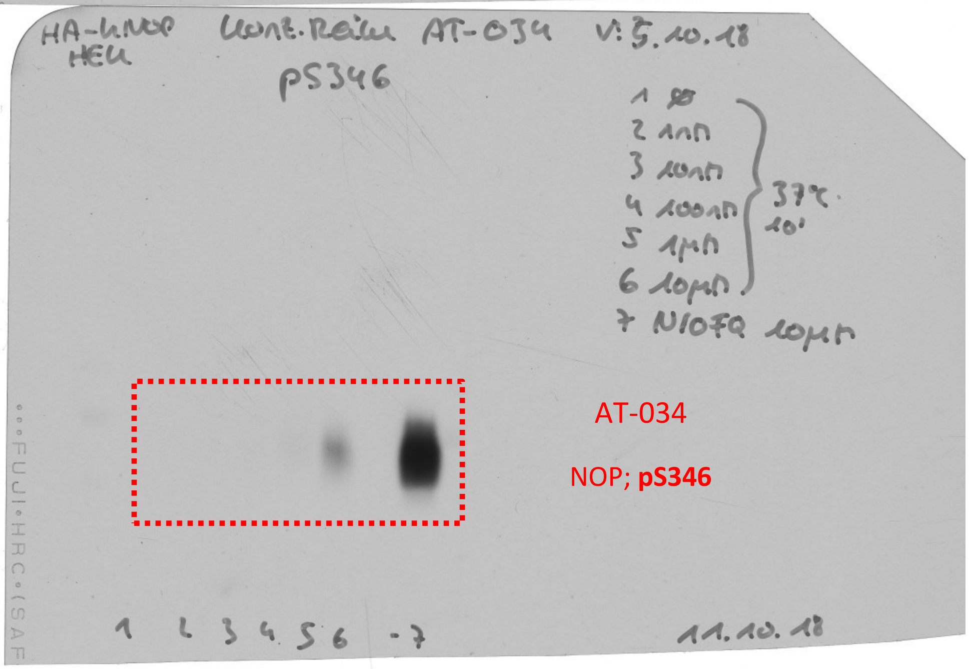
HA-hNOP HEK
Konz.Reihe
AT-034
V: 5.10.18
pS346
1 ø
2 1nM
3 10nM
4 100nM
5 1µM
6 10µM
37°C 10'
7 N/OFQ 10µM
AT-034
NOP; **pS346**
1 2 3 4 5 6 - 7
11.10.18

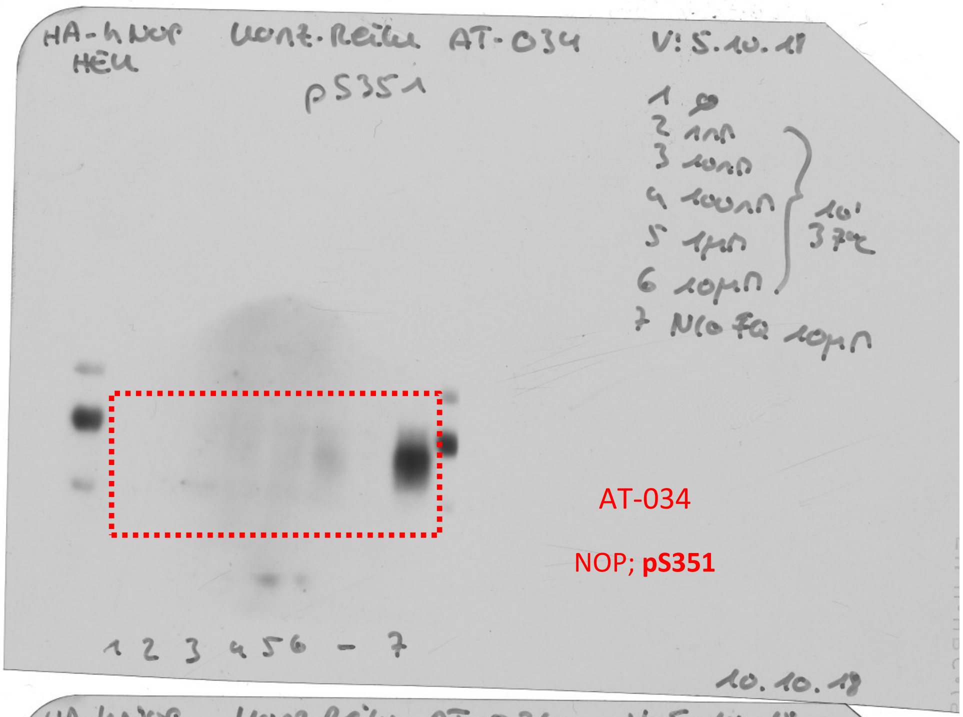
HA-hNOP
HEK
Konz. Reihe AT-034
V: 5.10.18
pS351
1 ø
2 1nM
3 10nM
4 100nM
5 1µM
6 10µM
10'
37°C
7 N/OFQ 10µM
AT-034
NOP; **pS351**
1 2 3 4 5 6 - 7
10.10.18

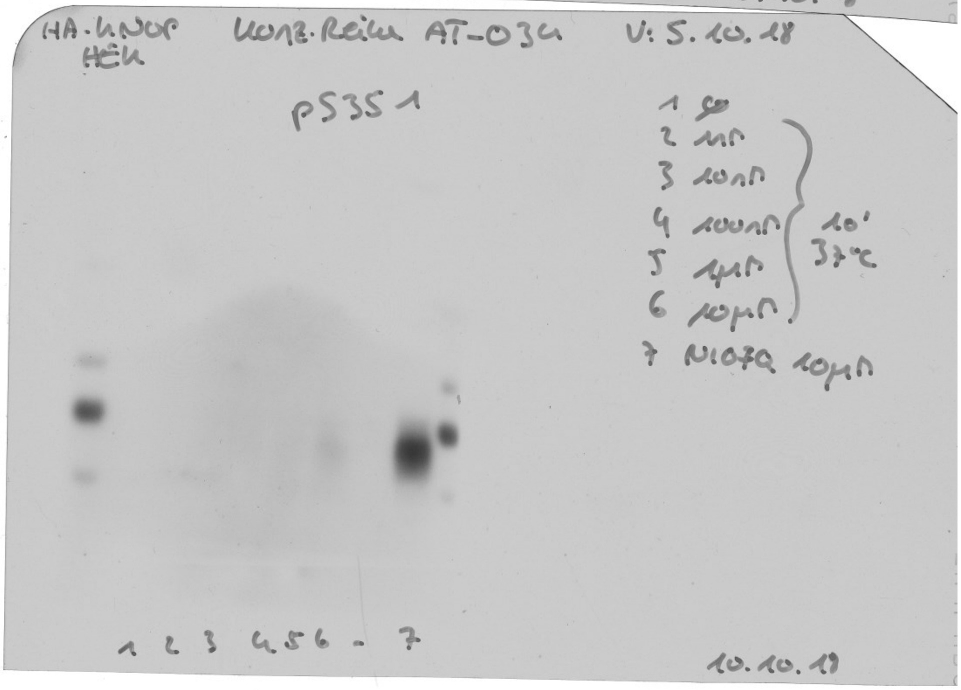
HA-hNOP
HEK
Konz. Reihe AT-034
V: 5.10.18
pS351
1 ø
2 1nM
3 10nM
4 100nM
5 1µM
6 10µM
10'
37°C
7 N/OFQ 10µM
1 2 3 4 5 6 - 7
10.10.18

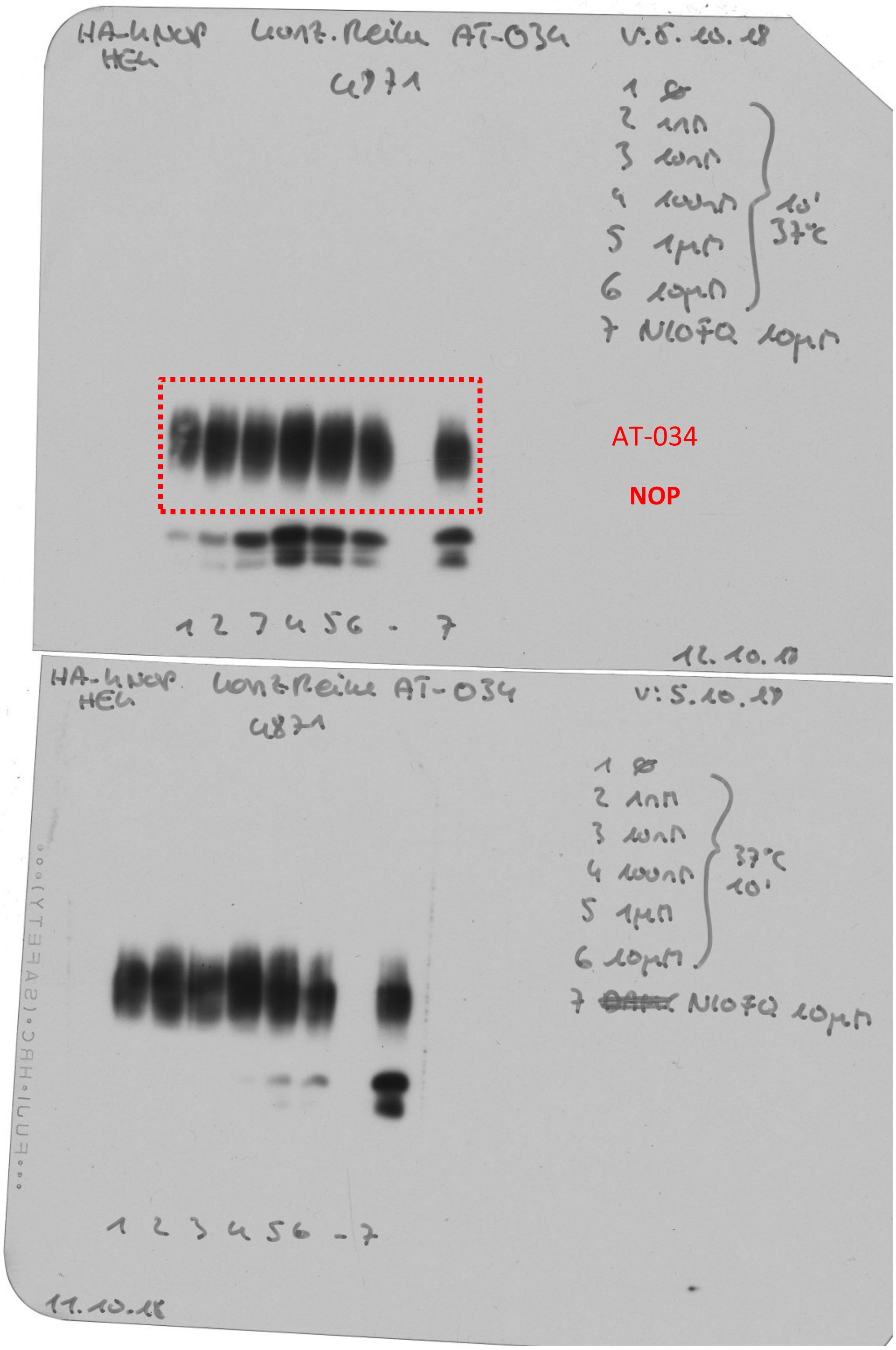
HA-hNOP HEK
AT-034
v. 5.10.18
1 Ø
2 1nM
3 10nM
4 100nM
5 1µM
6 10µM
7 N/OFQ 10µM
10' 37°C
AT-034
NOP
1 2 3 4 5 6 - 7
12.10.18
HA-hNOP HEK
AT-034
1 Ø
2 1nM
3 10nM
4 100nM
5 1µM
6 10µM
7 N/OFQ 10µM
37°C 10'
1 2 3 4 5 6 - 7
11.10.18

AT-034

<u>MOP</u>

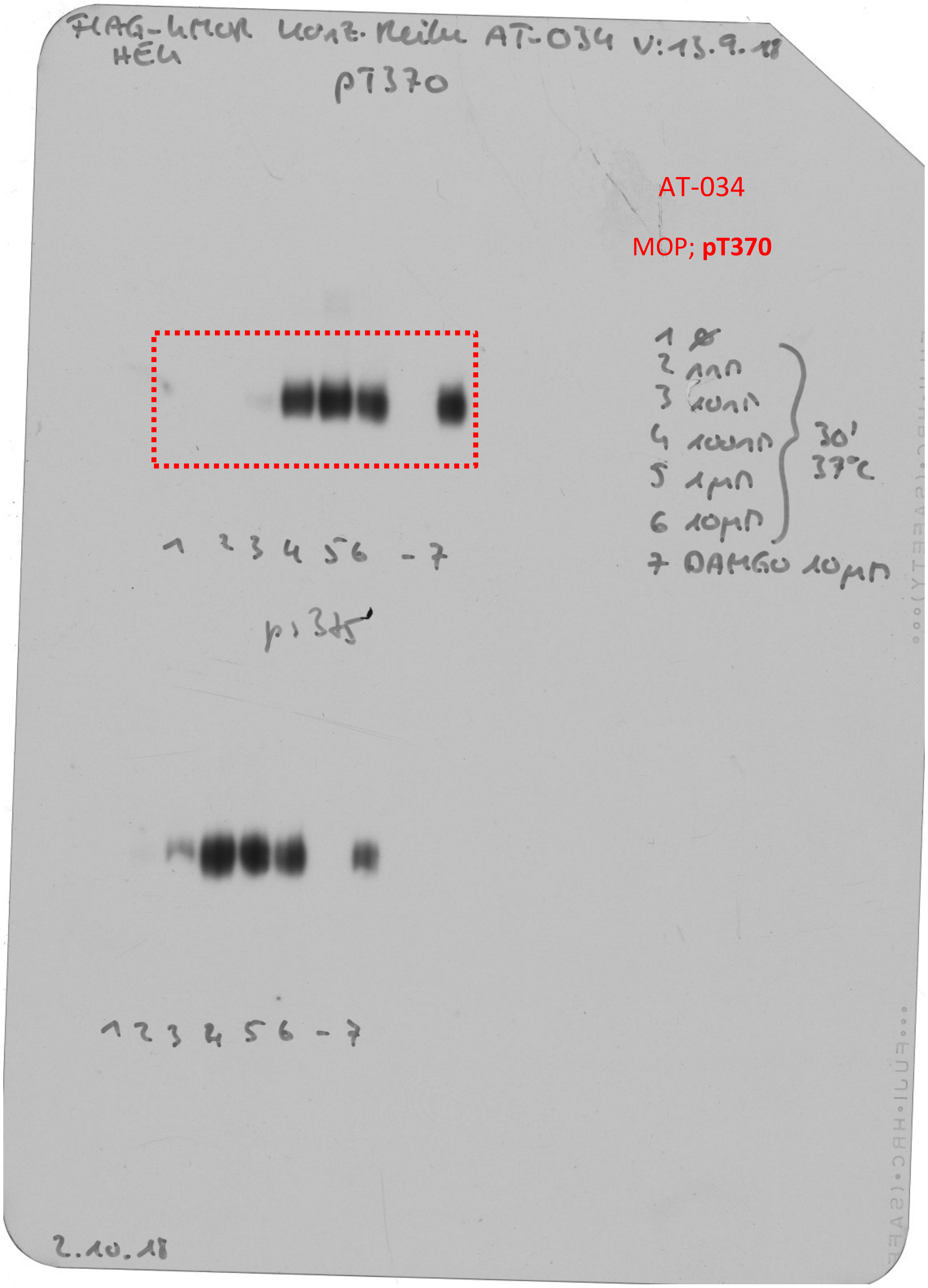
FLAG-hMOR HEK Konz.-Reihe AT-034 V: 13.9.18
pT370
AT-034
MOP; **pT370**
1 2 3 4 5 6 - 7
1 ø
2 1 nM
3 10 nM
4 100 nM
5 1 µM
6 10 µM
30' 37°C
7 DAMGO 10 µM
pT375
1 2 3 4 5 6 - 7
2.10.18

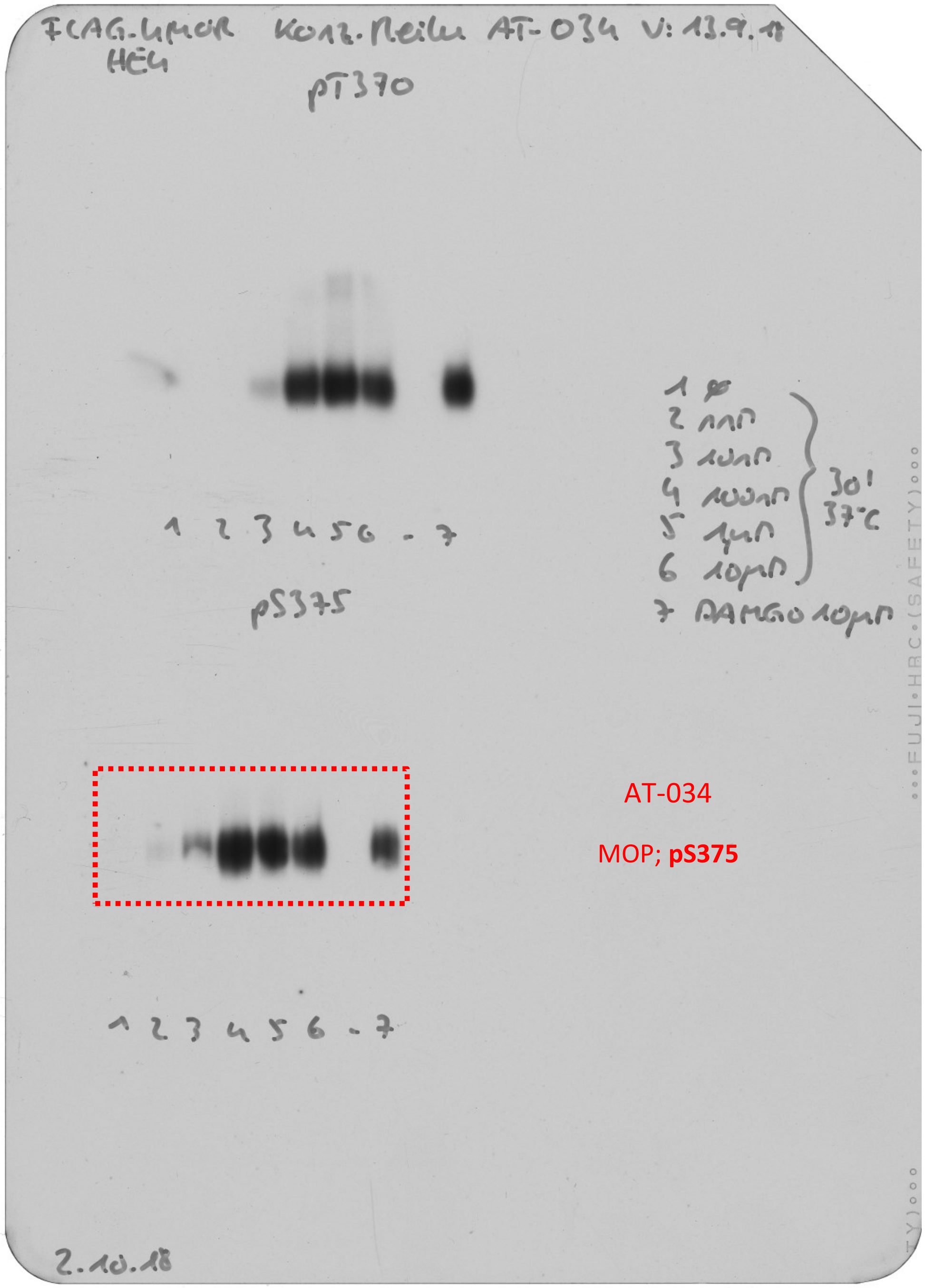
FLAG-hMOR HEK
Konz.-Reihe AT-034 V: 13.9.17
pT370
1 2 3 4 5 6 - 7
pS375
1 2 3 4 5 6 - 7
1 ø
2 1nM
3 10nM
4 100nM
5 1µM
6 10µM
30' 37°C
7 DAMGO 10µM
AT-034
MOP; **pS375**
2.10.18

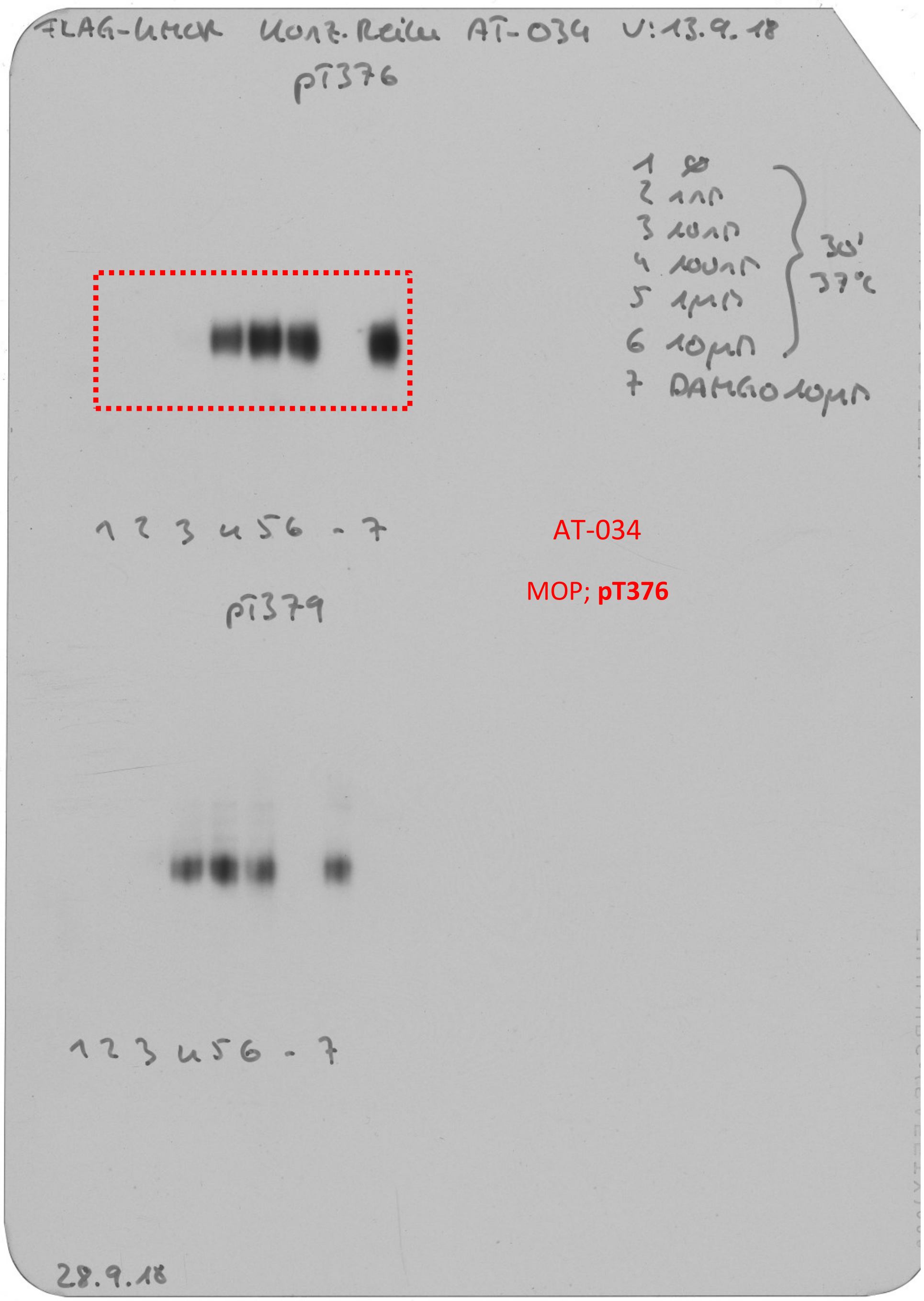

AT-034

MOP; **pT376**

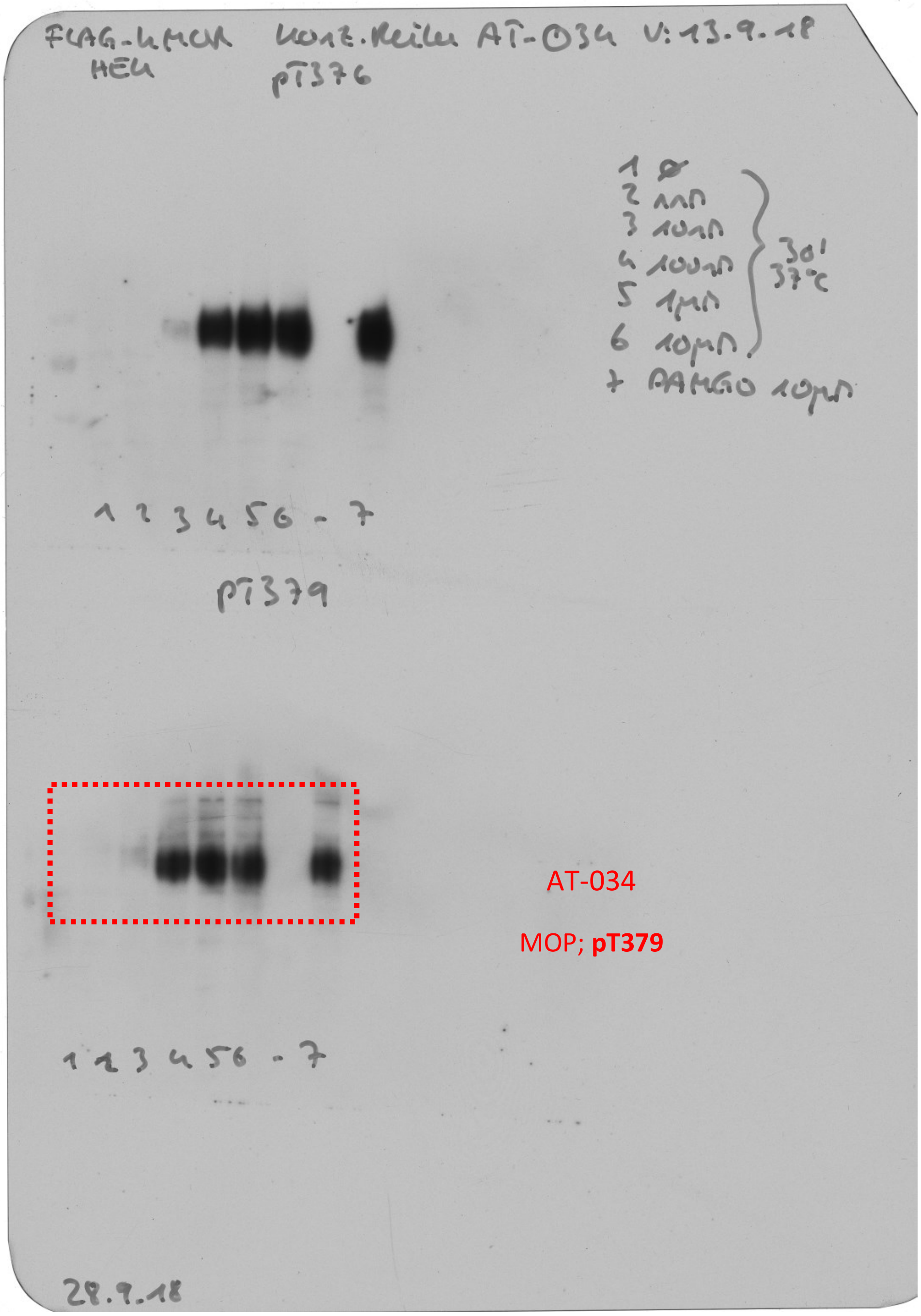

AT-034

MOP; **pT379**

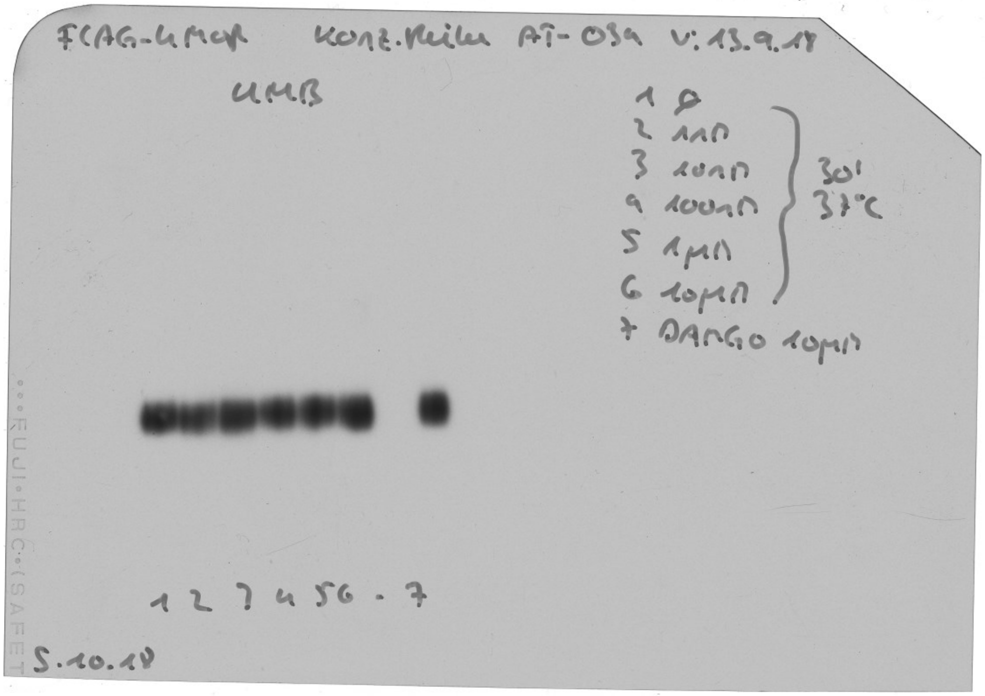
FCAG-hMOR kont.Reihe AT-034 V: 13.9.18
UMB
1 ø
2 1nM
3 10nM
4 100nM
5 1µM
6 10µM
7 DAMGO 10µM
30' 37°C
1 2 3 4 5 6 - 7
5.10.18

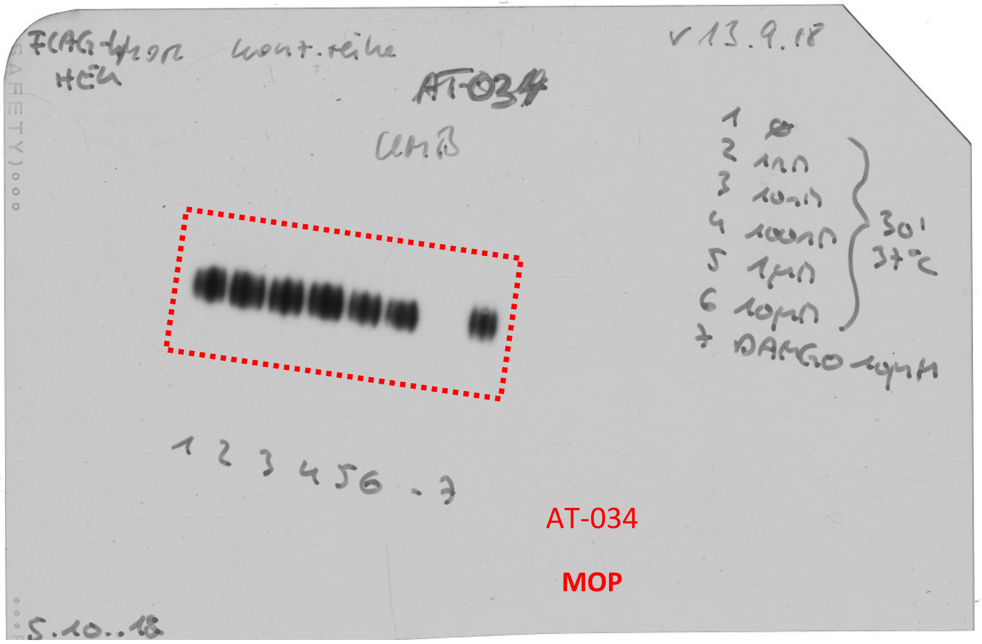
FCAG-hMOR kont.reihe
HEK
V 13.9.18
AT-034
UMB
1 ø
2 1nM
3 10nM
4 100nM
5 1µM
6 10µM
7 DAMGO 10µM
30' 37°C
1 2 3 4 5 6 - 7
5.10.18

AT-034

MOP

AT-324

<u>NOP</u>

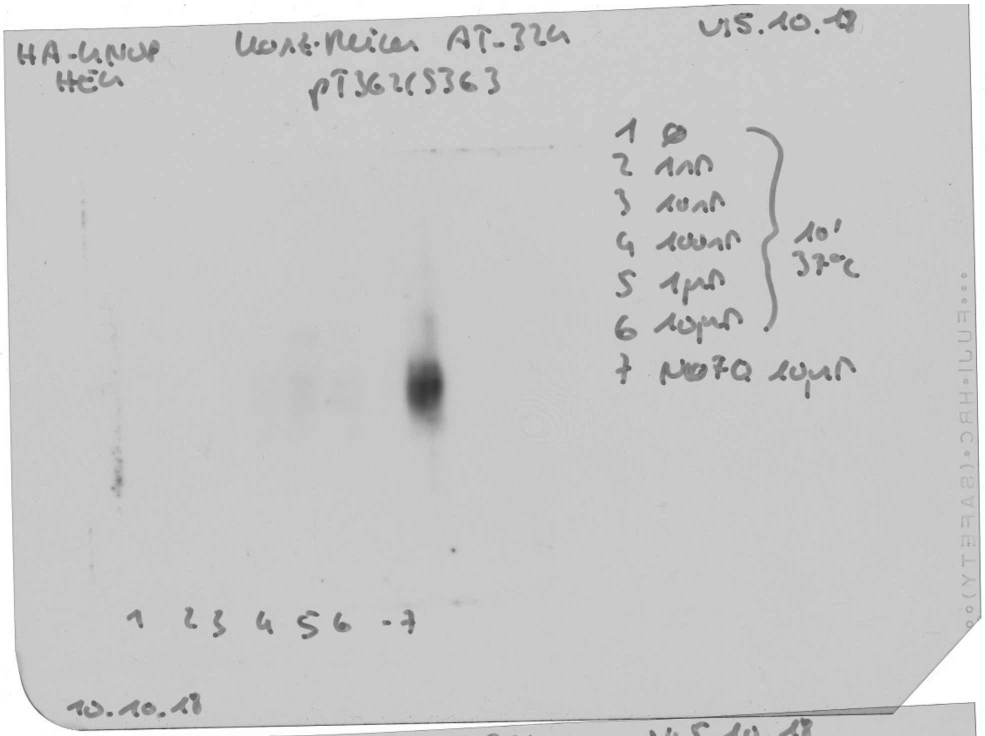

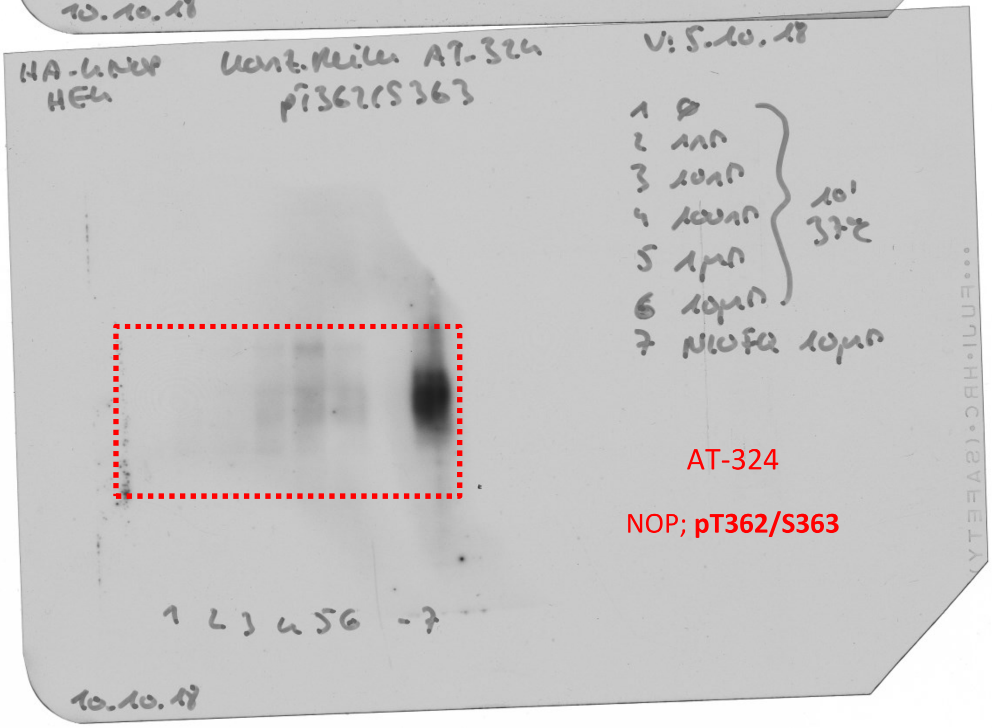
AT-324
NOP; **pT362/S363**

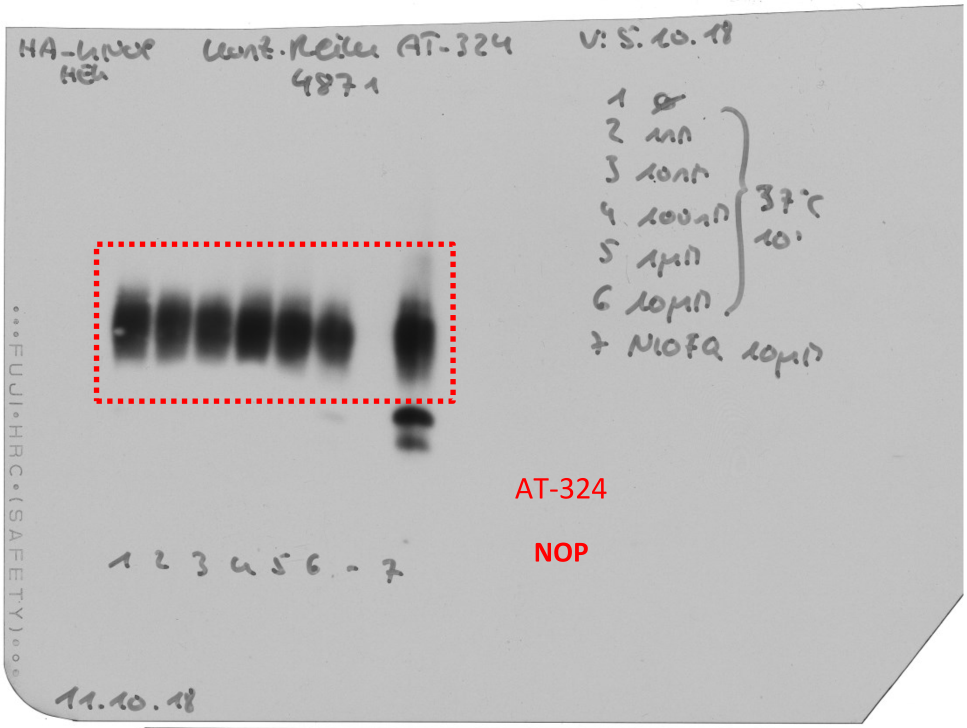
HA-hNOP
HEK
AT-324
4871
1 ø
2 1nM
3 10nM
4 100nM
5 1µM
6 10µM
37°C
10'
7 N/OFQ 10µM
AT-324
NOP
1 2 3 4 5 6 - 7
11.10.18

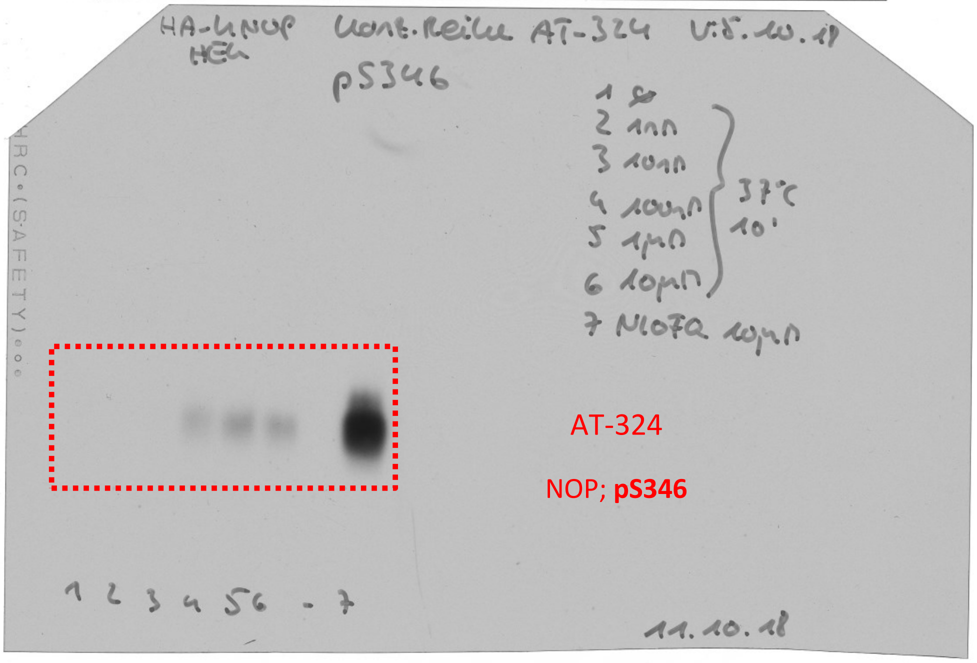
HA-hNOP
HEK
AT-324
pS346
1 ø
2 1nM
3 10nM
4 100nM
5 1µM
6 10µM
37°C
10'
7 N/OFQ 10µM
AT-324
NOP; pS346
1 2 3 4 5 6 - 7
11.10.18

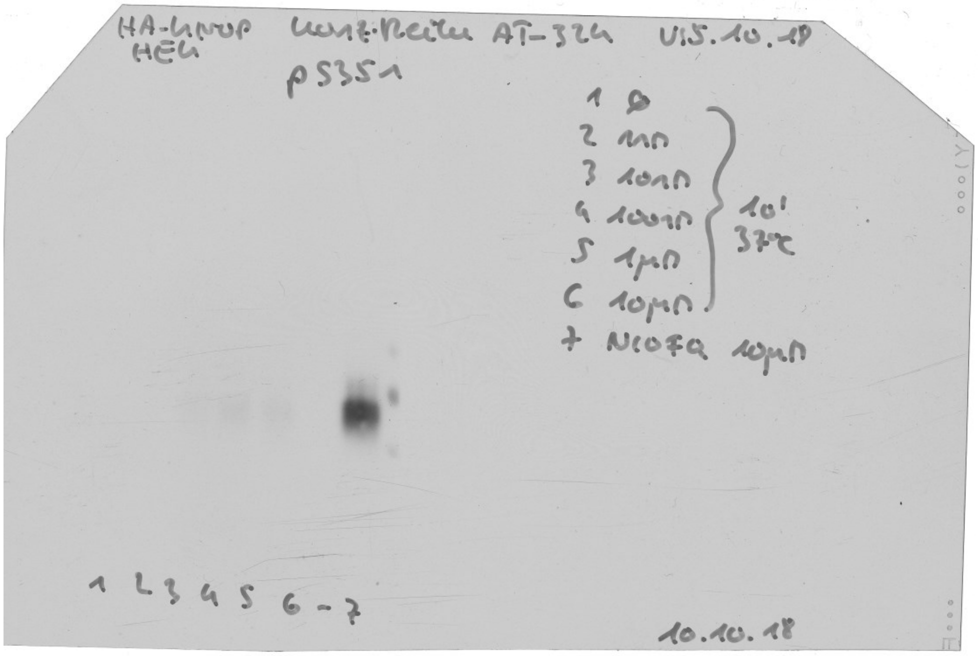
AT-324
pS351
1 2 3 4 5 6 - 7
10.10.18

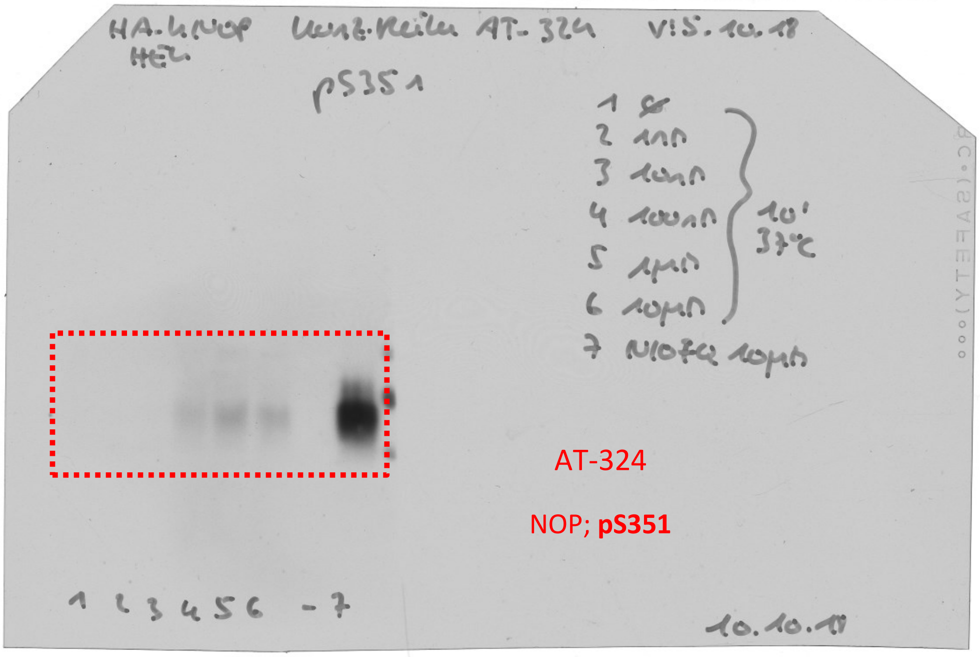
AT-324
pS351
1 2 3 4 5 6 - 7
10.10.18
AT-324
NOP; **pS351**

AT-324

<u>MOP</u>

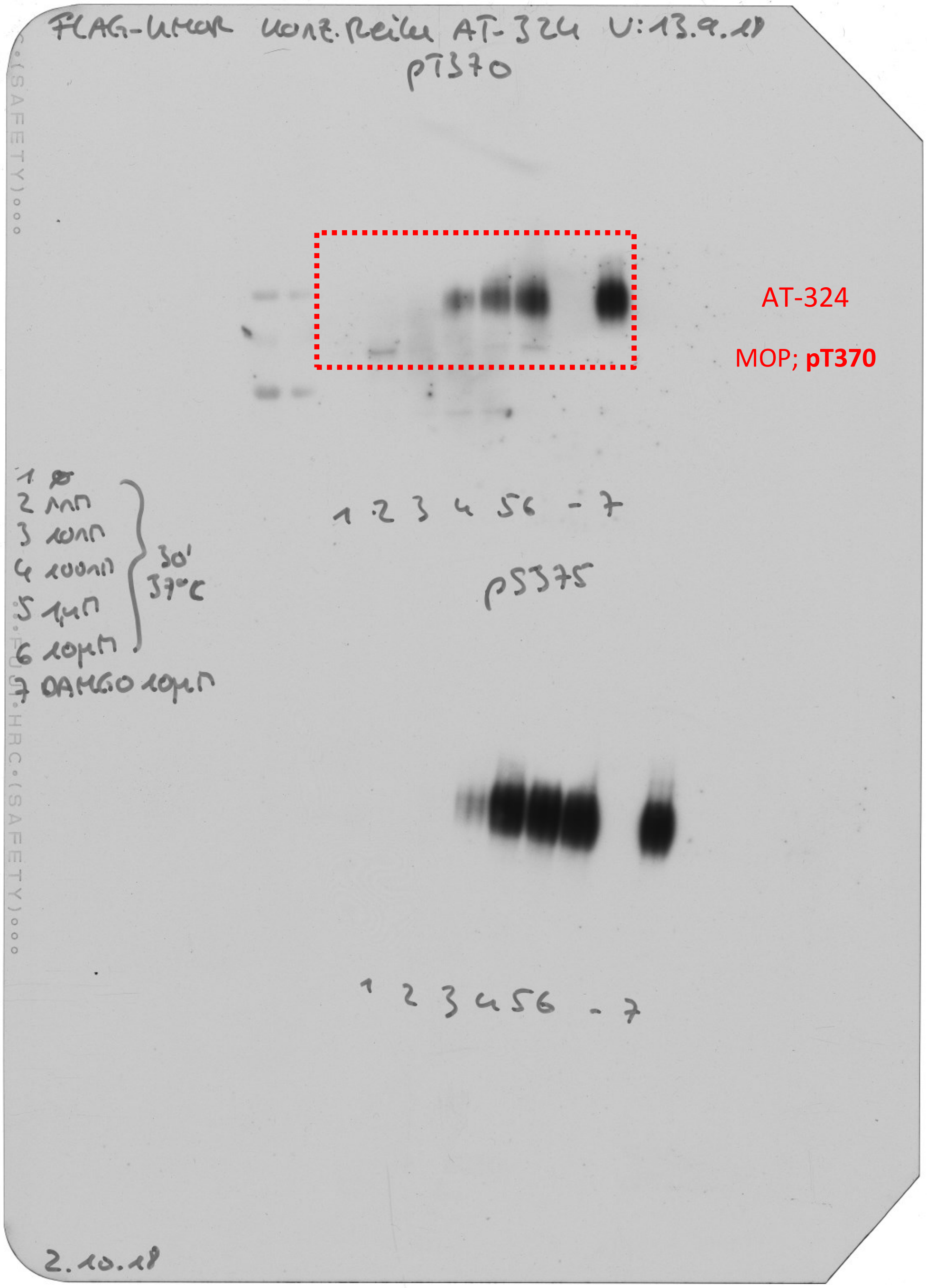

AT-324

MOP; **pT370**

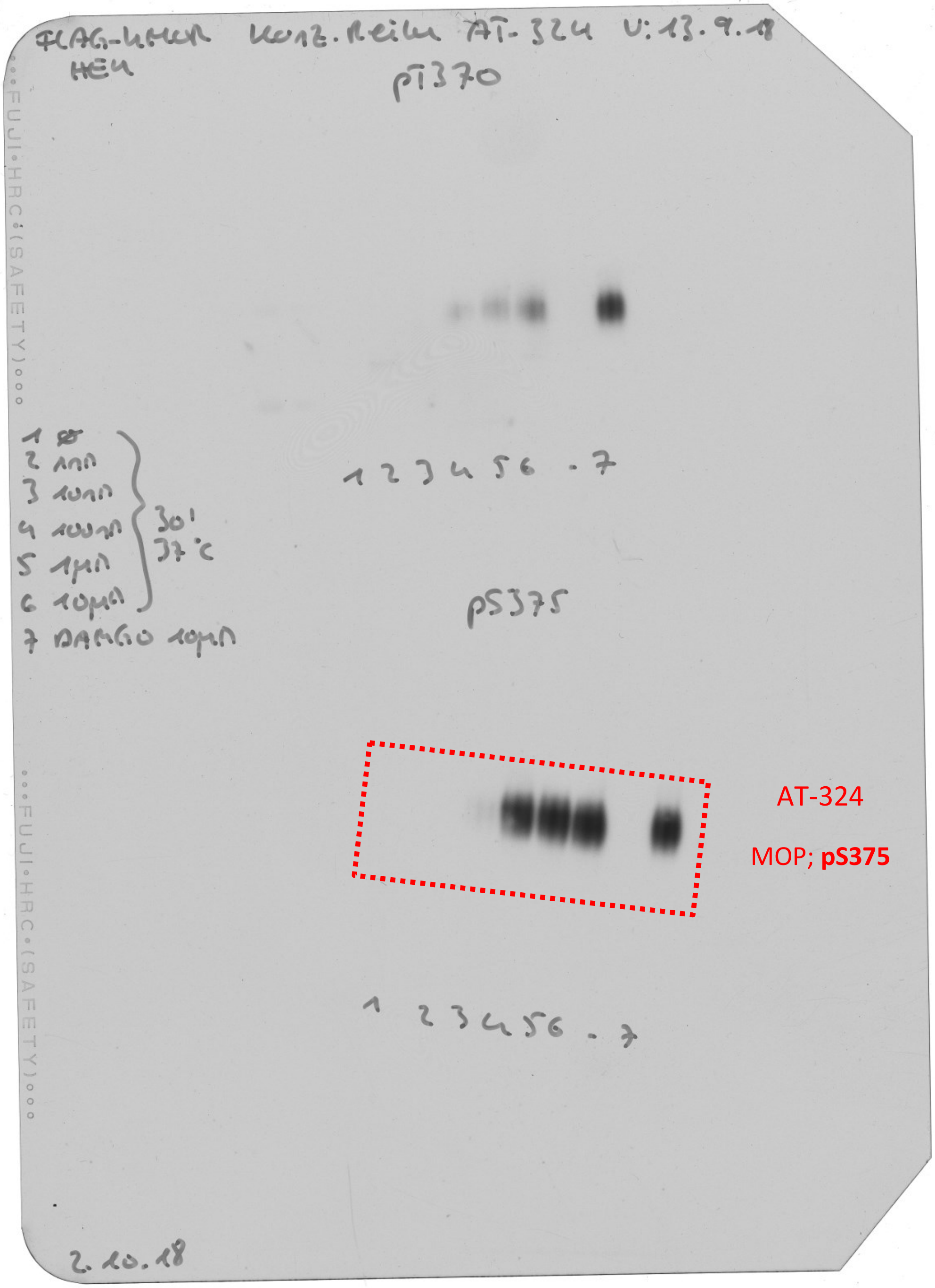
FLAG-hMOR Konz.Reihe AT-324 V: 13.9.18
HEK
pT370
1 2 3 4 5 6 . 7
1 ø
2 1nM
3 10nM
4 100nM
5 1µM
6 10µM
30' 37°C
7 DAMGO 10µM
pS375
AT-324
MOP; pS375
1 2 3 4 5 6 . 7
2.10.18

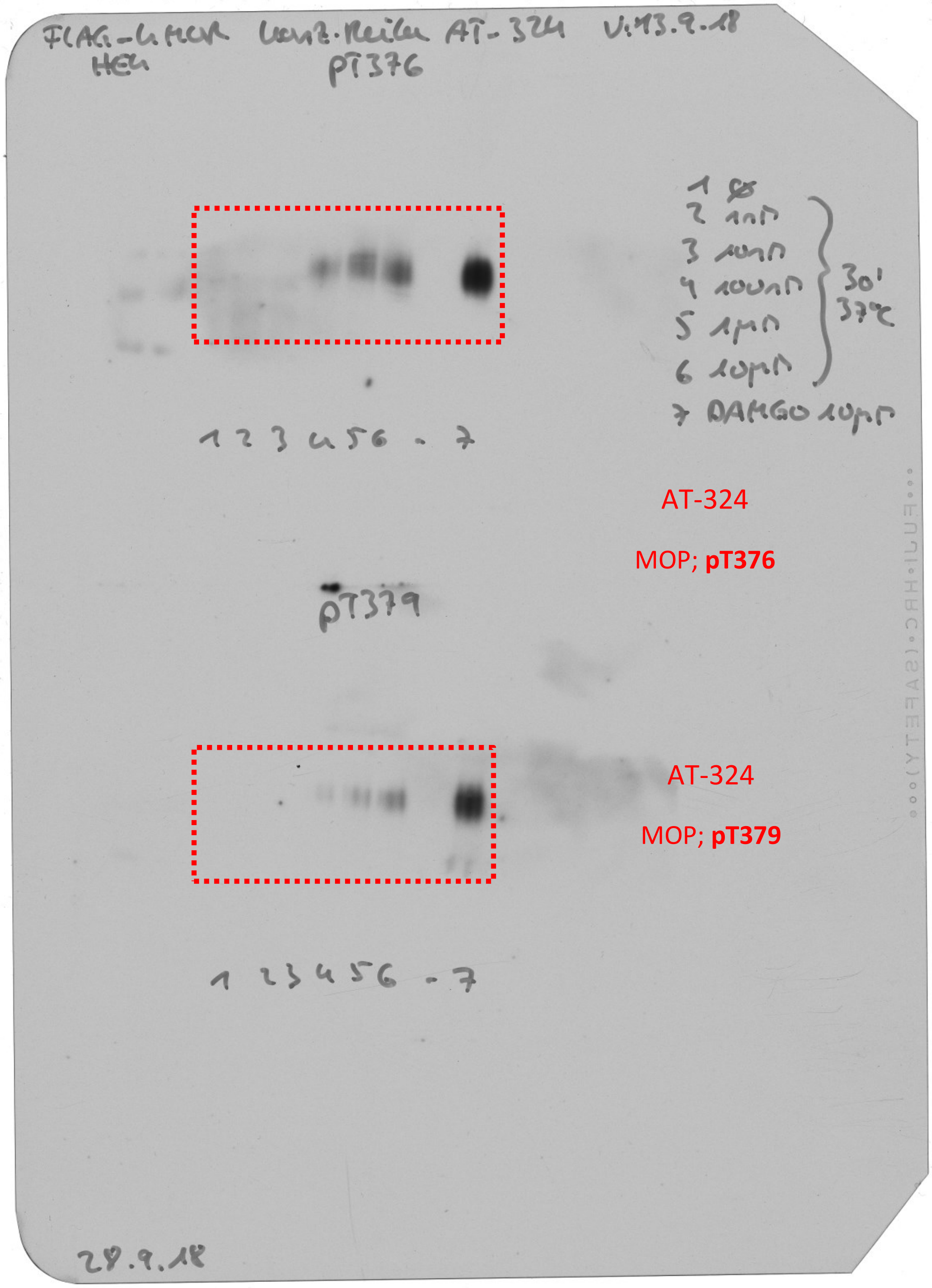
FLAG-hMOR
HEK
pT376
AT-324
1 ø
2 1nM
3 10nM
4 100nM
5 1µM
6 10µM
30'
37°C
7 DAMGO 10µM
1 2 3 4 5 6 - 7
AT-324
MOP; pT376
pT379
AT-324
MOP; pT379
1 2 3 4 5 6 - 7
28.9.18

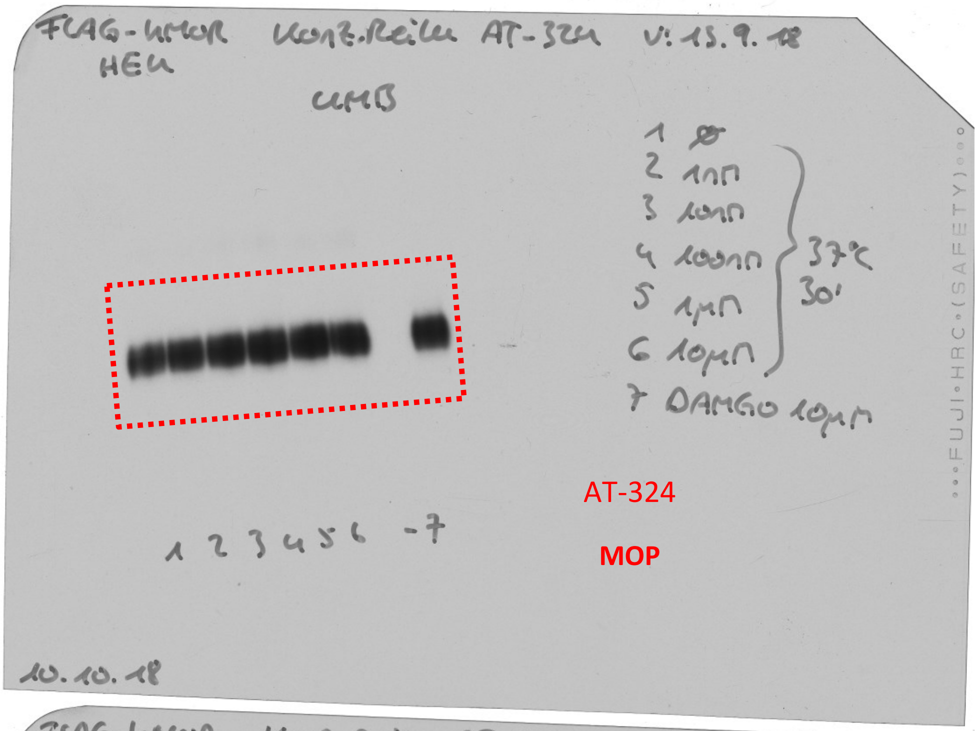
FLAG-hMOR
HEK
Konz.Reihe AT-324
V: 13.9.18
1 ∅
2 1nM
3 10nM
4 100nM
5 1µM
6 10µM
37°C
30'
7 DAMGO 10µM
1 2 3 4 5 6 -7
AT-324
MOP
FUJI•HRC•(SAFETY)
10.10.18

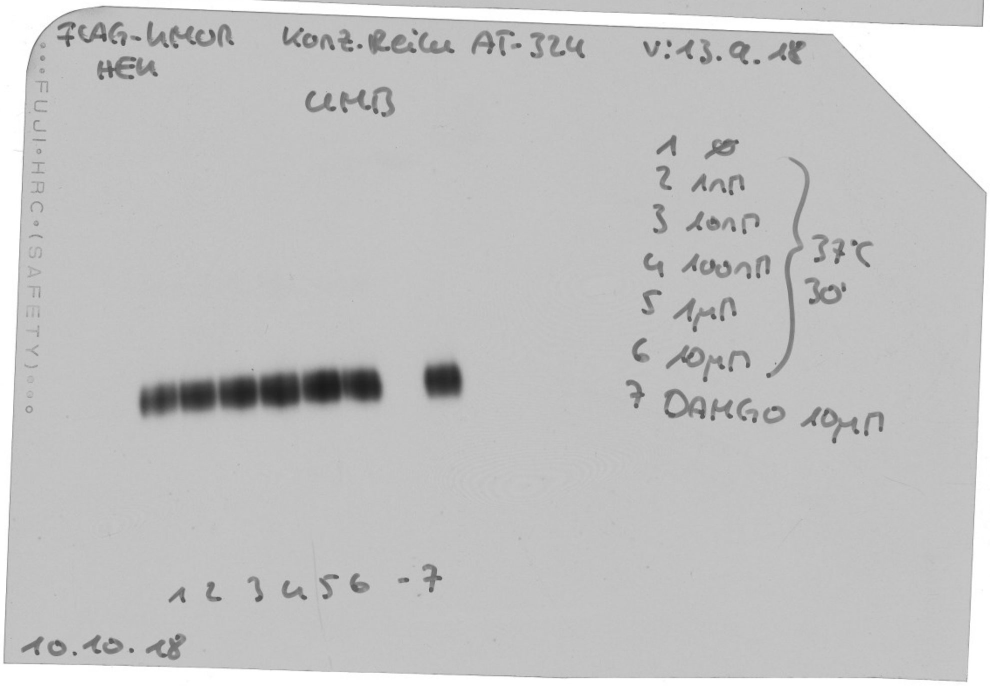
FLAG-hMOR
HEK
Konz.Reihe AT-324
V: 13.9.18
1 ∅
2 1nM
3 10nM
4 100nM
5 1µM
6 10µM
37°C
30'
7 DAMGO 10µM
1 2 3 4 5 6 -7
FUJI•HRC•(SAFETY)
10.10.18

AT-201

NOP

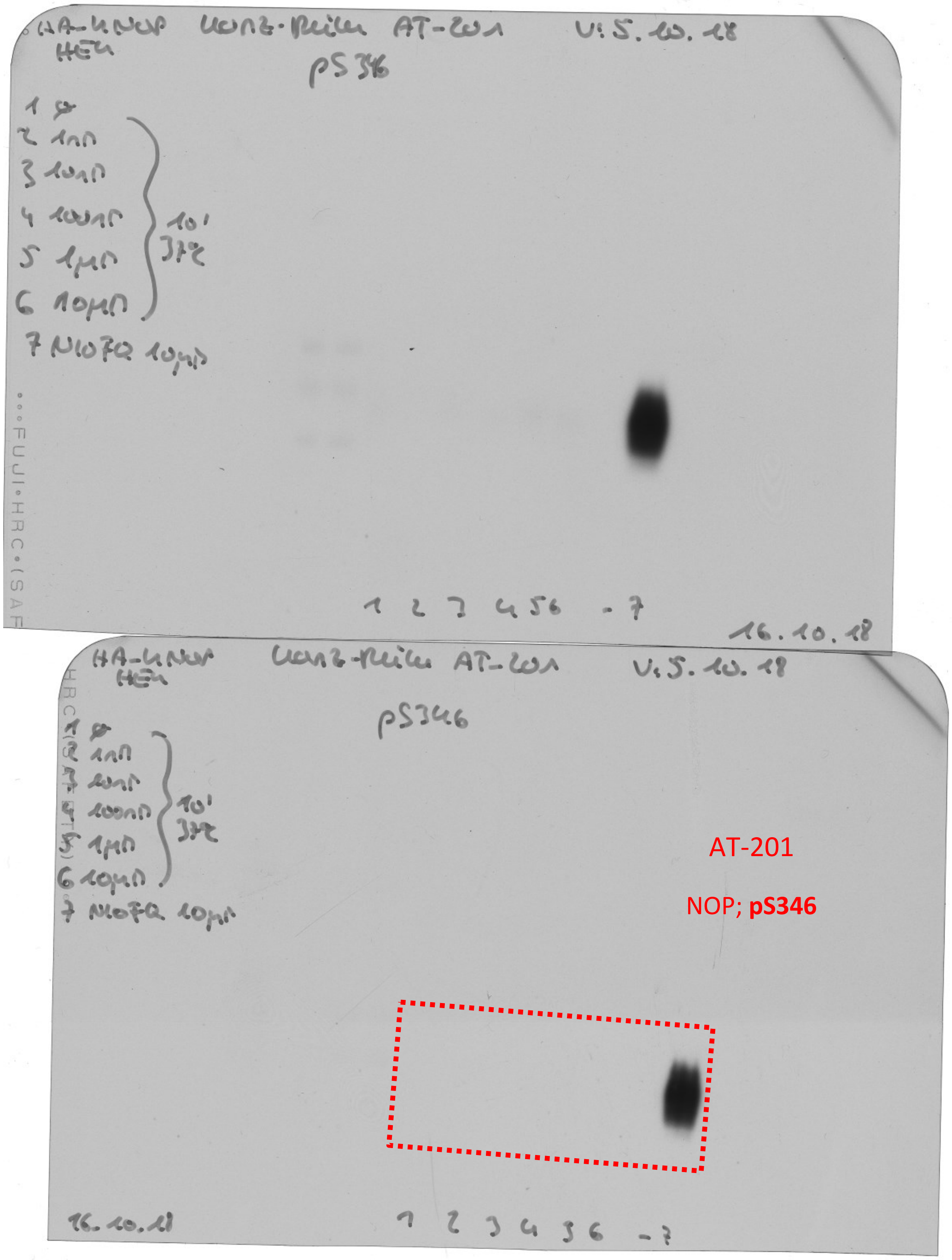
AT-201
NOP; **pS346**
pS346
16.10.18

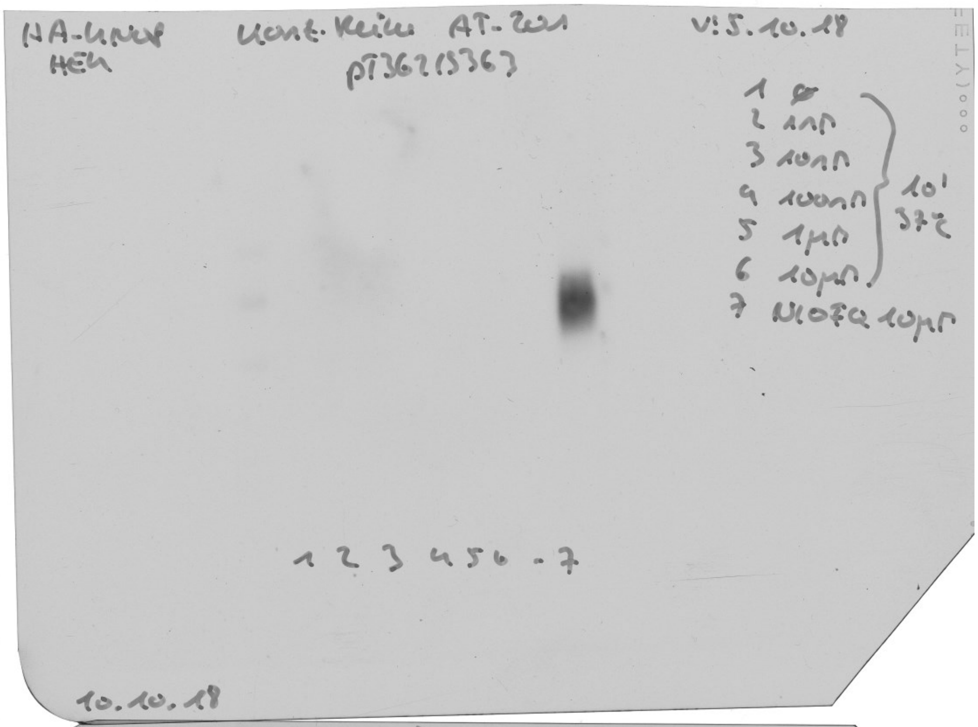
HA-hNOP
HEK
Kont. Reihe AT-201
pT362/S363
V: 5.10.18
1 Ø
2 1nM
3 10nM
4 100nM
5 1µM
6 10µM
10'
37°C
7 N/OFQ 10µM
1 2 3 4 5 6 - 7
10.10.18

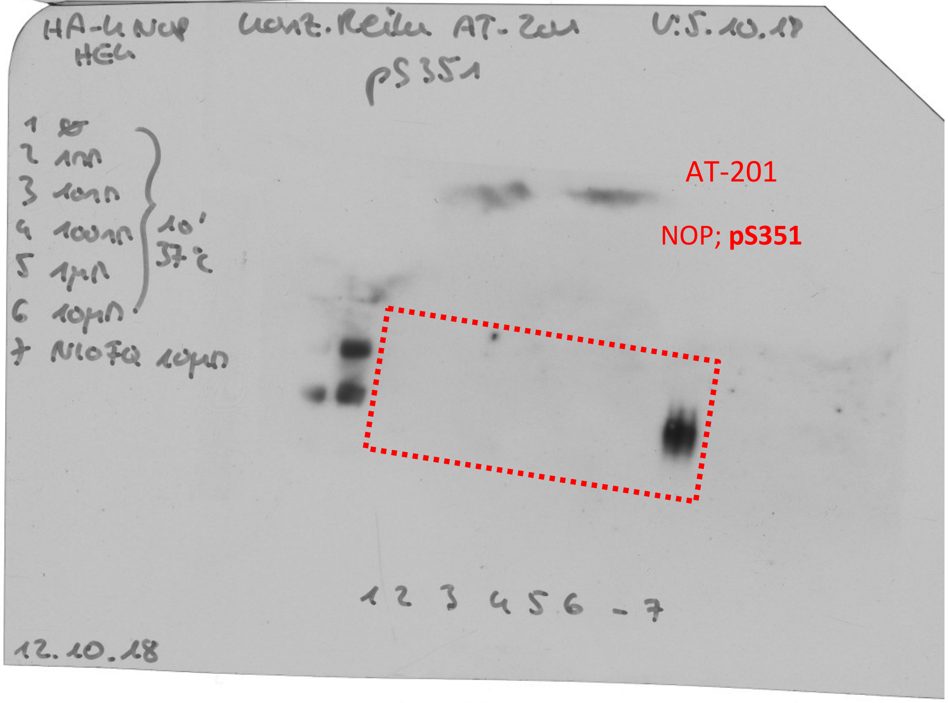
HA-hNOP
HEK
Kont. Reihe AT-201
V: 5.10.18
pS351
1 Ø
2 1nM
3 10nM
4 100nM
5 1µM
6 10µM
10'
37°C
7 N/OFQ 10µM
AT-201
NOP; pS351
1 2 3 4 5 6 - 7
12.10.18

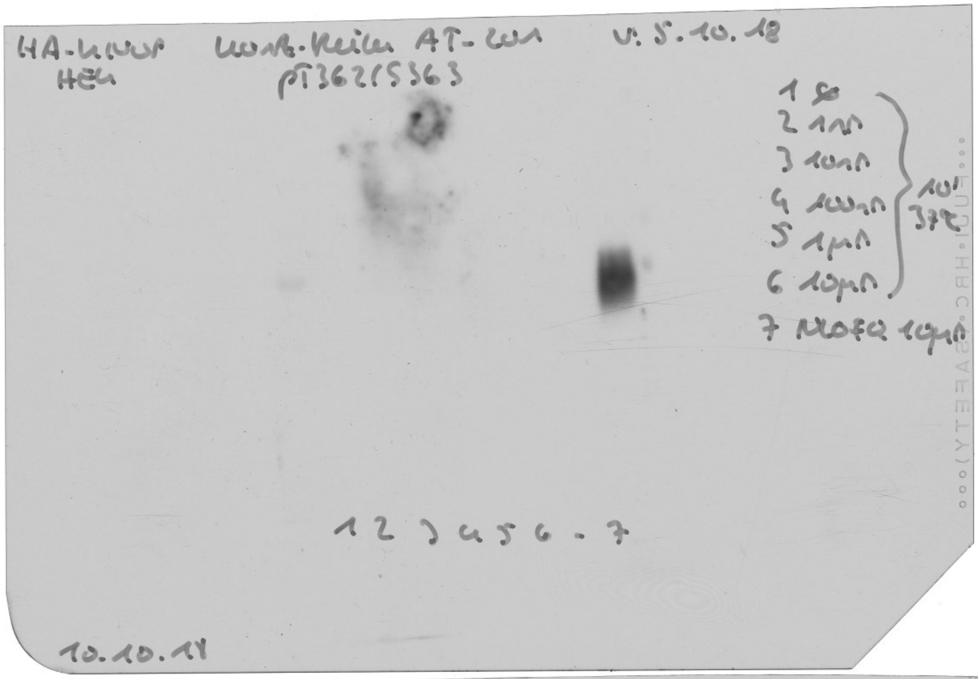
HA-hNOP
HEK
pT362/S363
v. 5.10.18
1 2 3 4 5 6 - 7
10.10.18

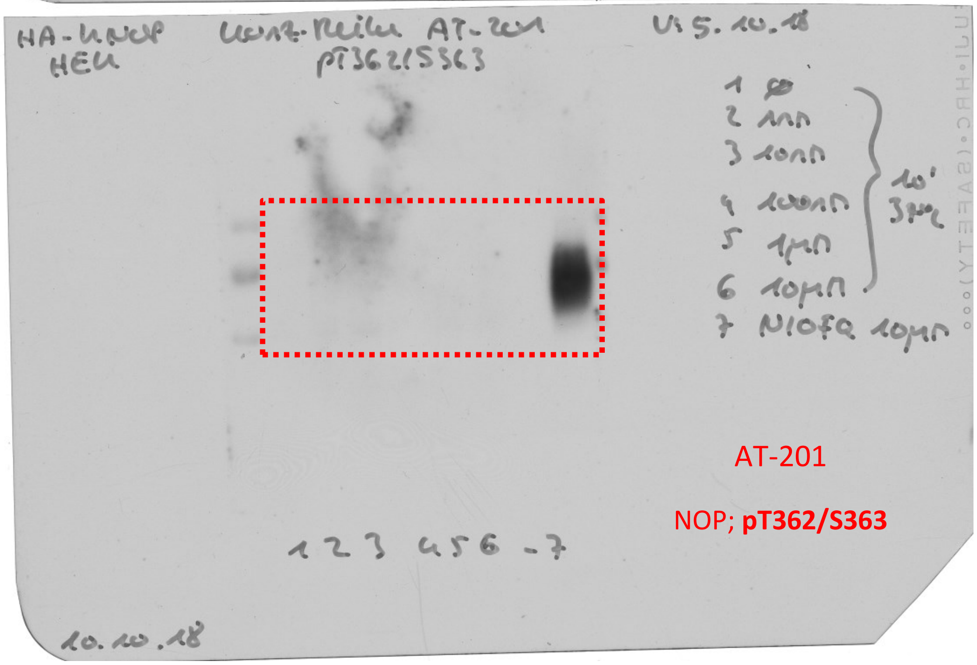
HA-hNOP
HEK
pT362/S363
1 2 3 4 5 6 - 7
10.10.18
AT-201
NOP; **pT362/S363**

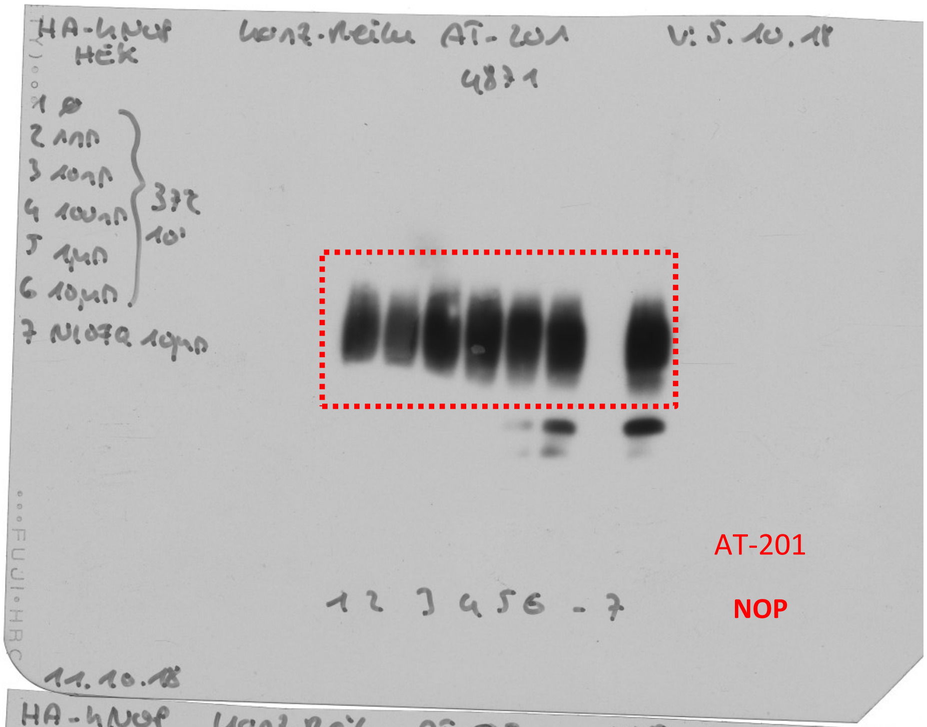
AT-201
NOP

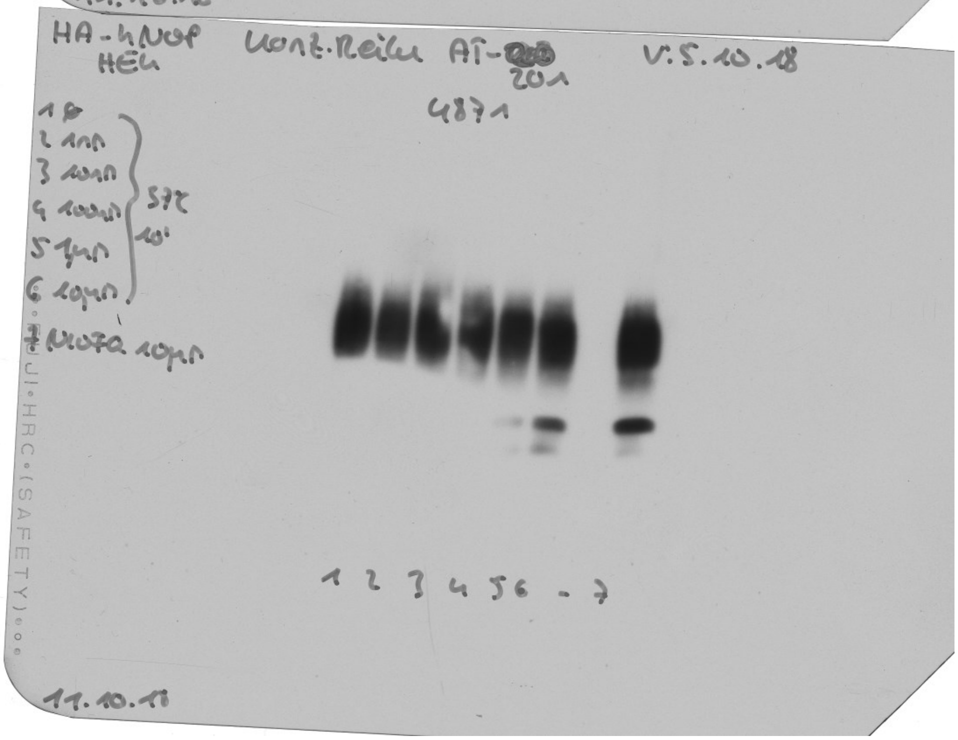

AT-201

<u>MOP</u>

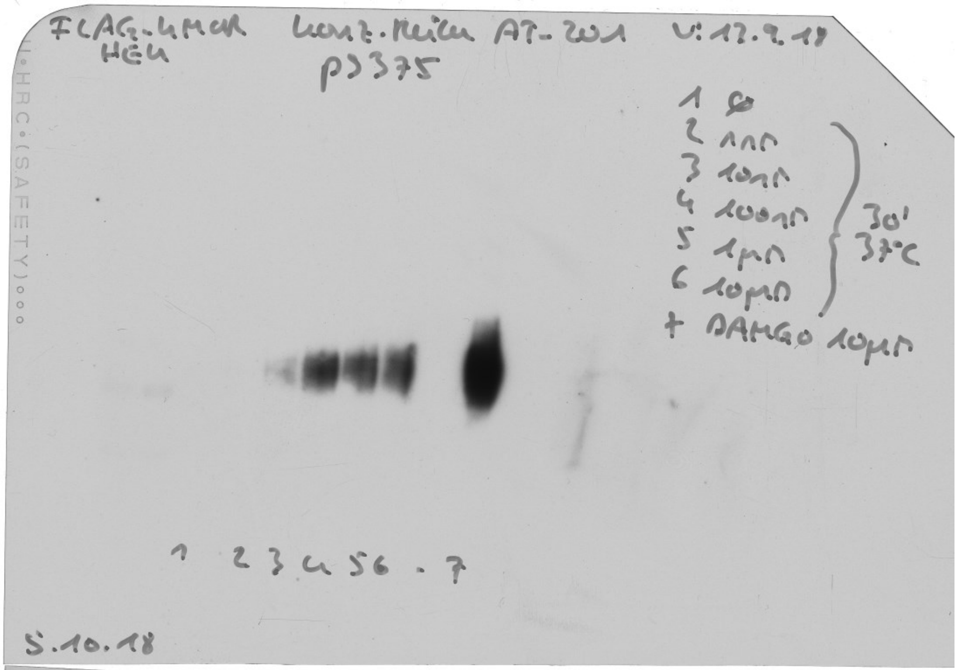

FLAG-hMOR HEK
AT-201
pS375
1 Ø
2 1nM
3 10nM
4 100nM
5 1µM
6 10µM
7 DAMGO 10µM
30'
37°C
1 2 3 4 5 6 - 7
5.10.18

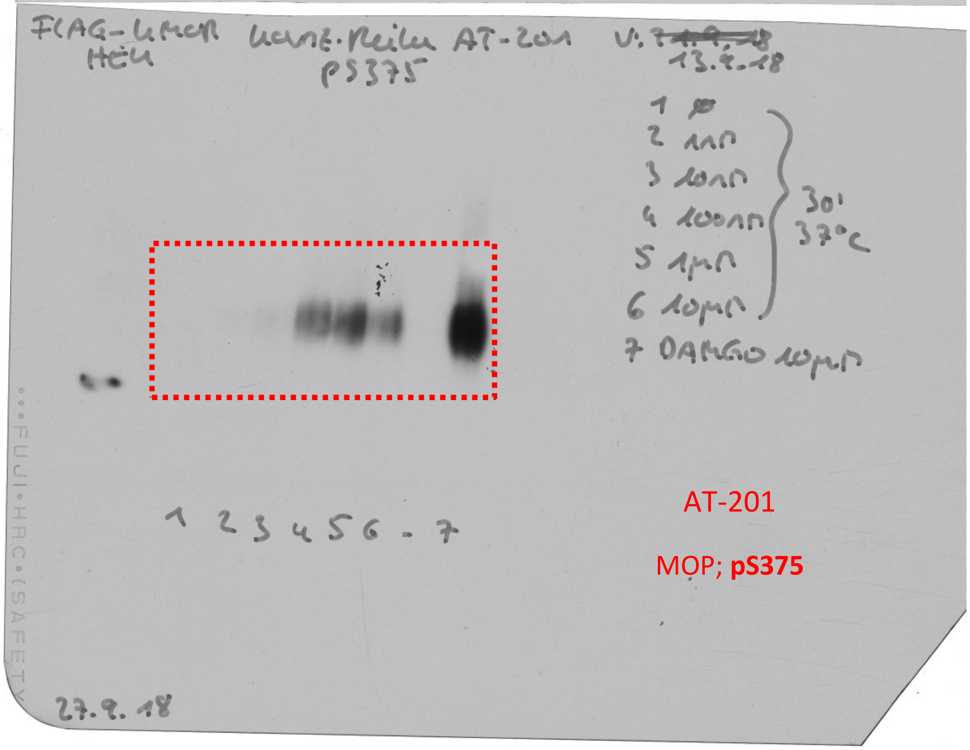

FLAG-hMOR HEK
AT-201
pS375
13.9.18
1 Ø
2 1nM
3 10nM
4 100nM
5 1µM
6 10µM
7 DAMGO 10µM
30'
37°C
1 2 3 4 5 6 - 7
27.9.18
AT-201
MOP; **pS375**

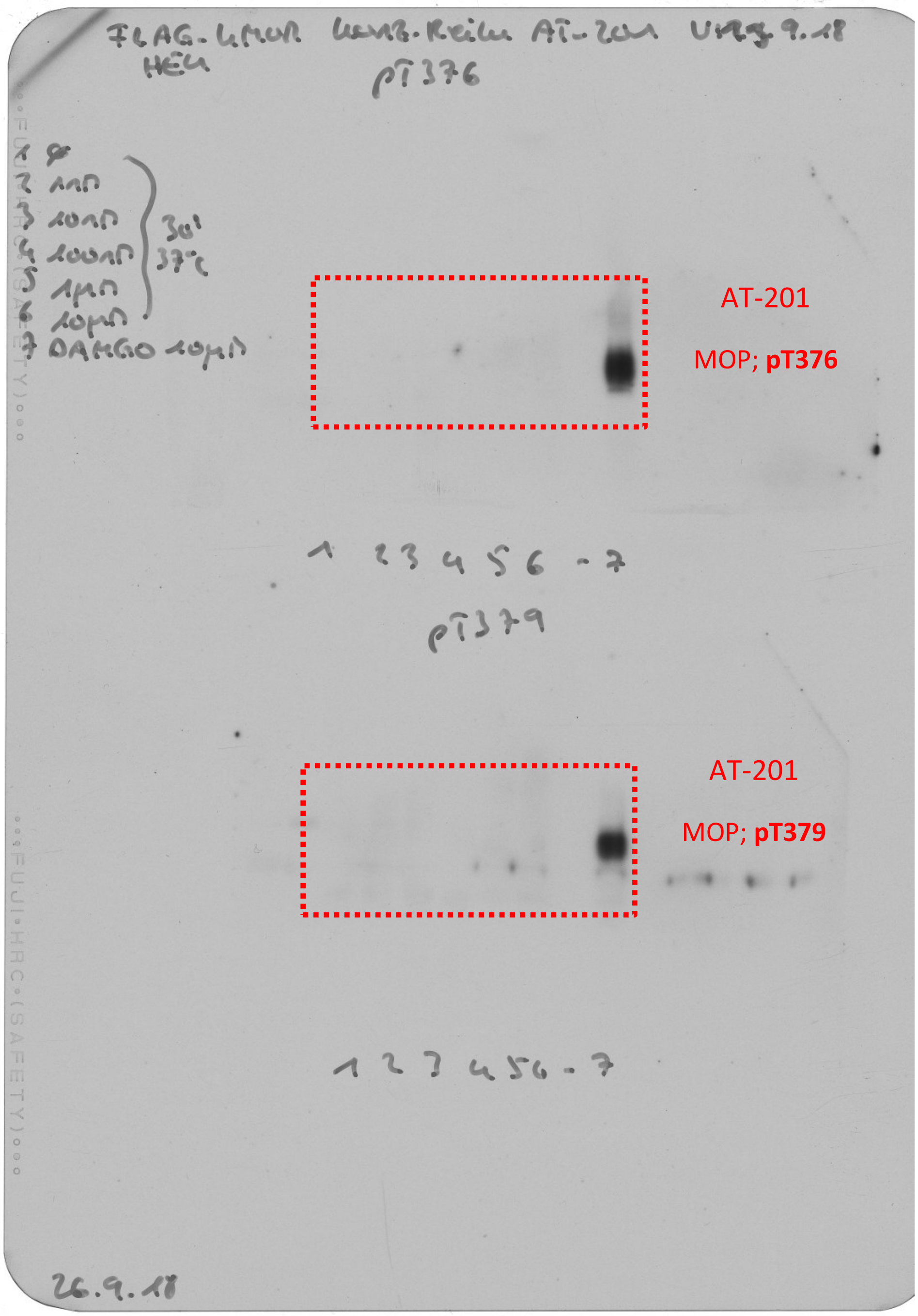

pT376
DAMGO
AT-201
MOP; pT376
pT379
AT-201
MOP; pT379
26.9.18

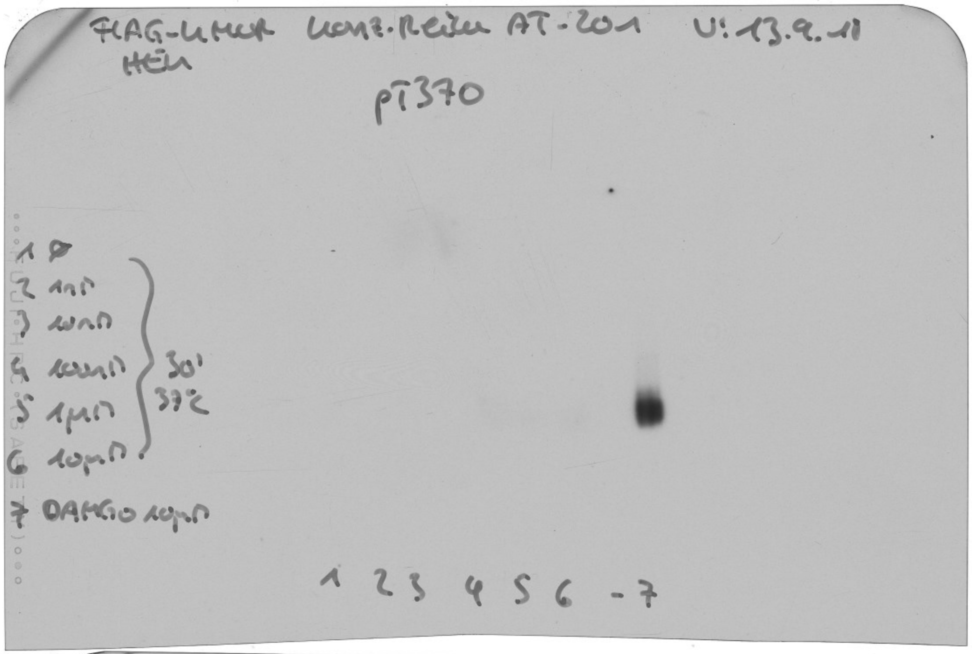

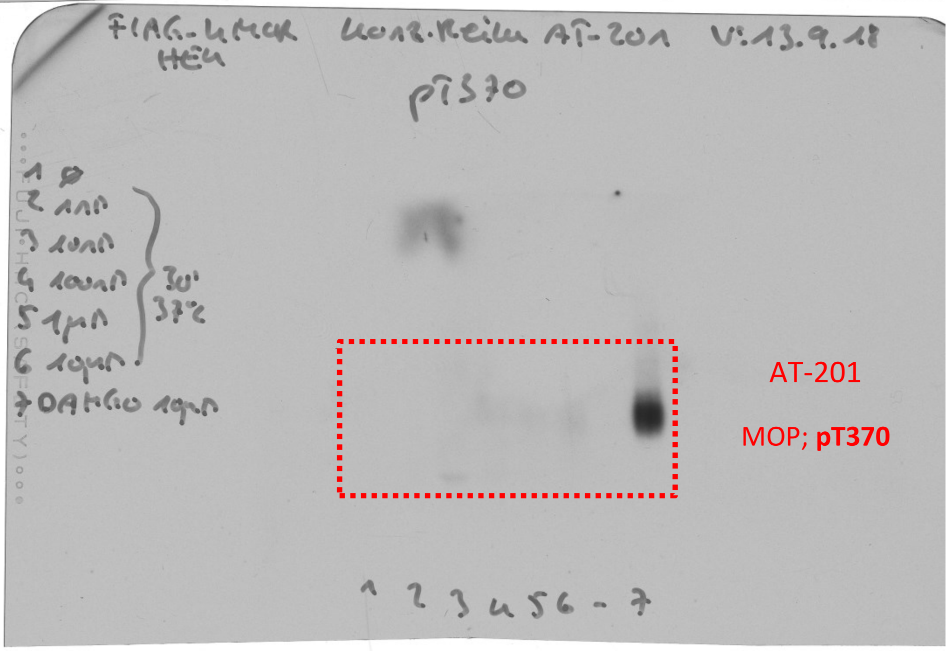

AT-201

MOP; **pT370**

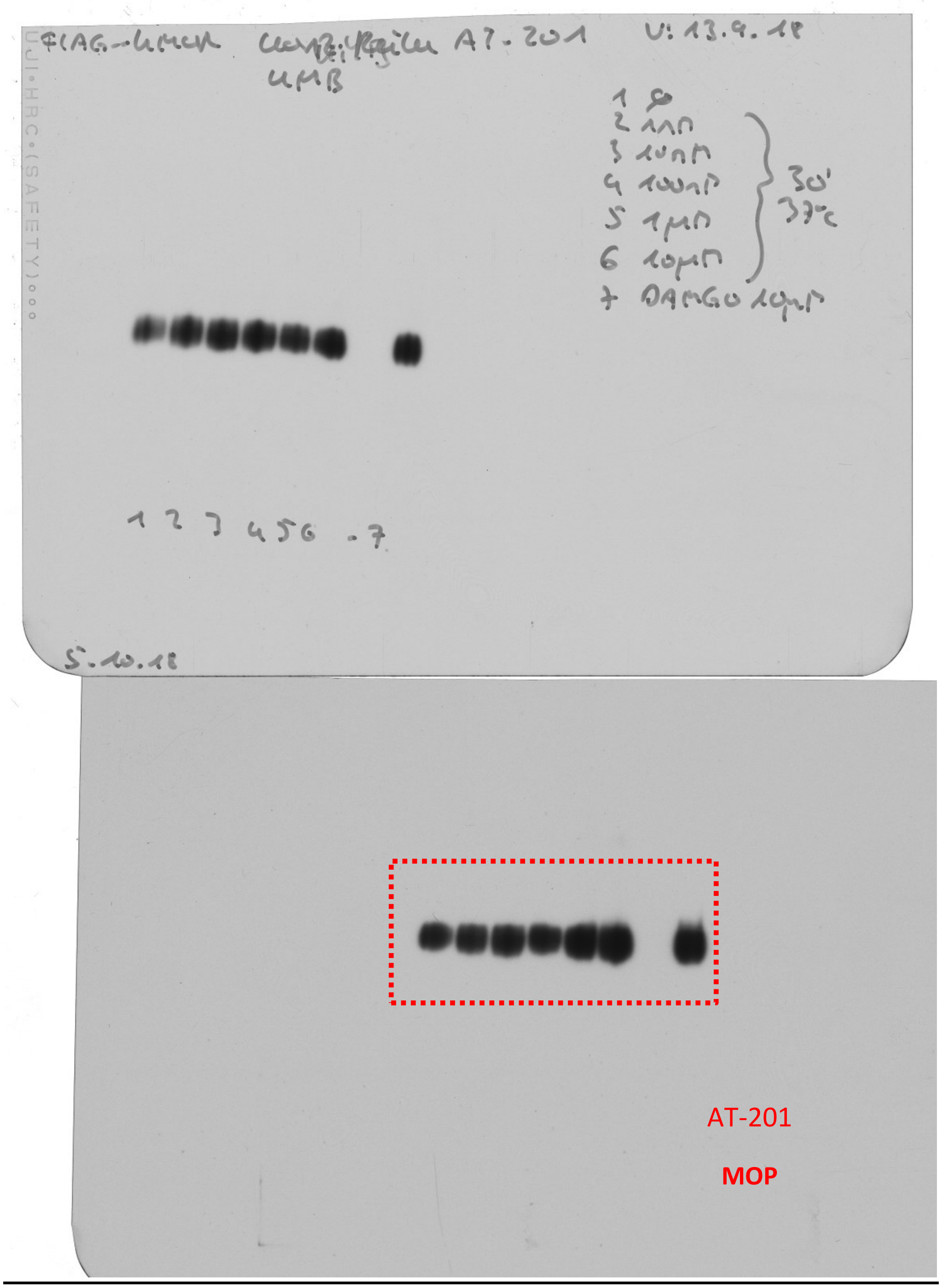
AT-201
V: 13.9.18
1 2 3 4 5 6 7
30'
37°C
DAMGO
5.10.18
AT-201
MOP

AT-121

NOP

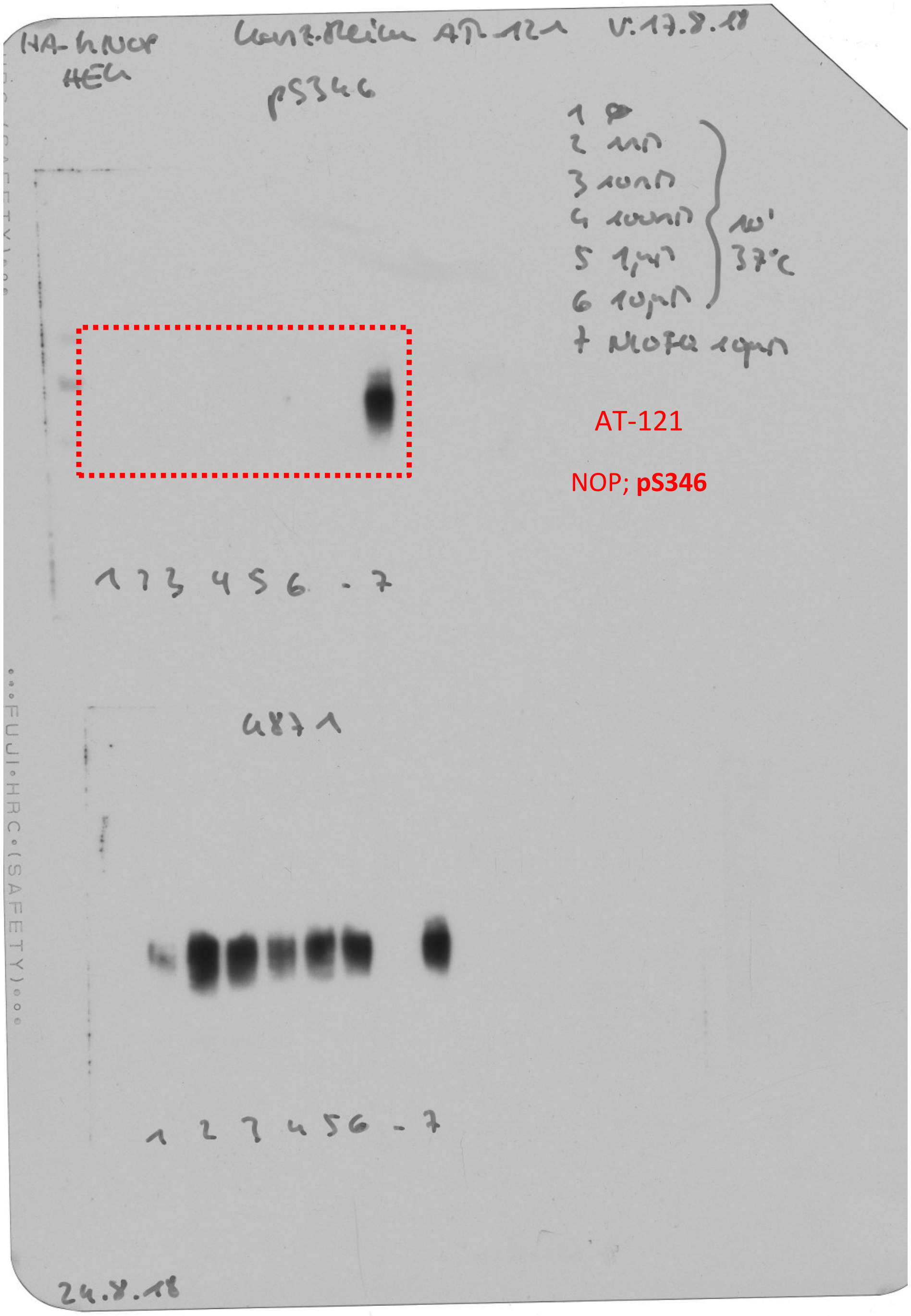
HA-hNOP
HEK
pS346
AT-121
NOP; pS346
1 2 3 4 5 6 - 7
1 2 3 4 5 6 - 7
24.8.18

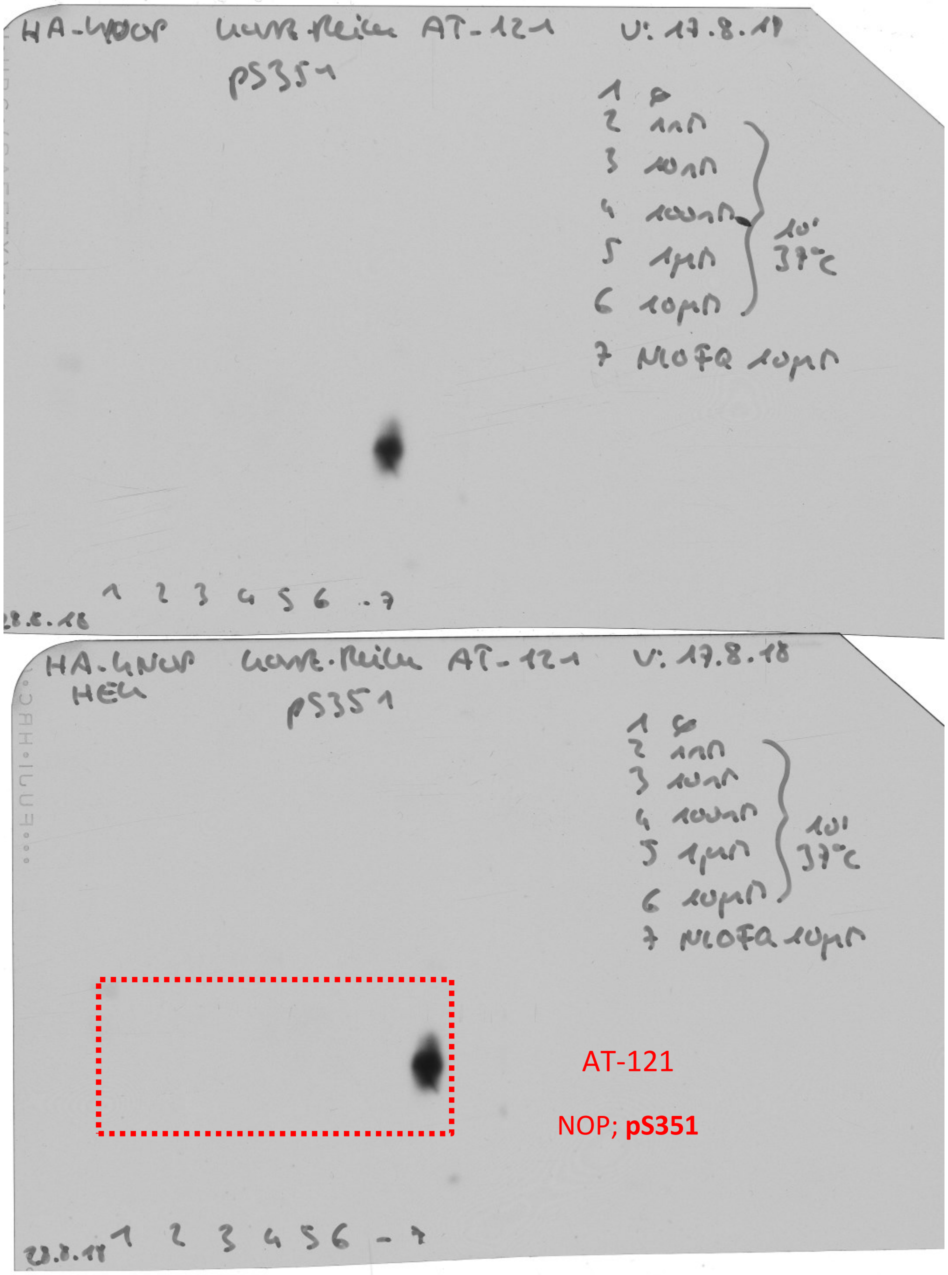
HA-
AT-121
pS351
1 ø
2 1nM
3 10nM
4 100nM
5 1µM
6 10µM
10'
37°C
7 N/OFQ 10µM
1 2 3 4 5 6 - 7
AT-121
NOP; **pS351**

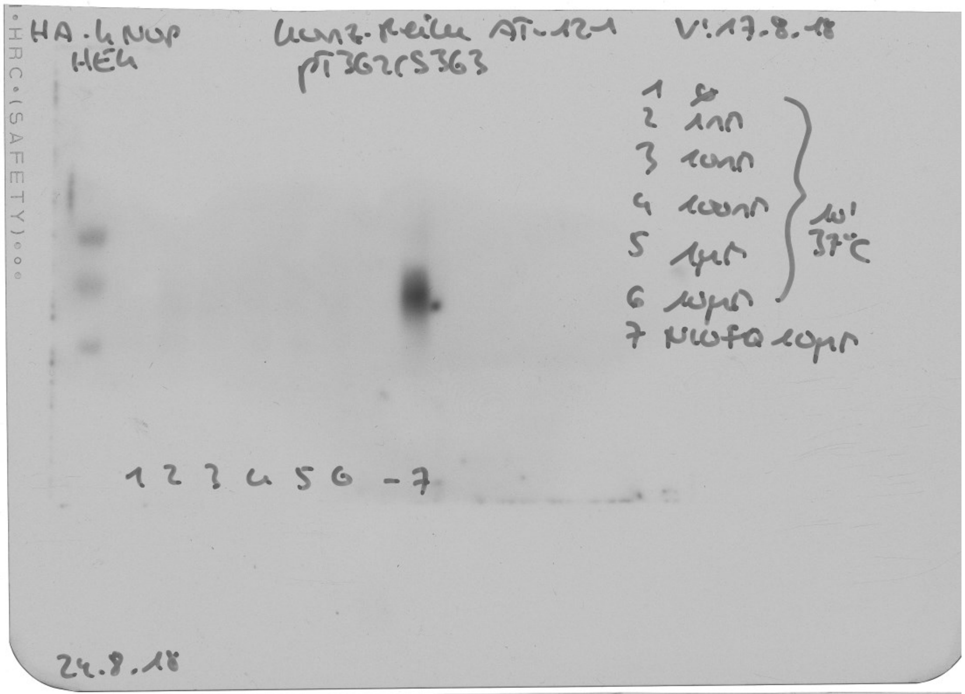
HA-hNOP
HEK
AT-121
pT362/S363
24.8.18

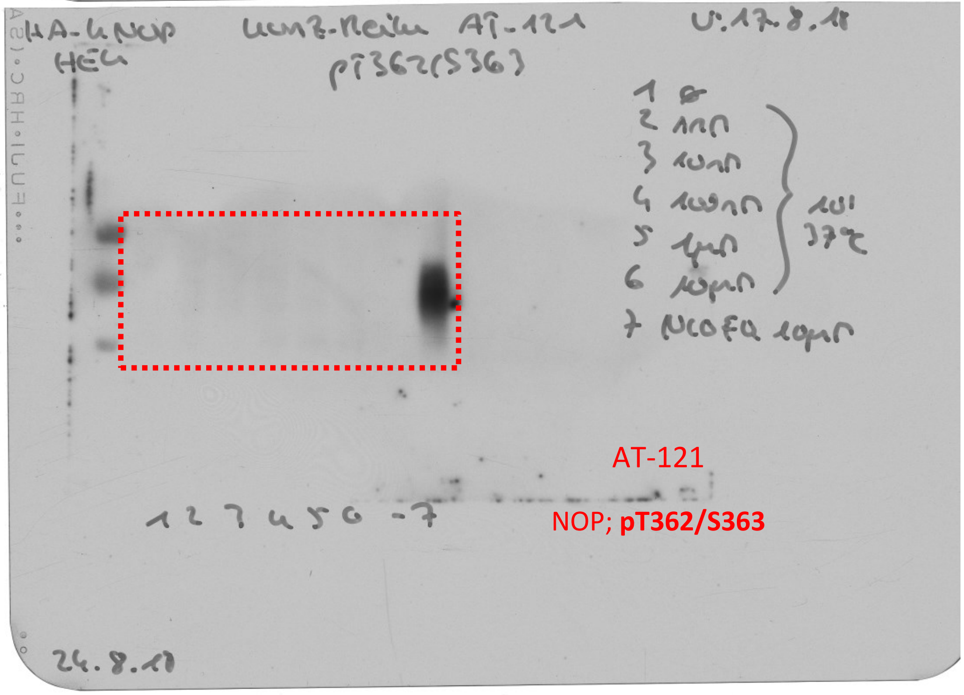
HA-hNOP
HEK
AT-121
pT362/S363
AT-121
NOP; pT362/S363
24.8.18

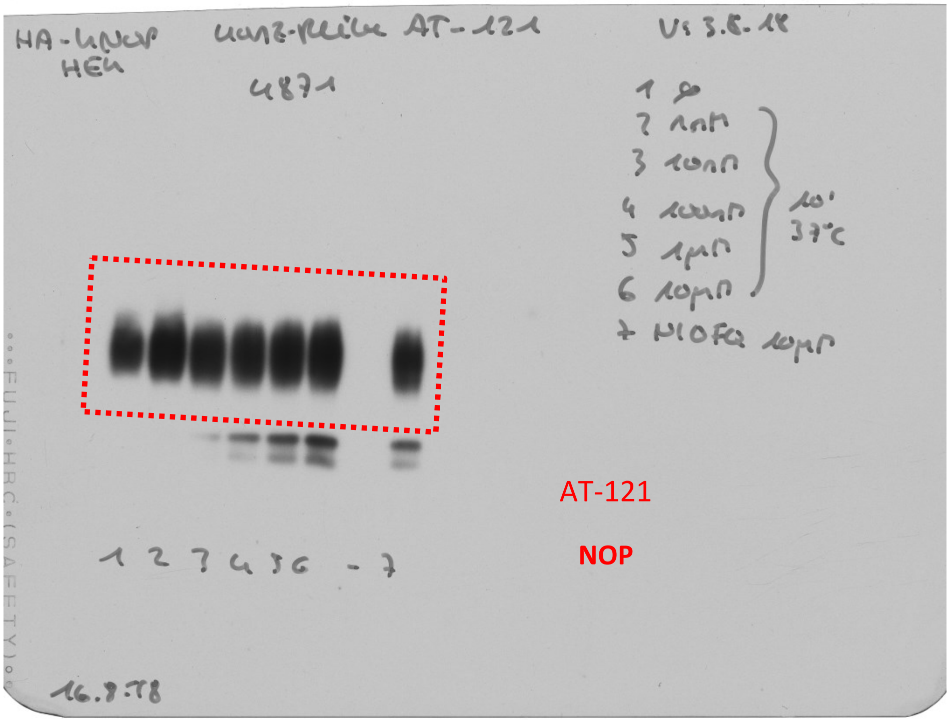
HA-hNOP
HEK
Konz-Reihe AT-121
V: 3.8.18
4871
1 ø
2 1nM
3 10nM
4 100nM
5 1µM
6 10µM
10'
37°C
7 N/OFQ 10µM
1 2 3 4 5 6 - 7
16.8.18
AT-121
NOP

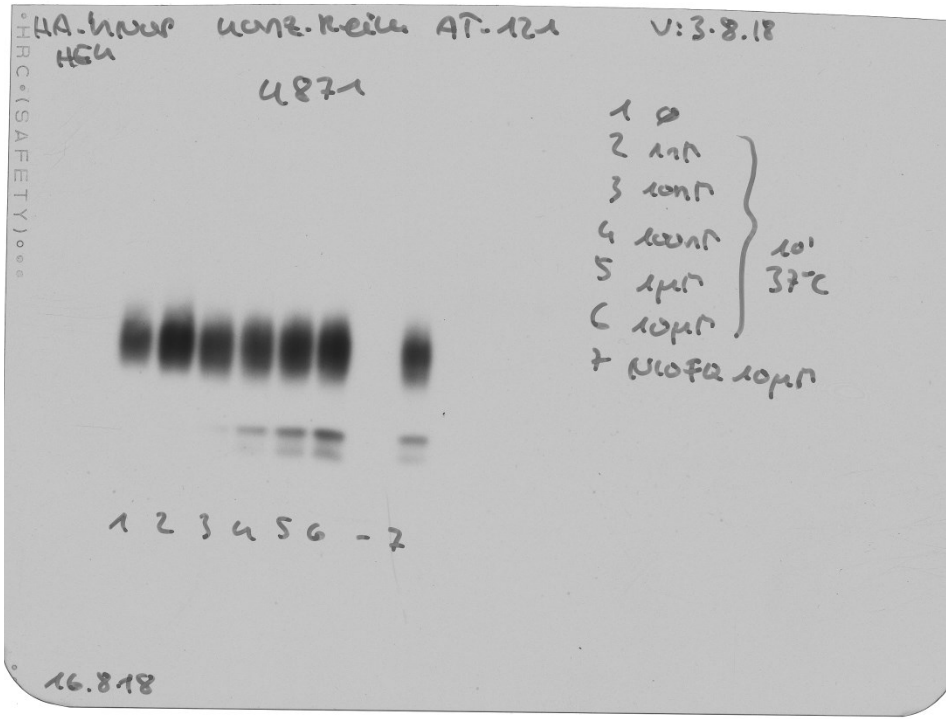
HA-hNOP
HEK
Konz-Reihe AT-121
V: 3.8.18
4871
1 ø
2 1nM
3 10nM
4 100nM
5 1µM
6 10µM
10'
37°C
7 N/OFQ 10µM
1 2 3 4 5 6 - 7
16.8.18

AT-121

<u>MOP</u>

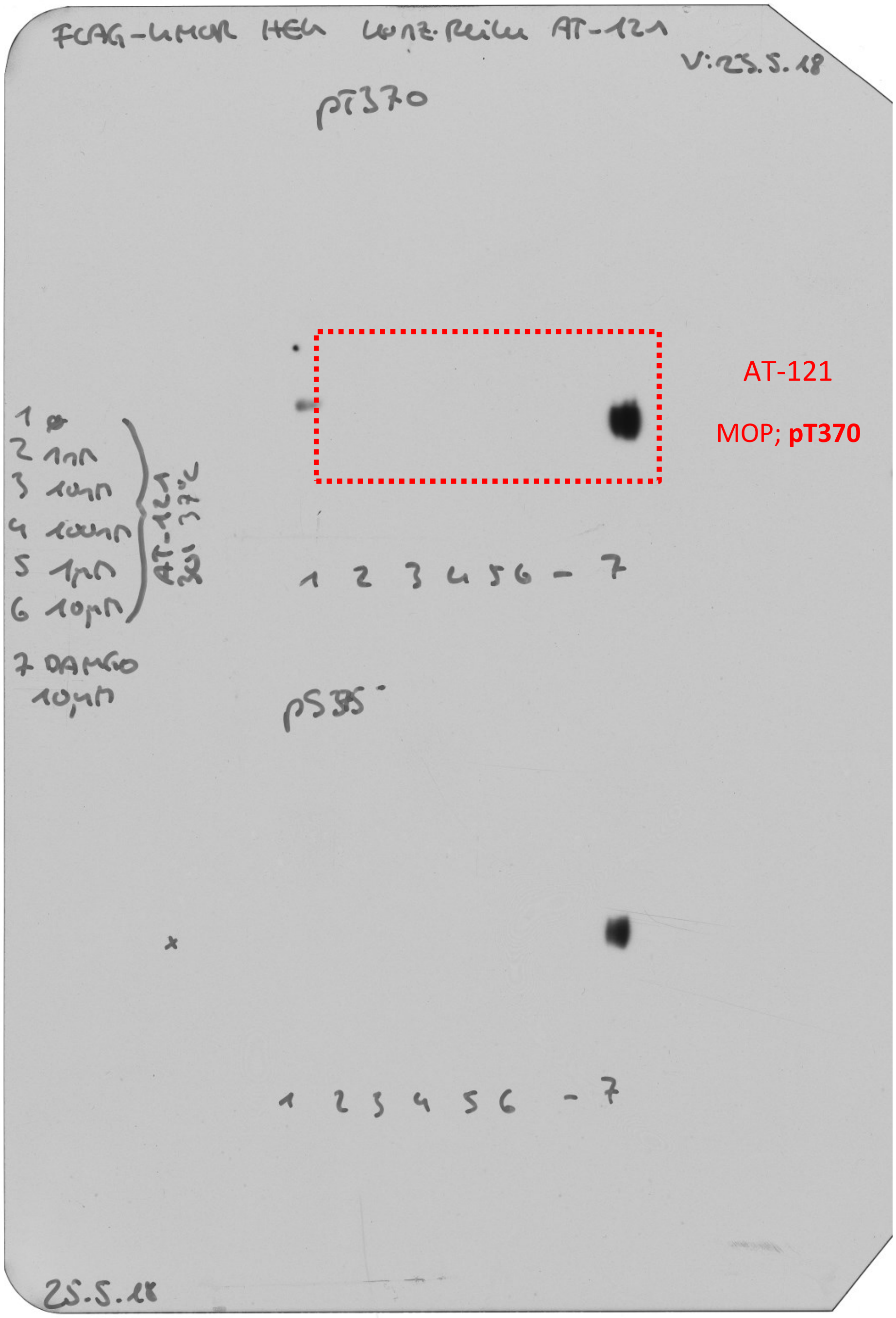

AT-121

MOP; **pT370**

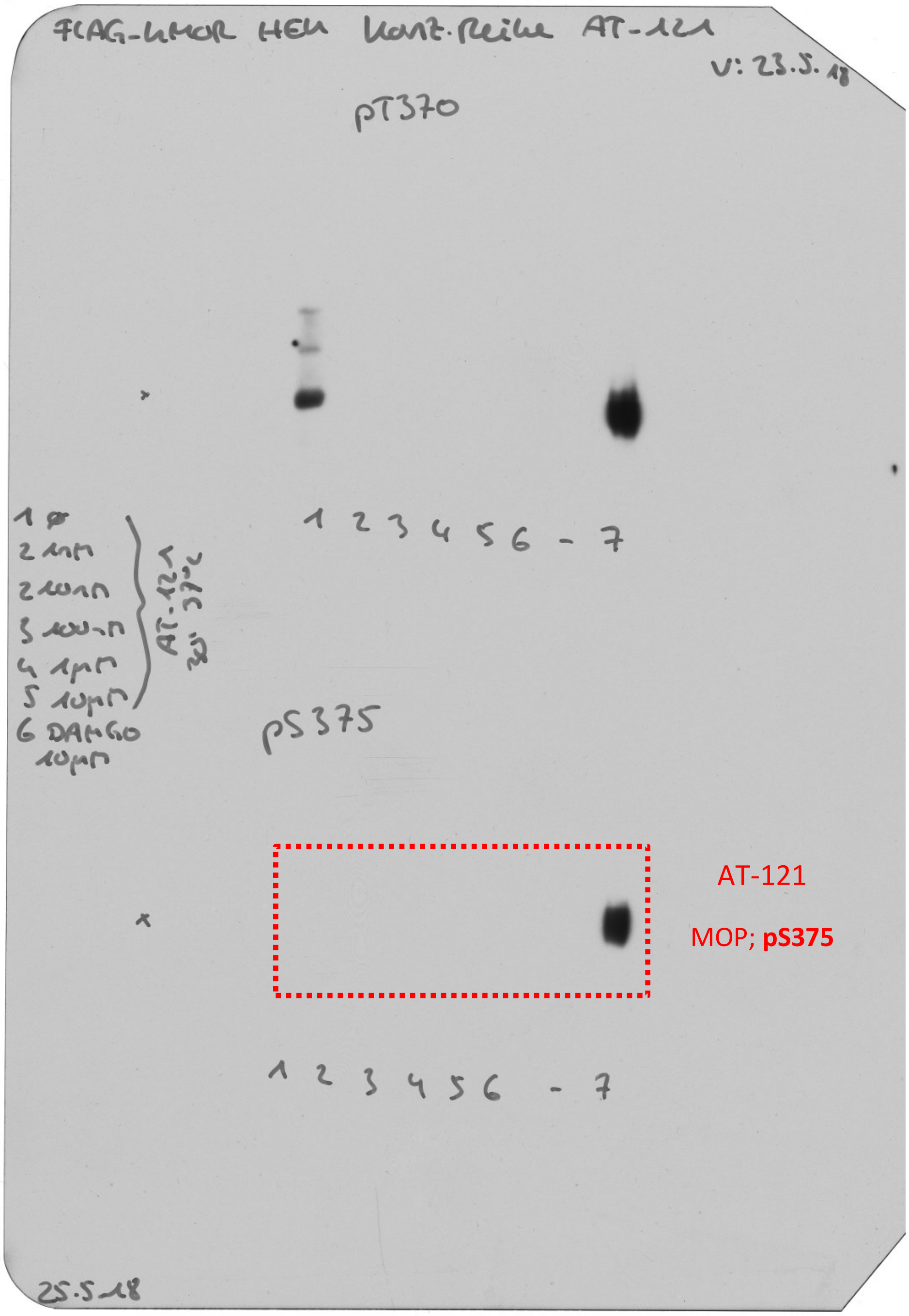
FLAG-hMOR HEK Konz.Reihe AT-121
V: 23.5.18
pT370
1 2 3 4 5 6 - 7
1 ø
2 1nM
2 10nM
3 100nM
4 1µM
5 10µM
6 DAMGO 10µM
AT-121 30' 37°C
pS375
AT-121
MOP; pS375
1 2 3 4 5 6 - 7
25.5.18

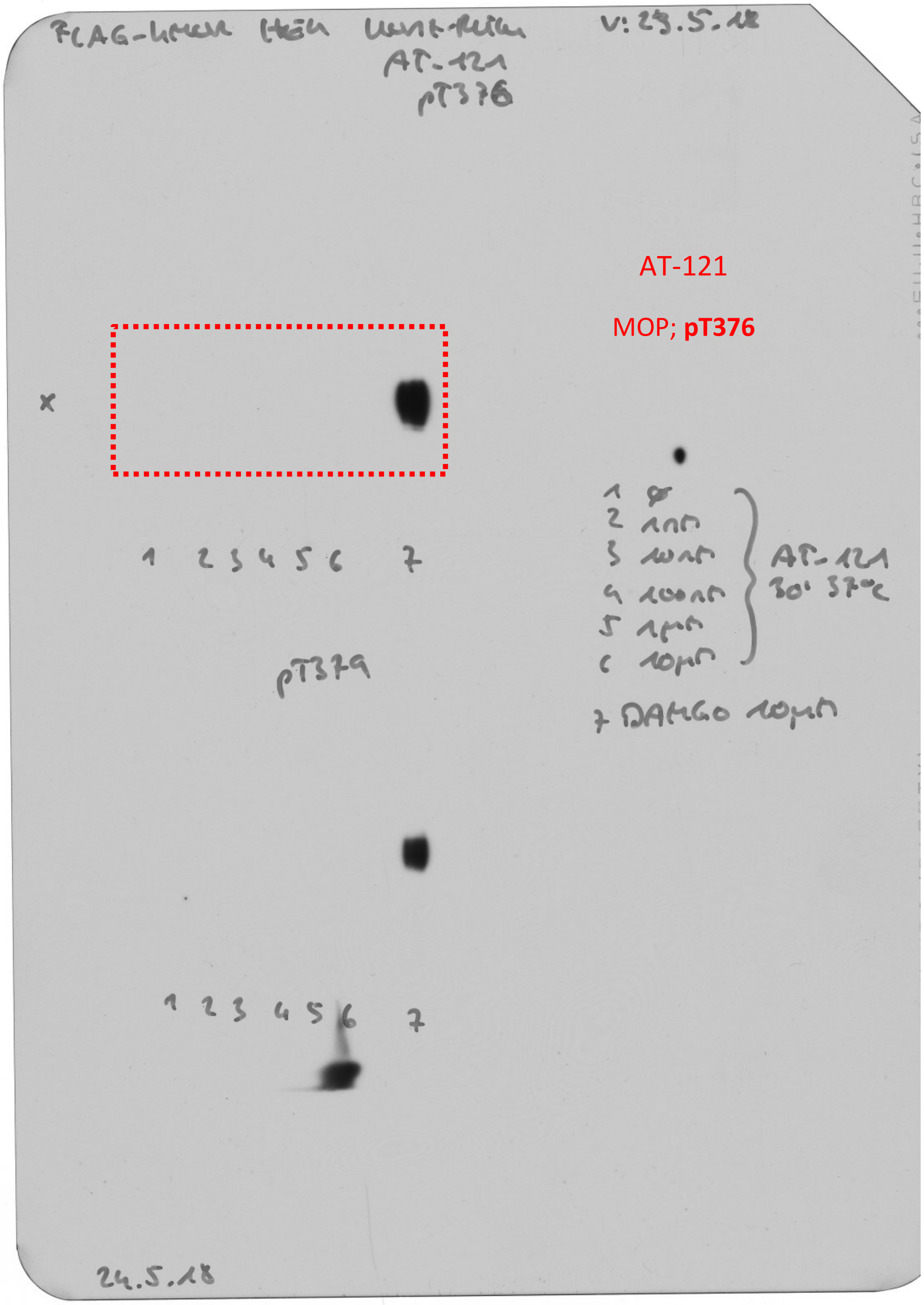
AT-121
pT376
V: 23.5.18
AT-121
MOP; pT376
1 2 3 4 5 6 7
pT379
1 2 3 4 5 6 7
1 ø
2 1nM
3 10nM
4 100nM
5 1µM
6 10µM
AT-121
30' 37°C
7 DAMGO 10µM
24.5.18

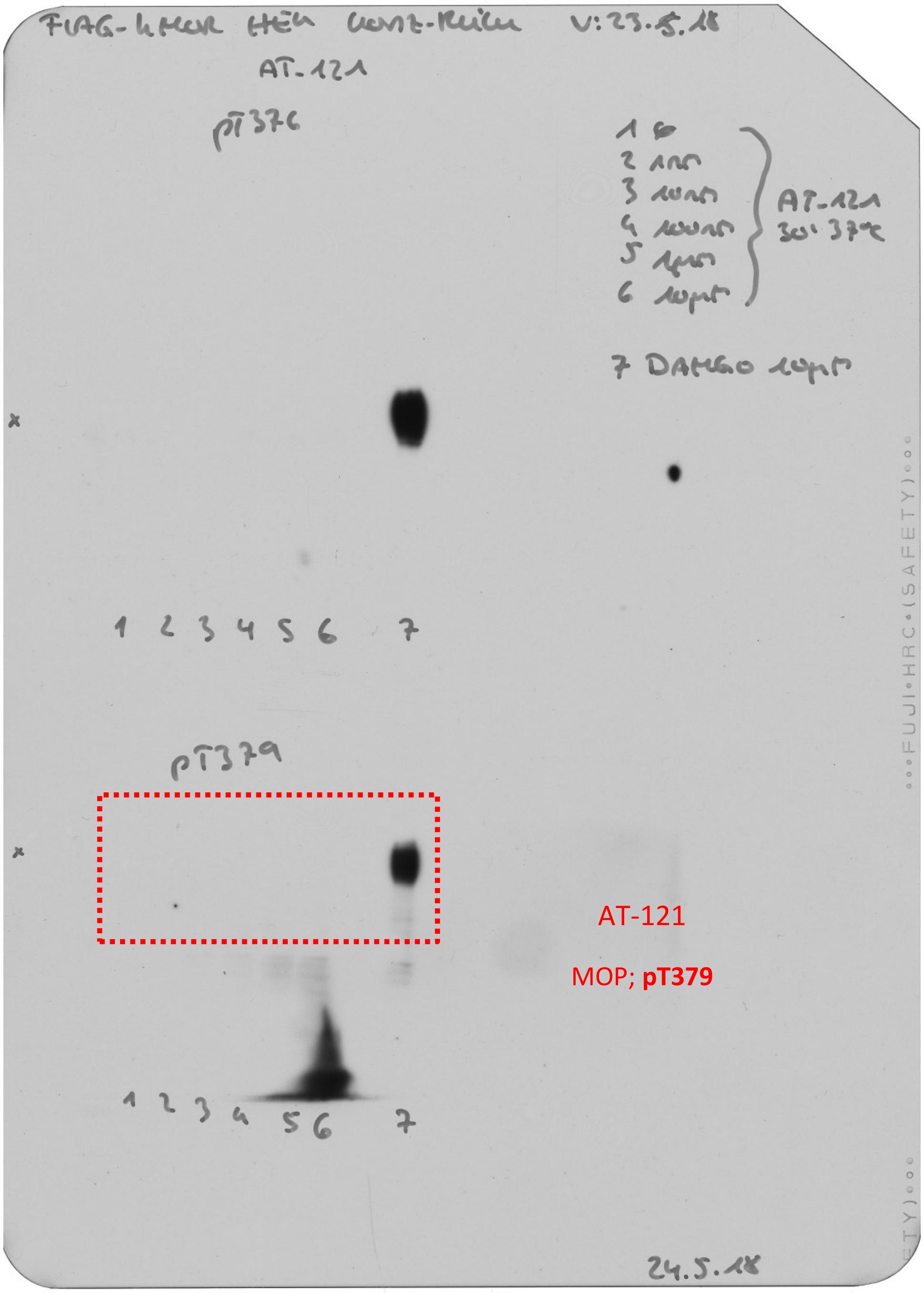
V:23.5.18
AT-121
pT376
1 0
2 1nM
3 10nM
4 100nM
5 1μM
6 10μM
AT-121 30' 37°C
7 DAMGO 10μM
1 2 3 4 5 6 7
pT379
AT-121
MOP; **pT379**
1 2 3 4 5 6 7
24.5.18
FUJI•HRC•(SAFETY)

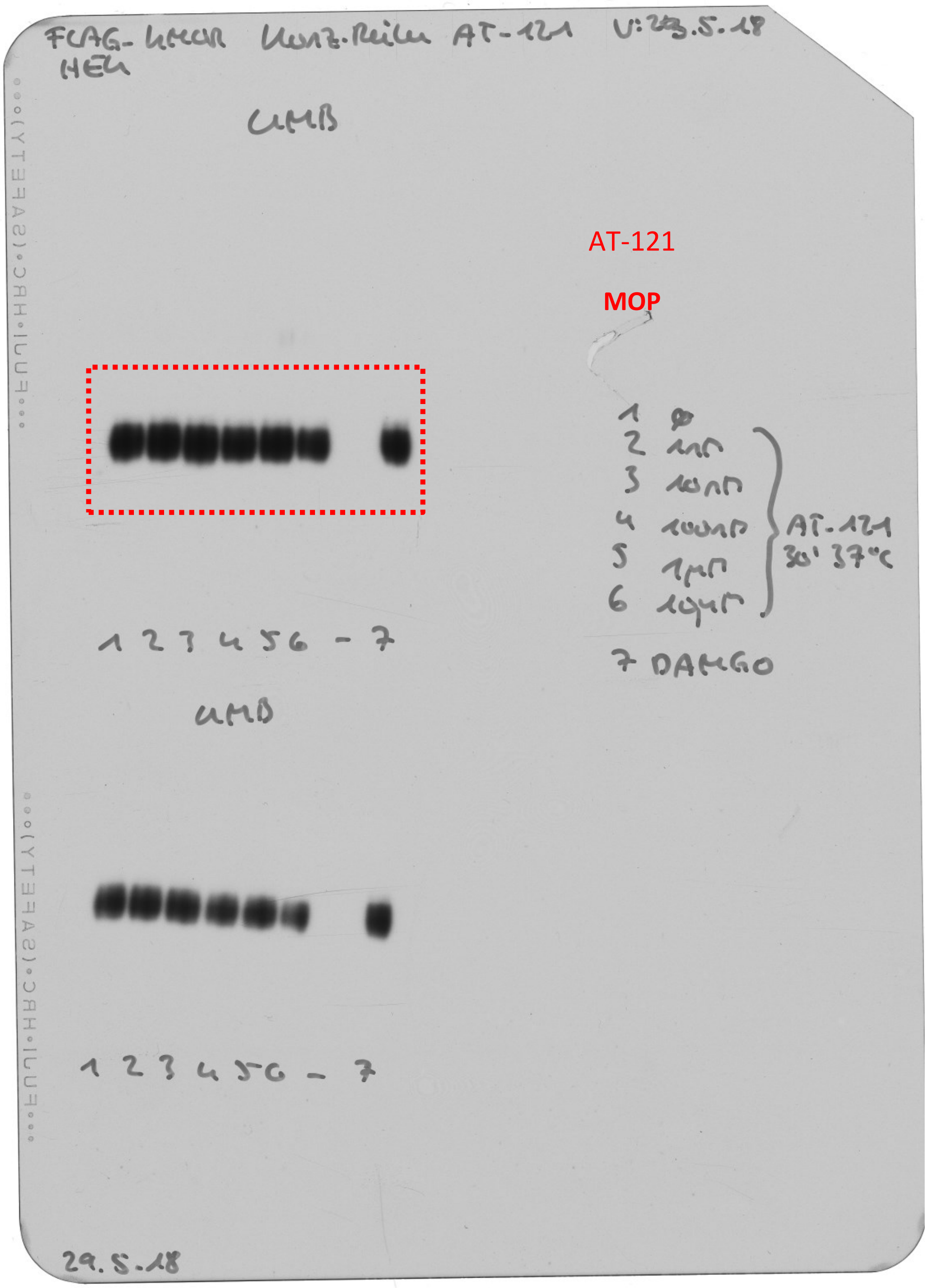
AT-121
MOP
1 ∅
2 1nM
3 10nM
4 100nM
5 1µM
6 10µM
AT-121 30' 37°C
7 DAMGO
1 2 3 4 5 6 - 7
1 2 3 4 5 6 - 7
29.5.18

Cebranopadol

NOP

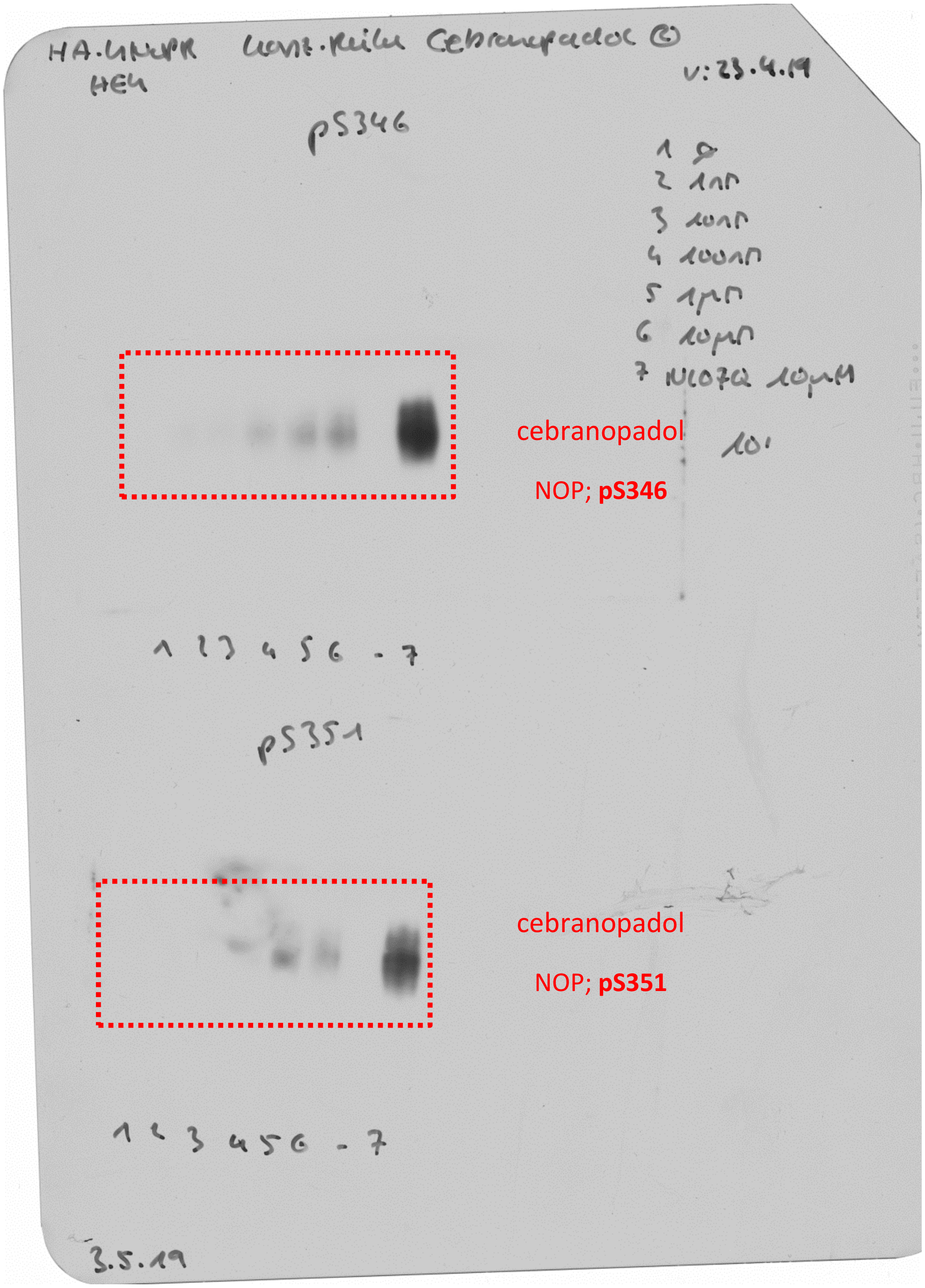
pS346
cebranopadol
NOP; pS346
pS351
cebranopadol
NOP; pS351
3.5.19

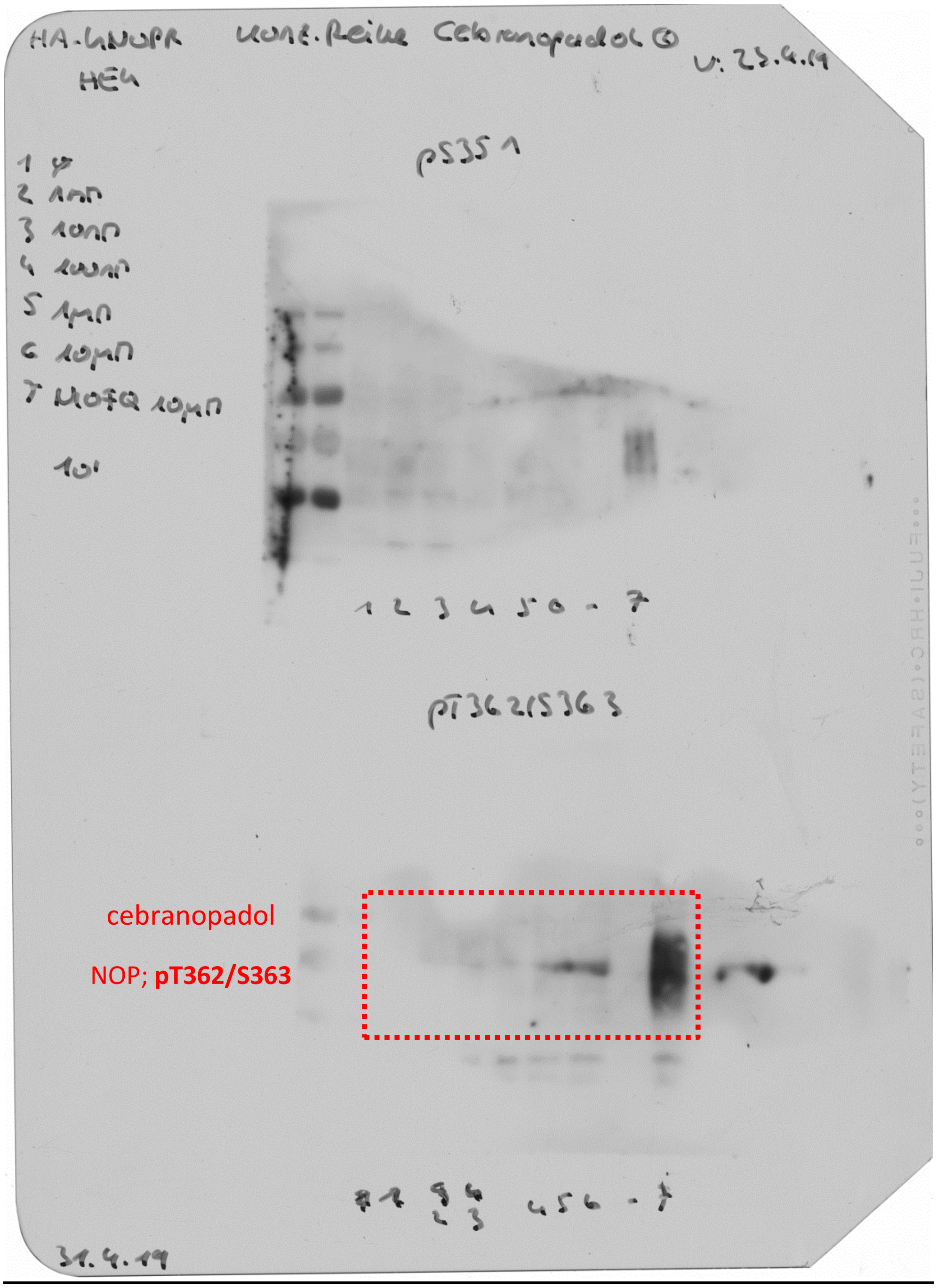
pS351
pT362/S363
cebranopadol
NOP; pT362/S363

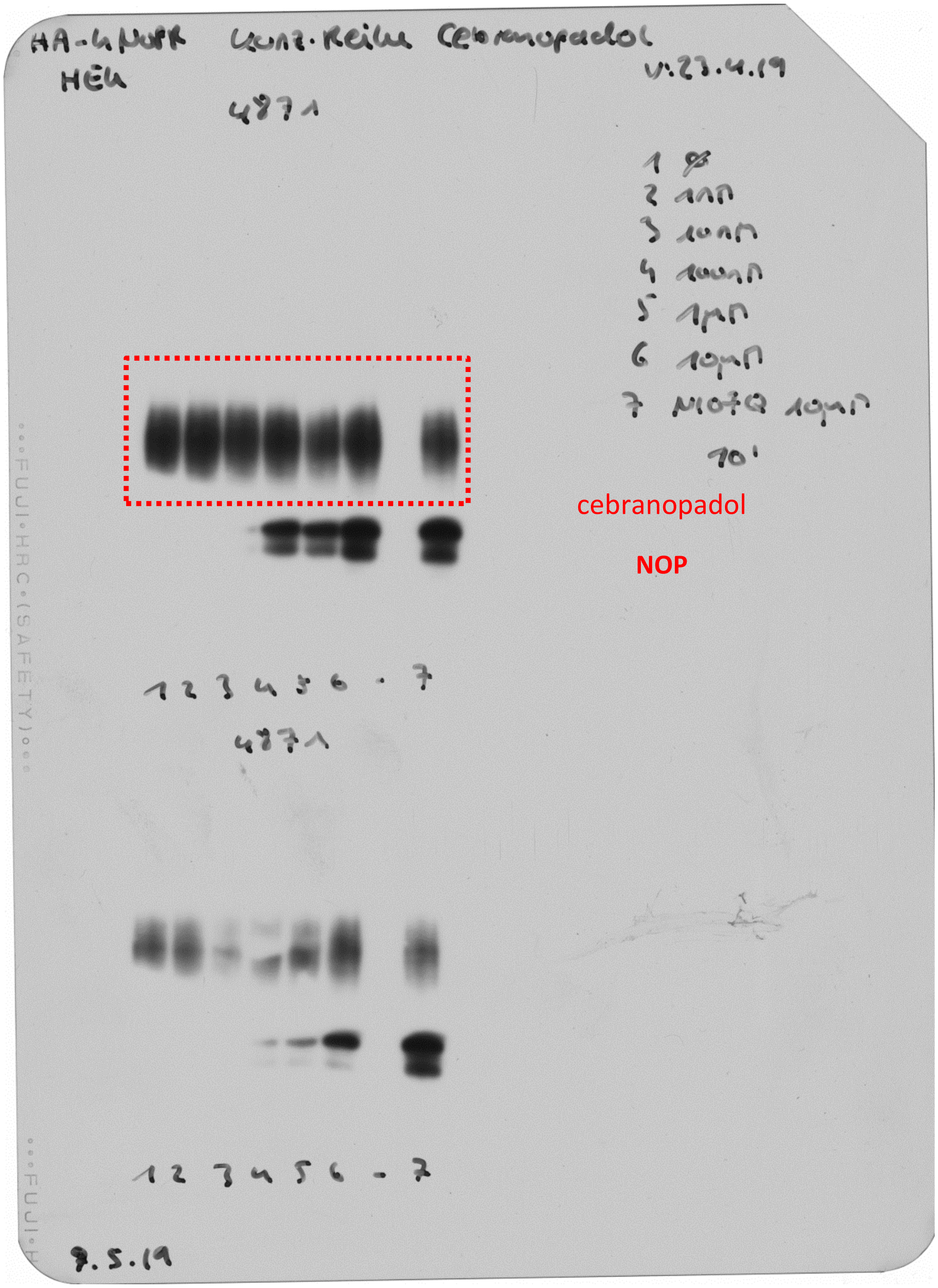
HA-hNOPR Konz-Reihe Cebranopadol
HEK
4871
1 Ø
2 1nM
3 10nM
4 100nM
5 1µM
6 10µM
7 N/OFQ 10µM
90'
cebranopadol
NOP
1 2 3 4 5 6 - 7
4871
1 2 3 4 5 6 - 7
9.5.19

Cebranopadol

<u>MOP</u>

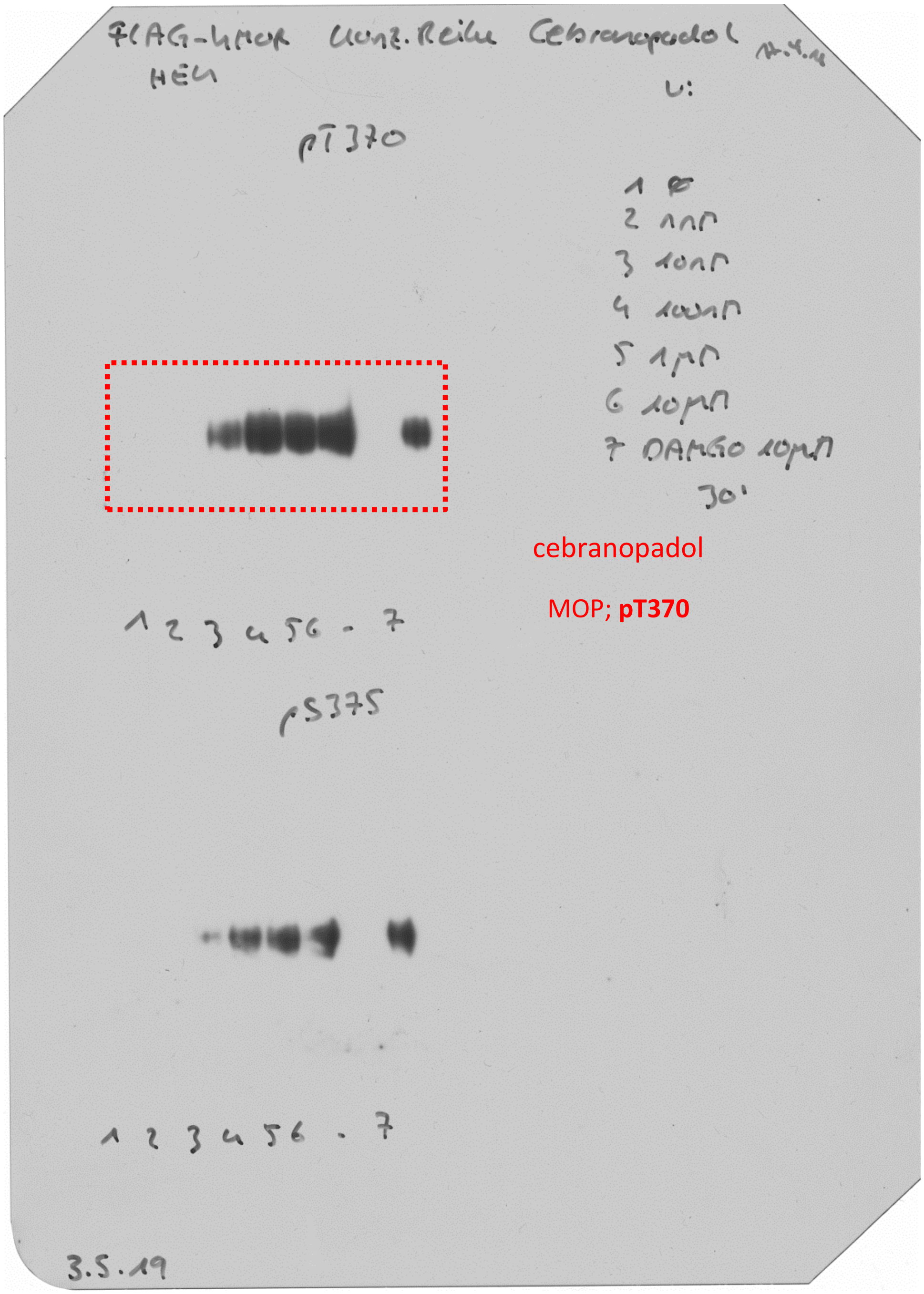
FLAG-hMOR Konz.Reihe Cebranopadol
HEK
pT370
1 ø
2 1nM
3 10nM
4 100nM
5 1µM
6 10µM
7 DAMGO 10µM
30'
cebranopadol
MOP; **pT370**
1 2 3 4 5 6 - 7
pS375
1 2 3 4 5 6 - 7
3.5.19

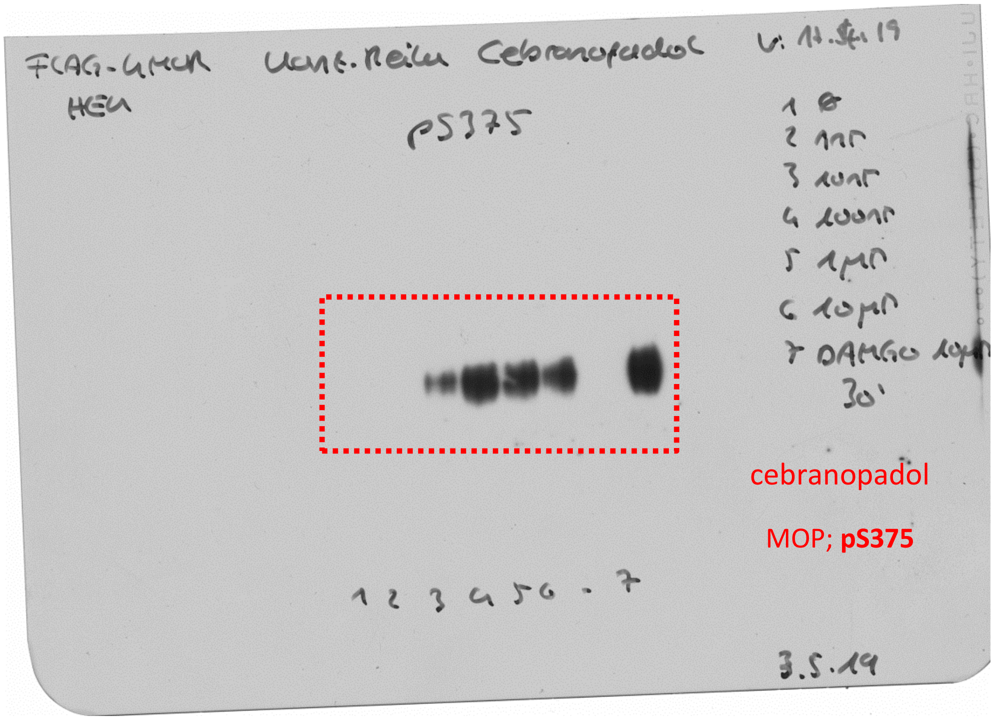
pS375
cebranopadol
MOP; pS375
1 2 3 4 5 6 7
3.5.19

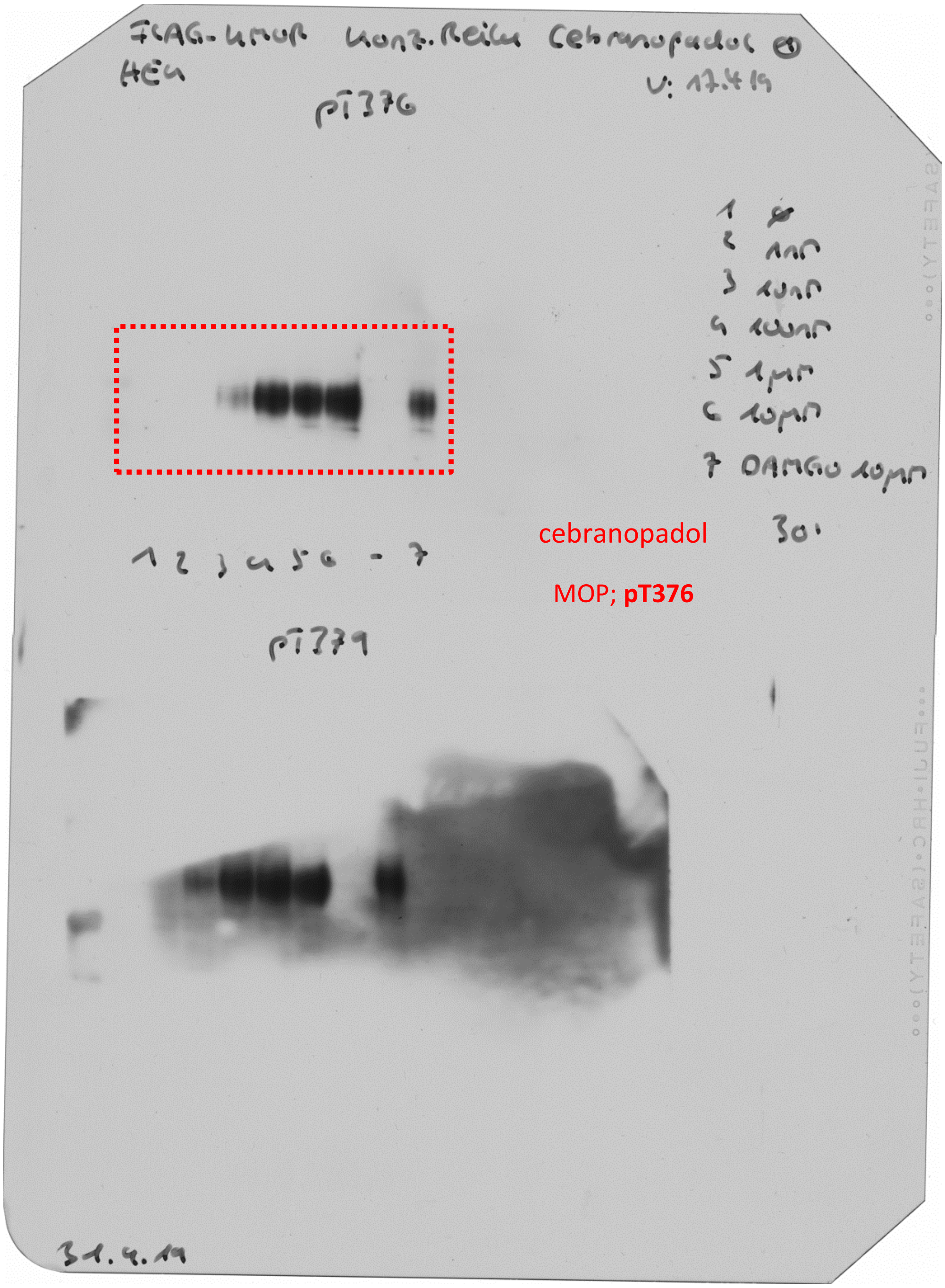
FLAG-hMOR Konz.Reihe Cebranopadol
HEK
pT376
1 ø
2 1nM
3 10nM
4 100nM
5 1µM
6 10µM
7 DAMGO 10µM
30'
1 2 3 4 5 6 - 7
cebranopadol
MOP; pT376
pT379

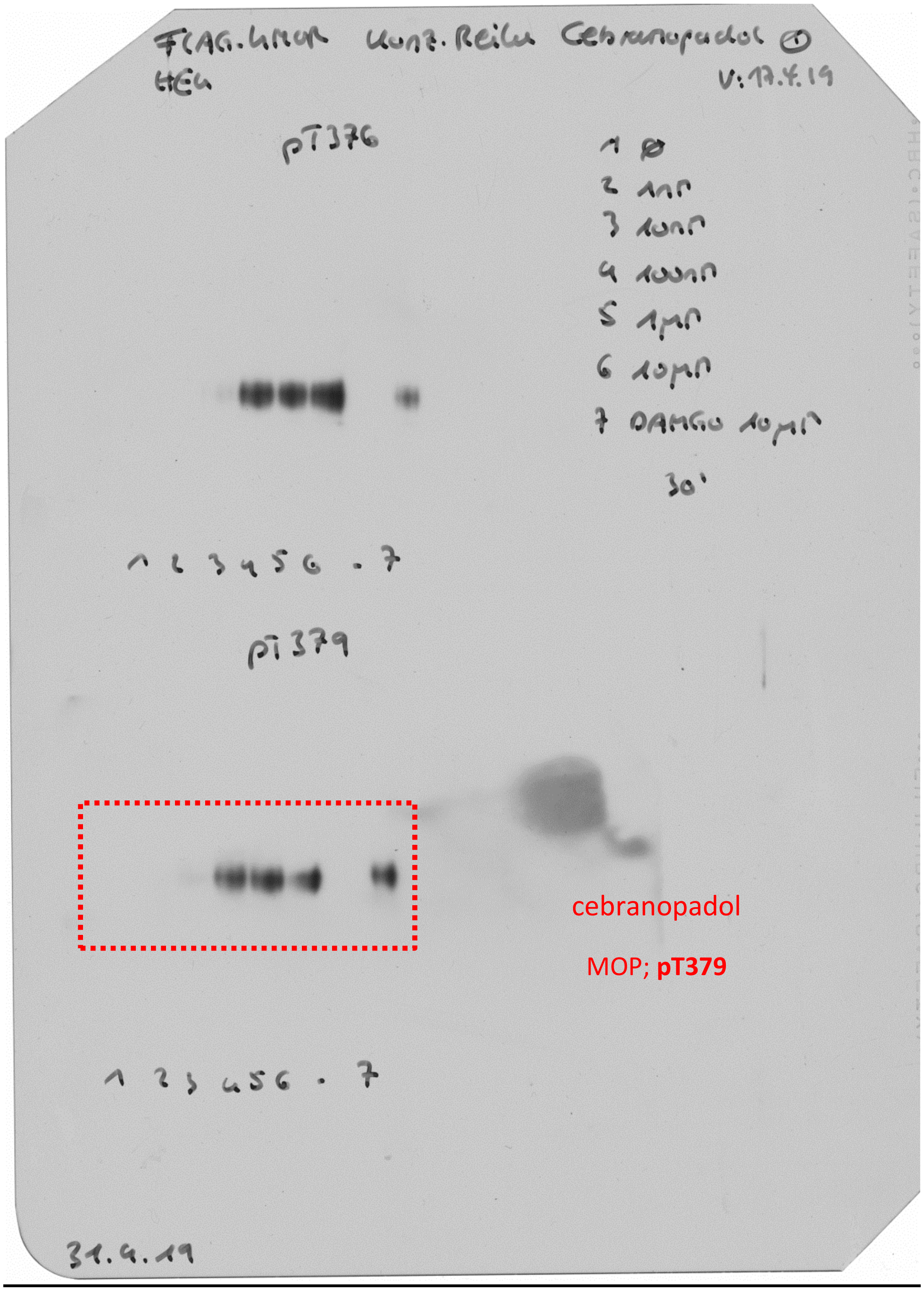
pT376
pT379
7 DAMGO 10µM
30'
cebranopadol
MOP; pT379

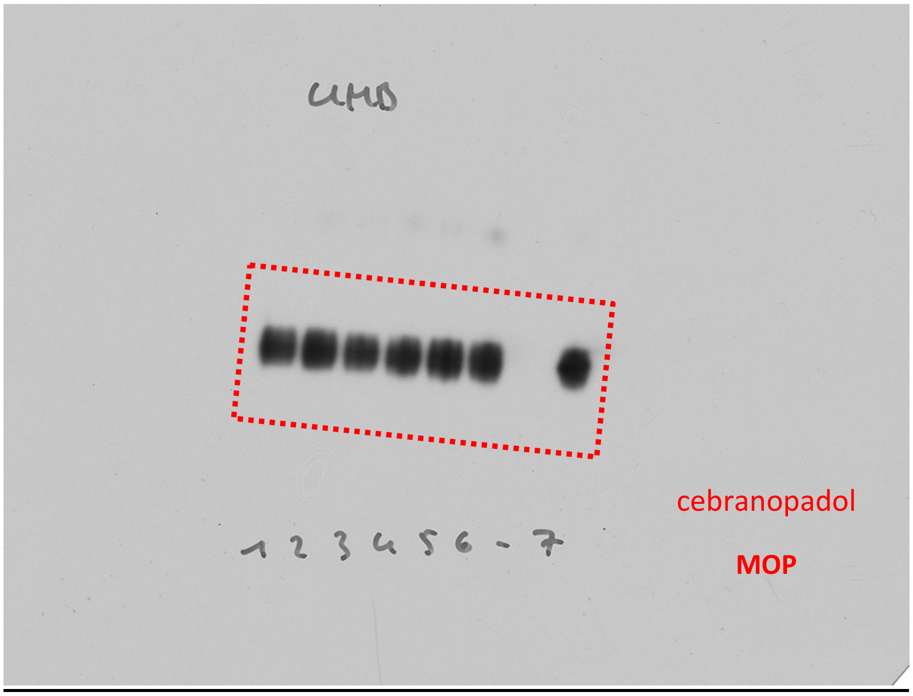

1 2 3 4 5 6 - 7
cebranopadol
MOP

N/OFQ

<u>NOP</u>

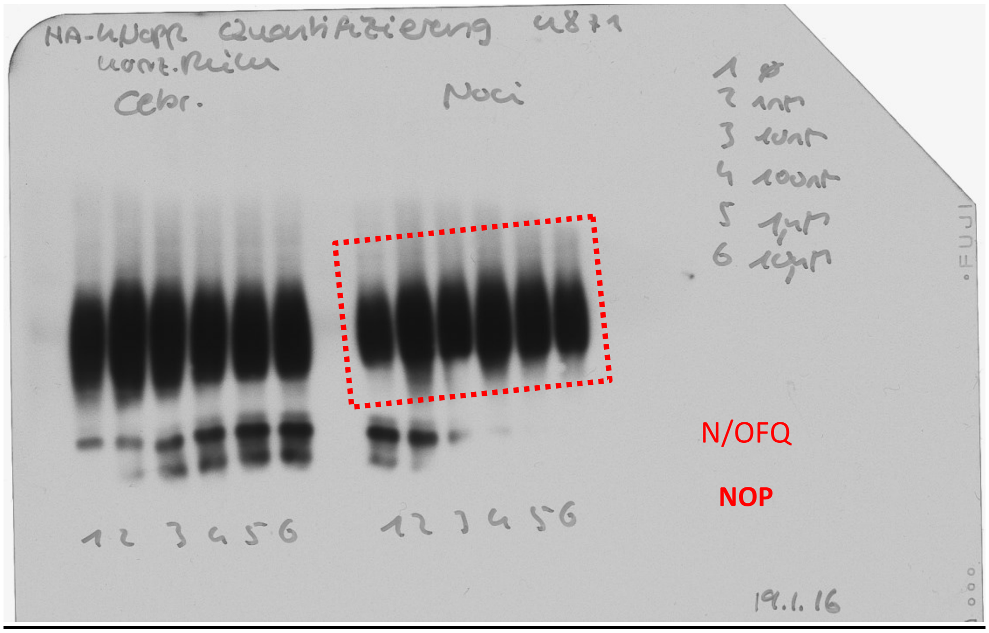
Noci
N/OFQ
NOP
1 2 3 4 5 6
1 2 3 4 5 6
19.1.16
FUJI

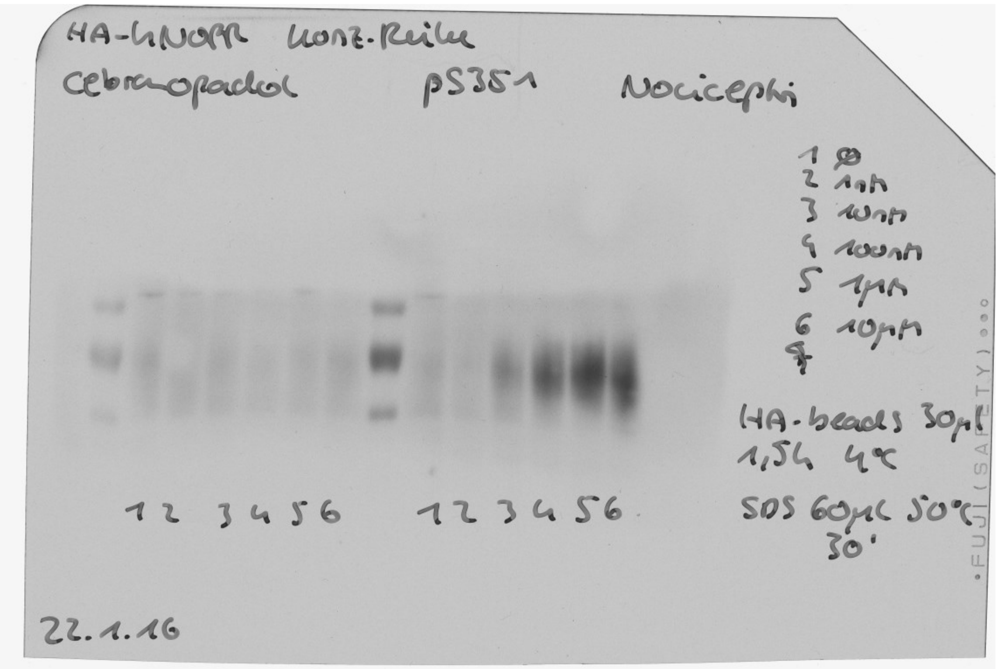
HA-hNOPR Konz.-Reihe
Cebranopadol pS351 Nociceptin
1 ø
2 1nM
3 10nM
4 100nM
5 1µM
6 10µM
HA-beads 30µl
1,5h 4°C
SDS 60µl 50°C
30'
1 2 3 4 5 6 1 2 3 4 5 6
22.1.16
FUJI (SAFETY)

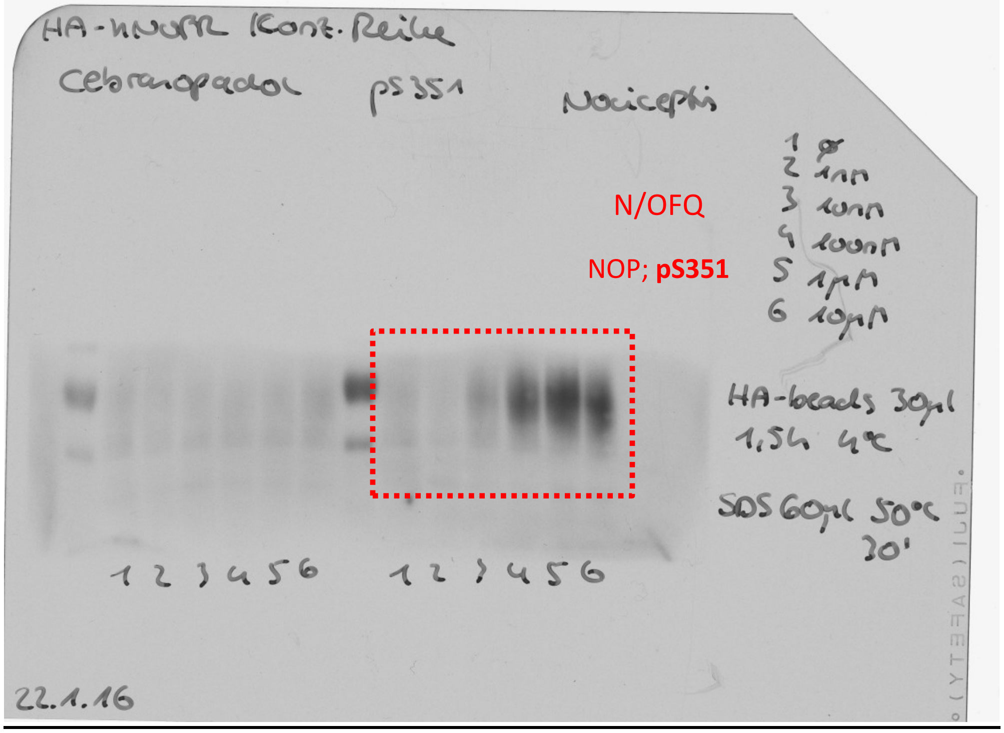
HA-hNOPR Konz.-Reihe
Cebranopadol pS351 Nociceptin
N/OFQ
NOP; pS351
1 ø
2 1nM
3 10nM
4 100nM
5 1µM
6 10µM
HA-beads 30µl
1,5h 4°C
SDS 60µl 50°C
30'
1 2 3 4 5 6 1 2 3 4 5 6
22.1.16

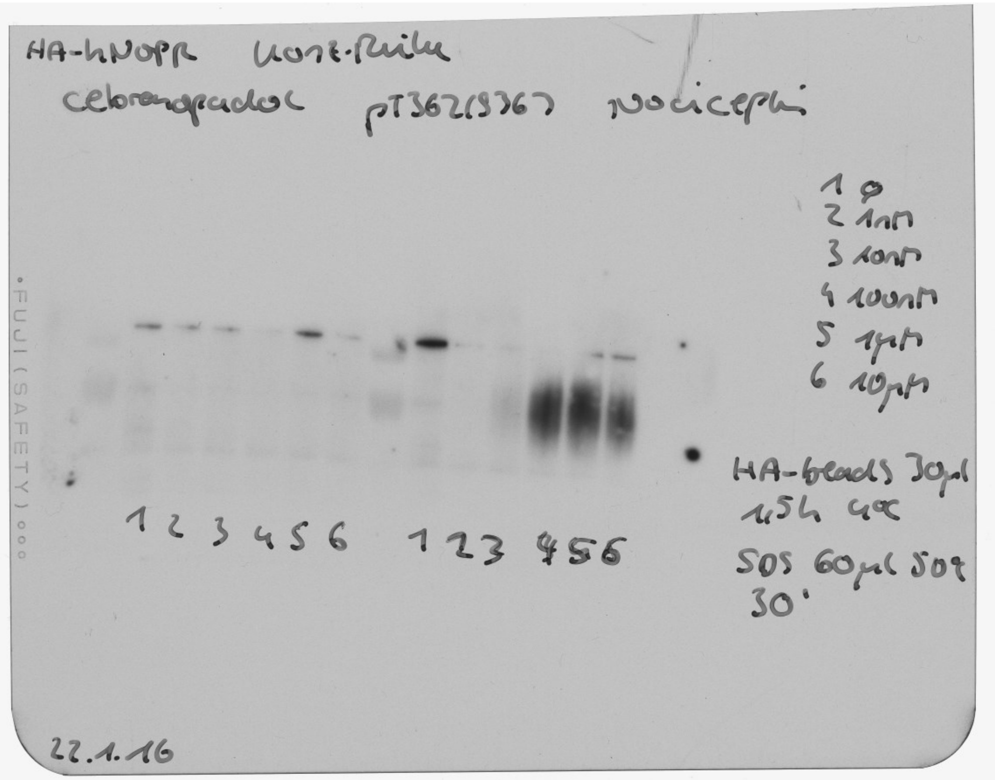
HA-hNOPR Konz.-Reihe
Cebranopadol pT362/S363 Nocicepti
1 ∅
2 1nM
3 10nM
4 100nM
5 1µM
6 10µM
HA-beads 30µl
1,5h 4°C
SDS 60µl 50°C
30'
1 2 3 4 5 6 1 2 3 4 5 6
22.1.16

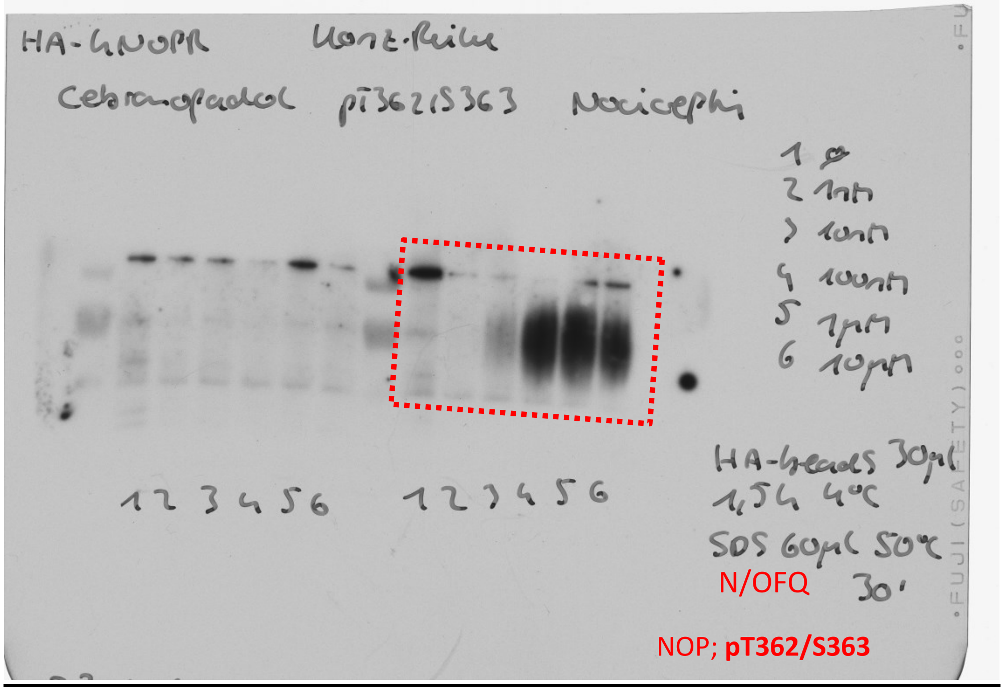
HA-hNOPR Konz.-Reihe
Cebranopadol pT362/S363 Nocicepti
1 ∅
2 1nM
3 10nM
4 100nM
5 1µM
6 10µM
HA-beads 30µl
1,5h 4°C
SDS 60µl 50°C
30'
1 2 3 4 5 6 1 2 3 4 5 6
N/OFQ
NOP; pT362/S363

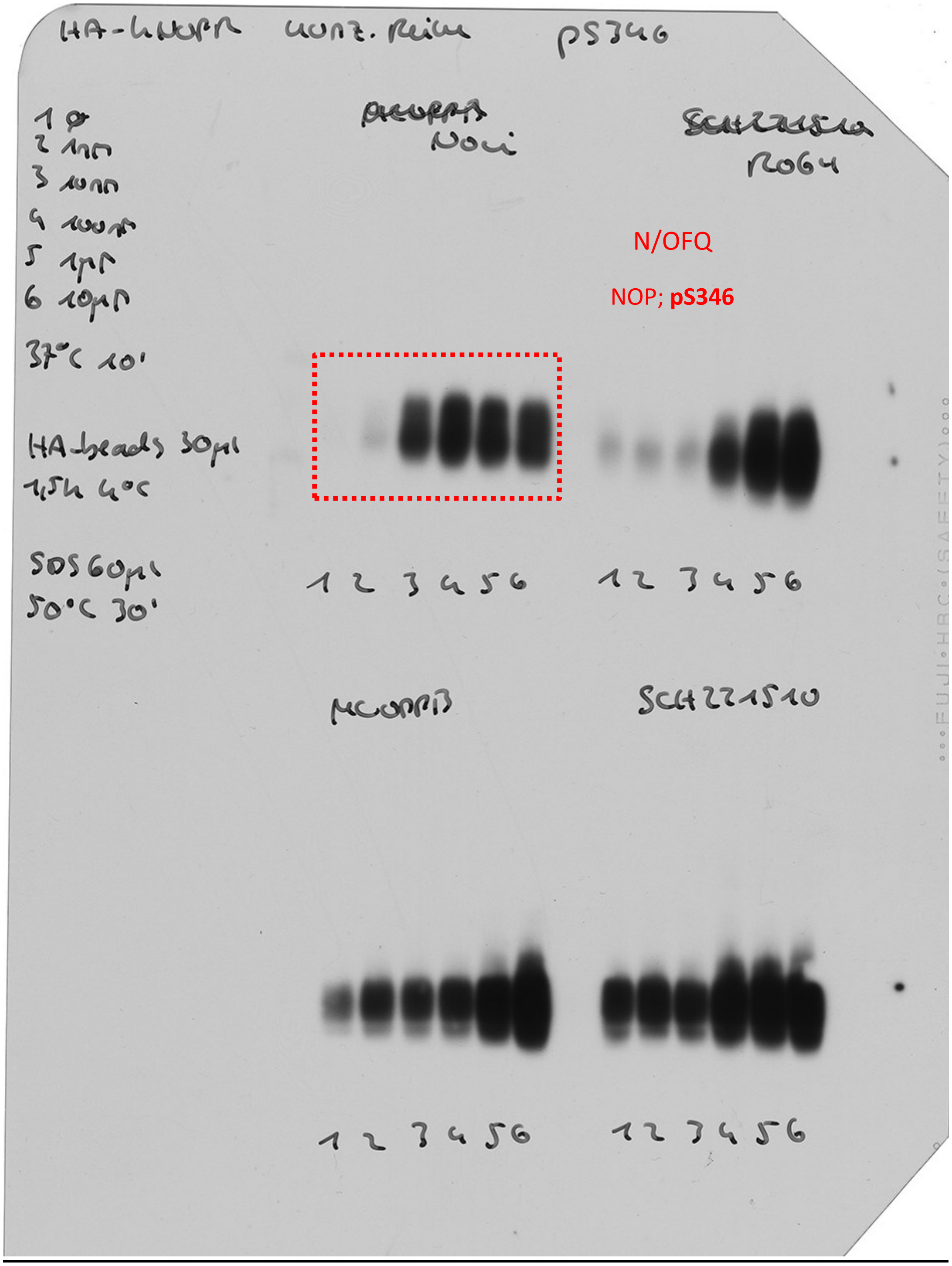

pS346
1 ø
2 1nM
3 10nM
4 100nM
5 1µM
6 10µM
37°C 10'
HA-beads 30µl
1,5h 4°C
SDS 60µl
50°C 30'
N/OFQ
NOP; pS346
1 2 3 4 5 6
1 2 3 4 5 6
MCOPPB
SCH221510
1 2 3 4 5 6
1 2 3 4 5 6

DAMGO

MOP

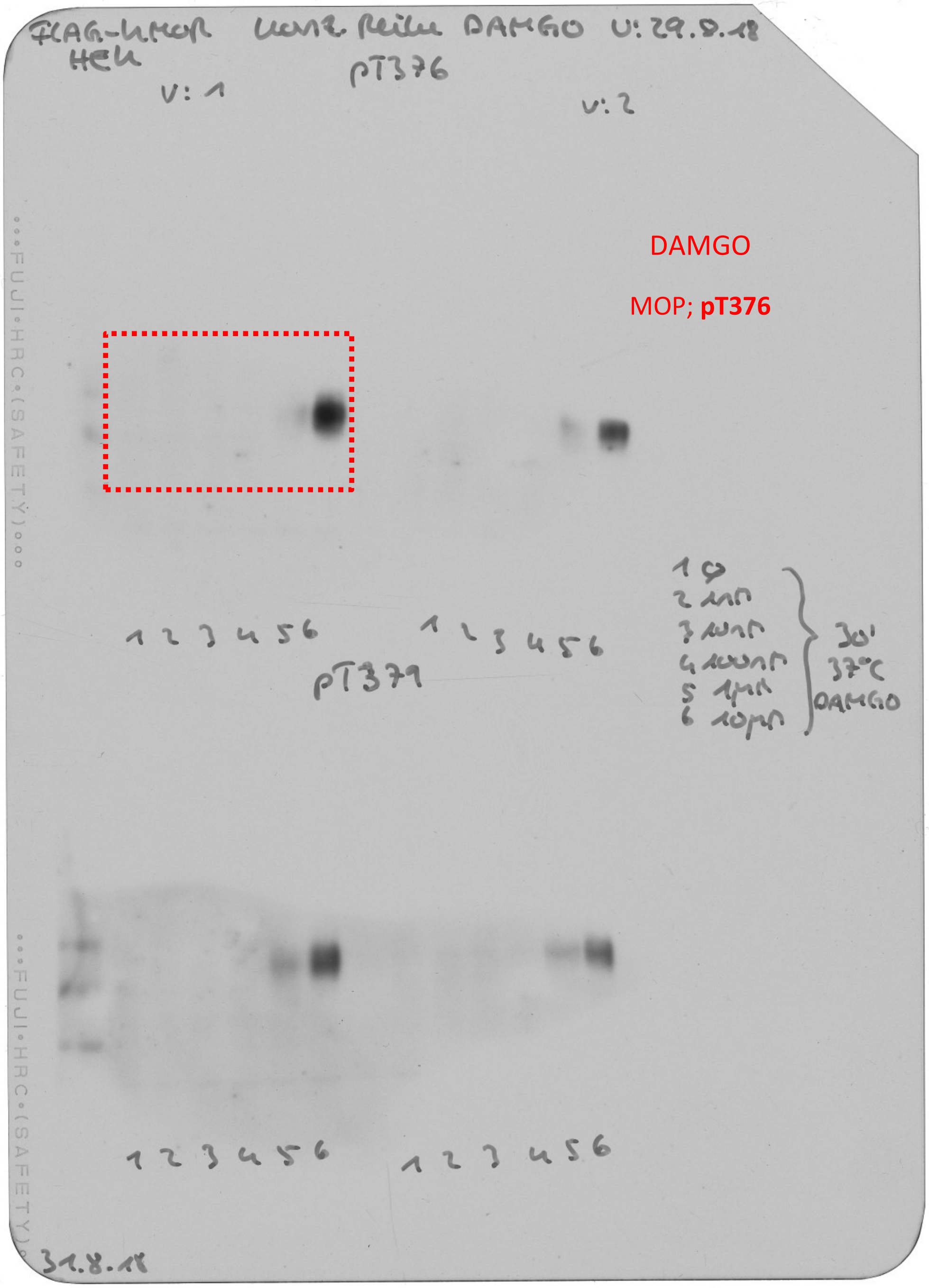
FLAG-hMOR
HEK
Konz. Reihe DAMGO
V: 29.8.18
pT376
V: 1
V: 2
DAMGO
MOP; pT376
1 2 3 4 5 6
1 2 3 4 5 6
pT379
1 Ø
2 1nM
3 10nM
4 100nM
5 1µM
6 10µM
30'
37°C
DAMGO
1 2 3 4 5 6
1 2 3 4 5 6
31.8.18
FUJI•HRC•(SAFETY)

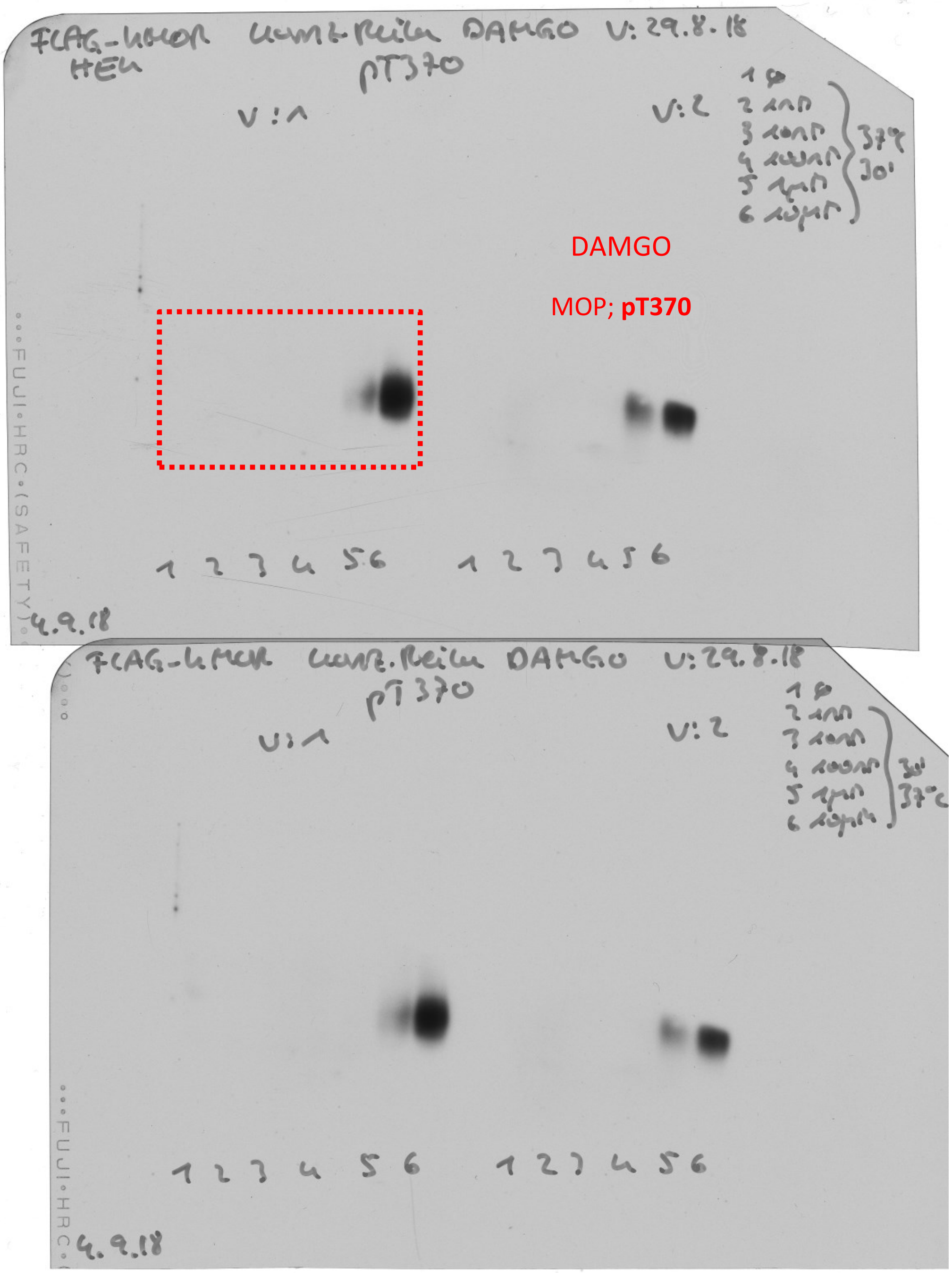
DAMGO
MOP; pT370
FLAG-hMOR
pT370
V:29.8.18
4.9.18
FUJI HRC (SAFETY)

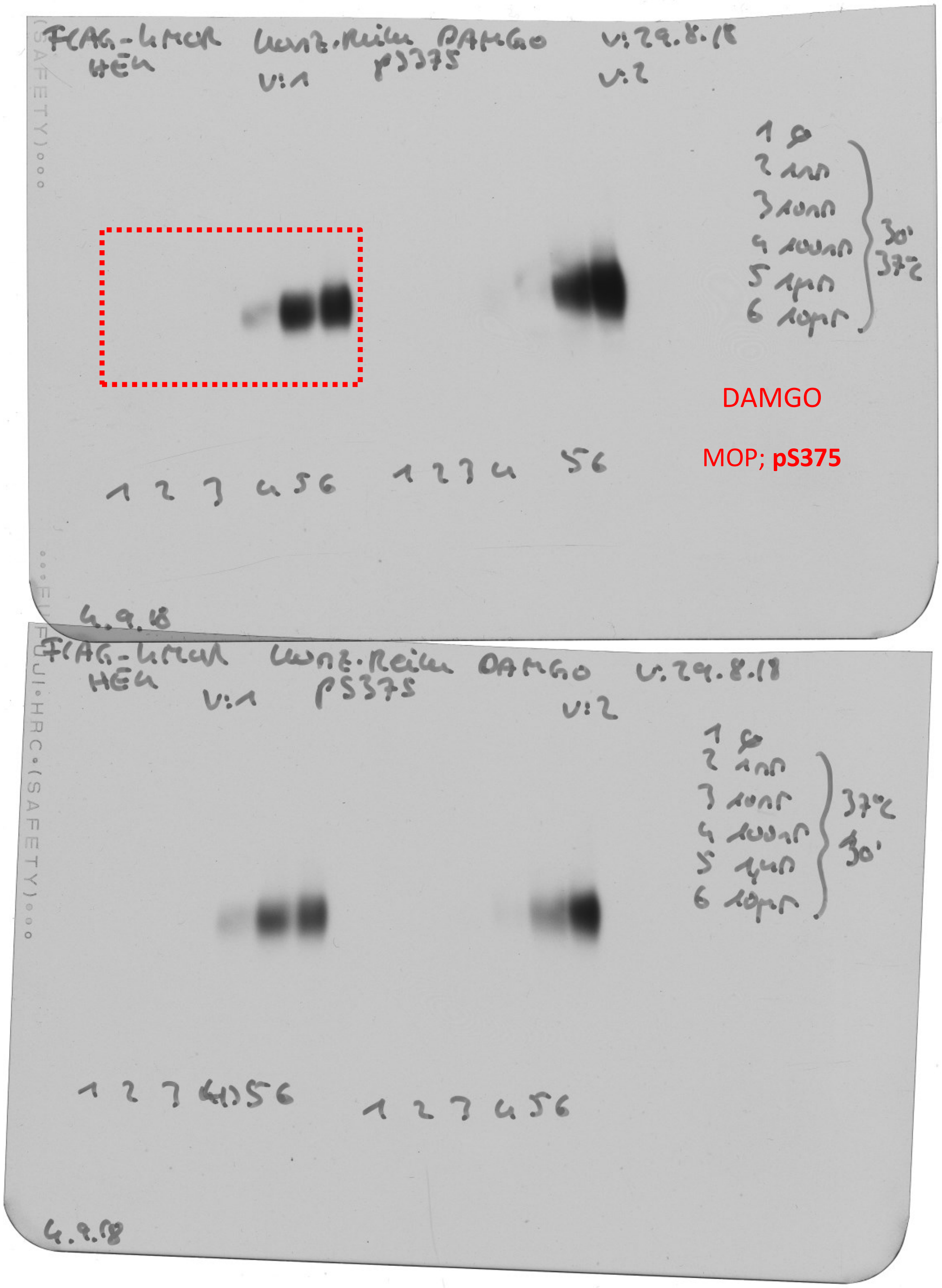
DAMGO
MOP; pS375
FLAG-hMOR HEK
DAMGO
pS375
v: 29.8.18
1 2 3 4 5 6
37°C
30'
4.9.18

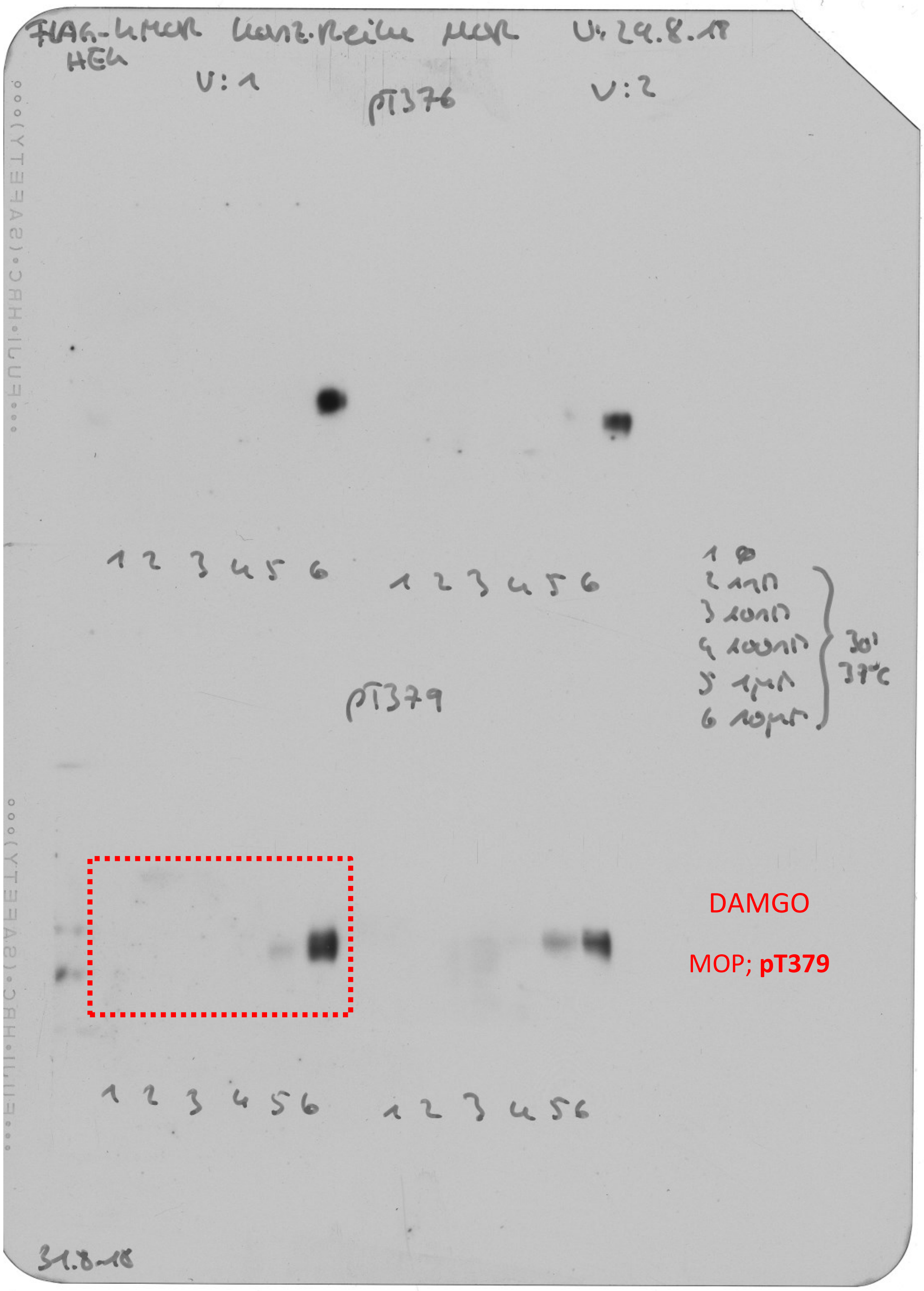
HEK
V:1
pT376
V:2
1 2 3 4 5 6
pT379
30'
37°C
DAMGO
MOP; pT379
31.8.18

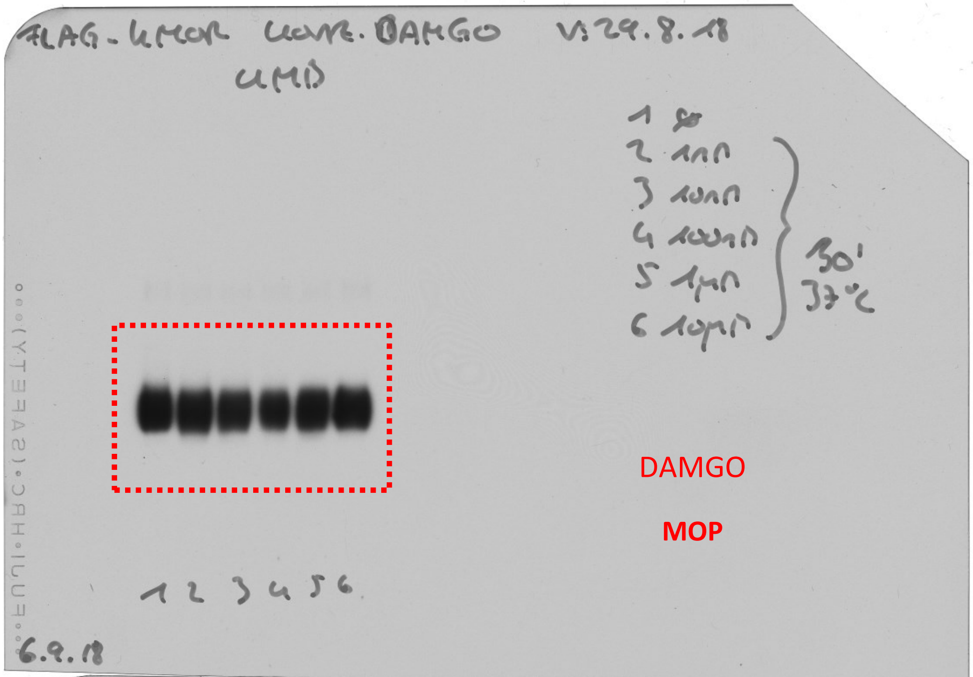
FLAG-hMOR
DAMGO
v: 29.8.18
1 ø
2 1nM
3 10nM
4 100nM
5 1µM
6 10µM
30'
37°C
1 2 3 4 5 6
6.9.18
FUJI•HRC•(SAFETY)
DAMGO
MOP

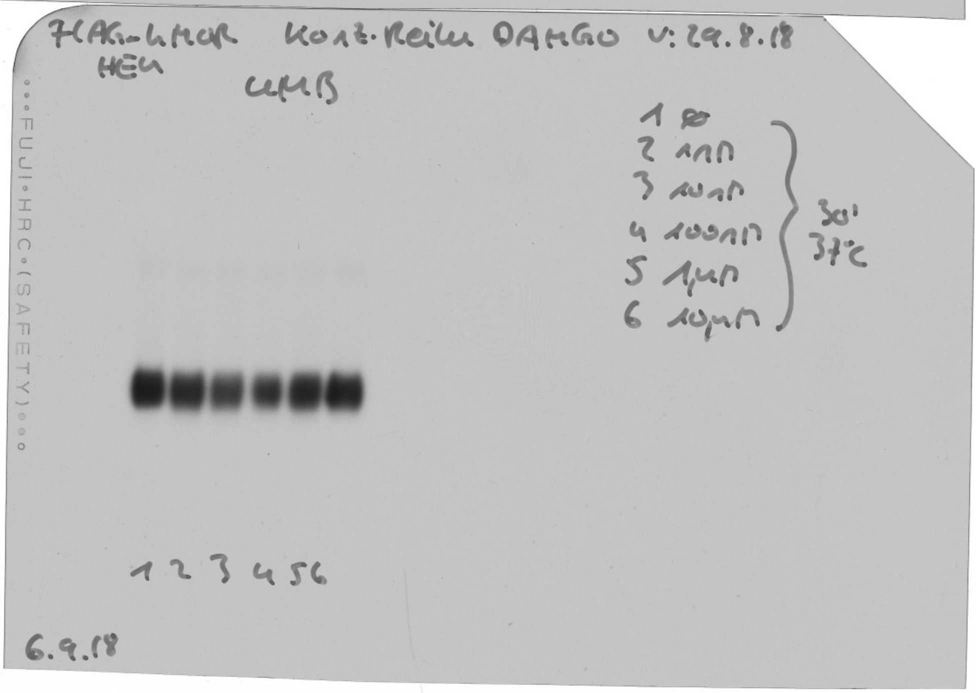
FLAG-hMOR
HEK
DAMGO
v: 29.8.18
1 ø
2 1nM
3 10nM
4 100nM
5 1µM
6 10µM
30'
37°C
1 2 3 4 5 6
6.9.18
FUJI•HRC•(SAFETY)

Supplementary figure 1. Uncropped images of western blots depicted in figure 4. For NOP, the rabbit polyclonal phosphosite-specific anti-S346 7TM0320A (previously known as 5034), anti-pS351 7TM0320B (previously known as 4876) and anti-pT362/S363 7TM0320C (previously known as 4874) as well as the phosphorylation-independent anti-NOP 7TM0320N (previously known as 4871) antibodies were used. The monoclonal phosphorylation-independent anti-MOP receptor antibody 7TM0319N (previously known as UMB-3) and phosphosite-specific antibodies anti-pT370 7TM0319B1 (previously known as 3196), anti-pS375 7TM0319C (previously known as 2493), anti-pT376 7TM0319D (previously known as 3723) and anti-pT379 7TM0319E (previously known as 3686) were used for MOP. The cropped parts of the blots used in figure 4 are depicted in red boxes.
